# Identification and expression analysis of the cysteine synthase (*CSase*) gene family in *Brassica napus* L. under abiotic stress

**DOI:** 10.1186/s12870-025-06532-8

**Published:** 2025-06-05

**Authors:** Tianyuan Xue, Yuqing He, Yudi Gao, Zhiheng Lei, Jingdong Chen, Shuai Yin, Xigang Dai, Changli Zeng, Heping Wan

**Affiliations:** Hubei Engineering Research Center for Protection and Utilization of Special Biological Resources in the Hanjiang River Basin, College of Life Science, Jianghan University, Wuhan, 430056 Hubei China

**Keywords:** *Brassica napus* L., Cysteine synthase, Gene family, Abiotic stress

## Abstract

Cysteine is the first organic compound identified in plants that contains both sulfur and nitrogen. It serves as a precursor for sulfur-containing metabolites such as methionine, glutathione (GSH), and Fe-S clusters, all of which play crucial roles in plant growth, development, and stress responses. Cysteine synthase (CSase) catalyzes the final step in cysteine biosynthesis; therefore, studying the *CSase* gene family is essential for understanding its role in plant abiotic stress tolerance. Using the CSase protein sequences of *Arabidopsis thaliana* as seed sequences and integrating protein domain information, 69 members of the *BnCSase* gene family were identified from the whole genome of *Brassica napus* ZS11. These members were analyzed for their physicochemical properties, phylogenetic relationships, covariance relationships, protein-protein interaction (PPI) networks, associated miRNAs, and SNP variations. Based on transcriptome data, the expression patterns of *BnCSase* genes under different abiotic stress treatments were investigated. Furthermore, the relative expression levels of several *BnCSase* genes were analyzed under salt, alkali, low nitrogen, and drought stress treatments at 0, 6, 12, and 24 h using qRT-PCR to explore their roles in abiotic stress tolerance in *B. napus*. The results revealed distinct expression patterns of *BnCSase* genes in response to different abiotic stress signals, indicating stress-specific responses in *B. napus.* This study provides a theoretical basis for elucidating the functions and molecular genetic mechanisms of the *BnCSase* gene family in abiotic stress tolerance in rapeseed.

**Supplementary Information:**

The online version contains supplementary material available at 10.1186/s12870-025-06532-8.

## Introduction

In the context of climate change and global population growth, crops are increasingly challenged by abiotic stresses, including temperature fluctuations, drought, salinity, and heavy metal contamination. These stresses adversely affect crop growth, ultimately leading to reductions in both yield and quality [1]. Sulfur is an essential macronutrient for plant growth and development, involved in various physiological and metabolic processes [2]. Plants absorb sulfur from the environment in the form of sulfate through their roots, which is then reduced to sulfide and ultimately incorporated into the first sulfur-containing organic compound, cysteine [3]. Cysteine is the first organic compound identified in plants that contains both sulfur and nitrogen. It serves as a precursor for various sulfur-containing metabolites, including methionine [4], glutathione (GSH), and Fe-S clusters, all of which play crucial roles in plant growth, development, and stress response [5, 6, 7]. Additionally, cysteine serves as a biosynthetic precursor for numerous essential molecules, including vitamin B1 [8], coenzyme A (CoA) [9], glucosinolates [10], and thioredoxins [11].

Cysteine synthesis is catalyzed by cysteine synthase (CS), also known as O-acetylserine(thiol)lyase (OASTL). OASTL participates in the final step of cysteine synthesis by catalyzing the reaction between hydrogen sulfide (H₂S) and O-acetylserine (OAS) to produce cysteine [12, 13]. The cysteine synthase gene family was first reported in Arabidopsis thaliana, consisting of 9 family members that can be classified into 5 subfamilies. All members contain the conserved pyridoxal 5’-phosphate (PLP) binding site [14]. OAS-A1, located in the cytoplasm, OAS-B in plastids, and OAS-C in mitochondria, exhibit higher expression levels compared to other family members. These enzymes interact with serine acetyltransferase (SAT) [15, 16, 17]. In contrast, CYS-D1 and CYS-D2, which are also localized in the cytoplasm, show relatively low expression levels and are considered to be less active cysteine synthases [14]. The interaction between serine acetyltransferase and cysteine synthase plays a critical role in cysteine biosynthesis; therefore, the ability to interact with SAT is considered a key feature of cysteine synthase (CSase) [17, 18]. The cysteine synthase family is a functionally diverse protein family. In Arabidopsis, CYS-C1, a member of the cysteine synthase family, exhibits β-cyanoalanine synthase (CAS) activity and is involved in the plant cyanide detoxification process. It catalyzes the conversion of cyanide and cysteine into β-cyanoalanine and hydrogen sulfide, thereby mitigating the harmful effects of cyanide on plants [14]. The cytosolic protein DES1 in Arabidopsis is a novel cysteine desulfhydrase (CDes) that catalyzes the desulfuration of cysteine, releasing hydrogen sulfide, ammonia, and pyruvate. This process enhances the plant’s antioxidant defense capacity and improves its tolerance to environmental conditions that induce oxidative stress [19]. Arabidopsis protein SCS exhibits S-sulfocysteine synthase activity and is localized to the thylakoid. The loss of SCS function significantly reduced three key photosynthetic parameters in Arabidopsis, including net CO₂ assimilation rate, mesophyll conductance, and mitochondrial respiration under dark conditions [20]. Recent studies have shown that the *CSase* gene plays an important role in stress responses. For example, the expression of the *Brassica napus* DES1 protein is downregulated under cadmium stress, leading to a decrease in cysteine desulfhydrase activity, which results in an increase in total cysteine content. This, in turn, enhances the production of sulfur-containing peptides involved in heavy metal sulfur metabolism, thereby improving plant tolerance to heavy metals. In contrast, under high salinity conditions, the *Brassica napus* DES1 protein catalyzes the desulfuration of cysteine, releasing hydrogen sulfide, ammonia, and Pyruvicacid, which enhances the plant’s antioxidant defense capacity [21]. The overexpression of the soybean *GmOASTL4* gene in tobacco resulted in a significant increase in OASTL activity and cysteine levels in the transgenic plants, which enhanced their tolerance to cadmium stress [22].

Rapeseed, particularly kale-type rapeseed (*Brassica napus* L.), is globally recognized as one of the most significant oilseed crops due to its extensive cultivation area. As an important economic crop, rapeseed plays a crucial role not only in providing raw materials for the human edible oil industry but also in various other industries. Furthermore, rapeseed seeds are rich in oil content, making them a valuable source of oil [23]. The *CSase* gene family has been identified in *Arabidopsis thaliana* [14], *Solanum lycopersicum* L [24]., *Sorghum bicolor* [25], *Setaria italica* (L.) P. Beauvois [26], *Medicago sativa* L [27]., and *Triticum aestivum* L [28]. Xie et al., in their study on the Yangyou 6 cultivar, employed RT-PCR to clone a cDNA encoding an O-acetyl-L-serine(thiol)lyase homolog with DES activity from seedling roots of *B. napus*. Biochemical analysis of the purified protein demonstrated that it primarily catalyzes L-cysteine degradation, displaying significant DES activity, and was subsequently designated as *BnDES1* [21]. However, this study did not involve a comprehensive genome-wide identification of *CSase* gene family members in *B. napus*.

In this study, we conducted a genome-wide analysis of *B. napus* using bioinformatics methods, identifying 69 *CSase* genes, which were classified into six subfamilies. We performed a comprehensive analysis of the physicochemical properties, phylogenetic relationships, chromosomal localization, and intron-exon structures of these *CSase* genes. Additionally, further bioinformatics analyses revealed protein interactions as well as inter- and intra-species collinearity. The study also included a variation analysis of the *CSase* gene family in *B. napus.* Transcriptomic data further uncovered the expression profiles of *CSase* genes under various abiotic stress conditions.

Although previous studies have explored the role of *CSase* genes in enhancing abiotic stress resilience through cysteine synthesis in *B. napus* and *M. sativa*, the expression profiles of *CSase* genes in *B. napus* under abiotic stress conditions remain underexplored. In this study, we used qPCR to analyze the expression of *BnCSase* family members in response to salt (NaCl), drought (PEG6000), low nitrogen (Table 2), and alkali (0.2% NaHCO_3_) stresses at four time points (0, 6, 12, and 24 h). This investigation provides evidence of *BnCSase* gene responsiveness to abiotic stress. The results presented here establish a basis for further research on the functional roles and mechanisms of *CSase* genes in abiotic stress tolerance in *B. napus* and offer a theoretical foundation for the selection and breeding of *B. napus* germplasm with enhanced abiotic stress resilience.

**Table 1:** Number of CSase proteins contained in each group in the phylogenetic tree

Group	CSase family	CysA family	CysB family	CysC family	CysD family	SCS family
AtCSase Number	0	2	2	1	3	1
BnCSase Number	36	6	6	5	14	2
BrCSase Number	18	3	3	2	5	1
BoCSase Number	21	3	3	3	5	1

## Results

### Identification and chromosomal localization of CSase gene family

**Fig. 1:**
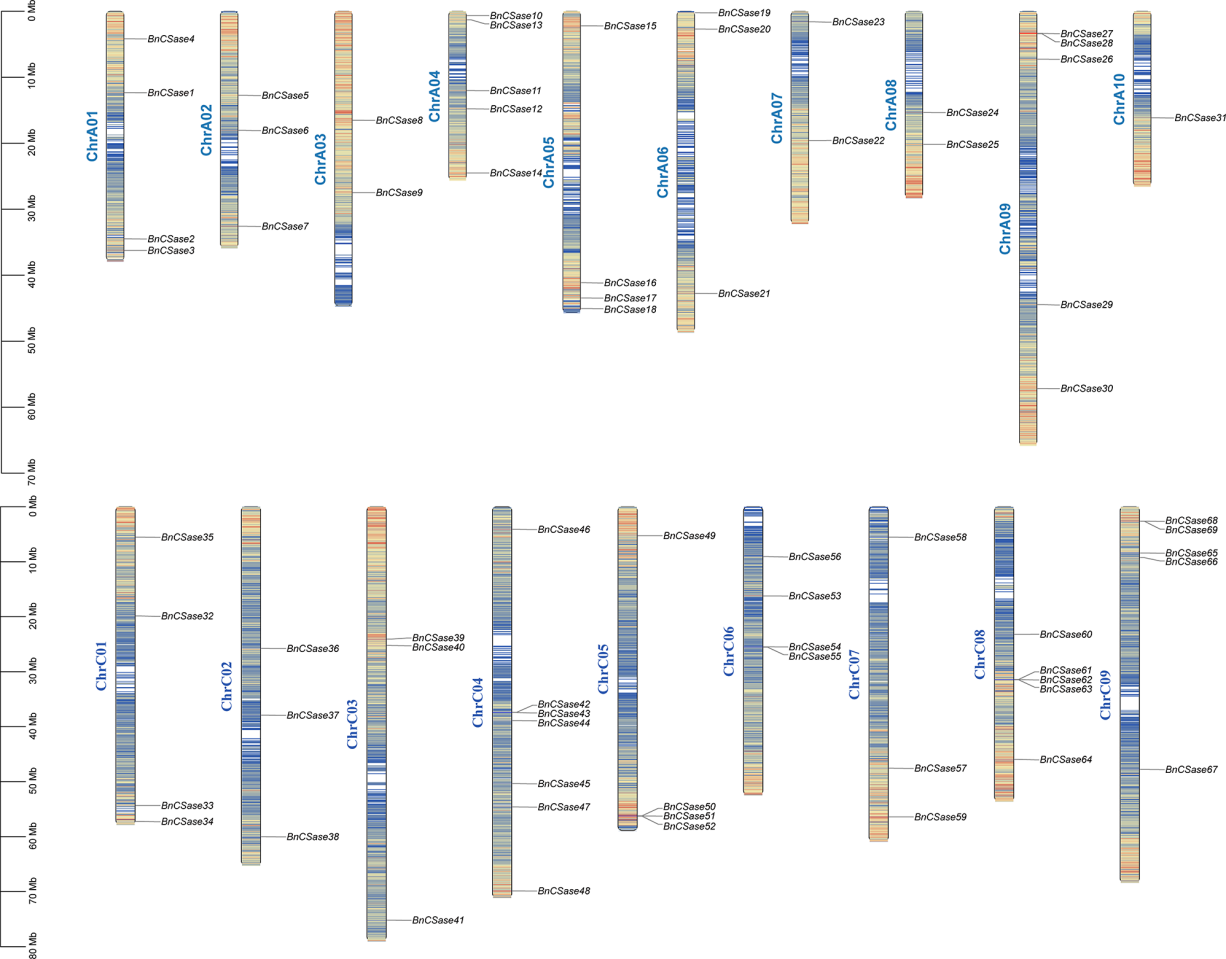
Chromosomal location of the *BnCSases* in the *B. napus* genome

The conserved domain seed file for CSase (PF00291) was downloaded from the Pfam database. HMMER software was subsequently employed on a Linux system to construct a Profile HMM for comparative analysis with B. napus protein sequences, aiming to remove redundant sequences. Candidate genes of high quality were selected based on an E-value threshold of < 1 × 10⁻¹⁰, leading to the identification of BnCSase protein sequences. Cross-referencing the obtained results with HMMER screening outcomes using the NCBI CD-search tool, 69 members of the *BnCSase* gene family were ultimately identified and named sequentially as *BnCSase1* to *BnCSase69* according to their chromosomal order. These gene family members were localized on 19 chromosomes (Fig. 1). Specifically, seven BnCSase genes were mapped to chromosome C4, and six to chromosome C8. Additionally, five *BnCSase* genes were localized on chromosomes A4, A9, and C9, respectively. Four *BnCSase* genes were identified on chromosomes A1, A5, C1, C5, and C6, respectively. Furthermore, three *BnCSase* genes were found on chromosomes A2, A6, C2, C3, and C7, respectively. Two *BnCSase* genes were mapped to chromosomes A3, A7, and A8, respectively, while one *BnCSase* gene was localized on chromosome A10.

### Physicochemical properties and subcellular localization prediction of BnCSase proteins

Through predictive analysis, the amino acid (AA) length of BnCSase proteins was found to range from 121 to 607, with relative molecular weights (MW) spanning from 13.01 to 66.22 kDa and theoretical isoelectric points (pI) varying between 4.86 and 9.54 (Table S1). Subcellular localization predictions revealed that 32 CSase proteins are likely localized in the chloroplast, 22 in the cytoplasm, and 15 in the mitochondria.

### Phylogenetic analysis of BnCSase proteins

**Fig. 2:**
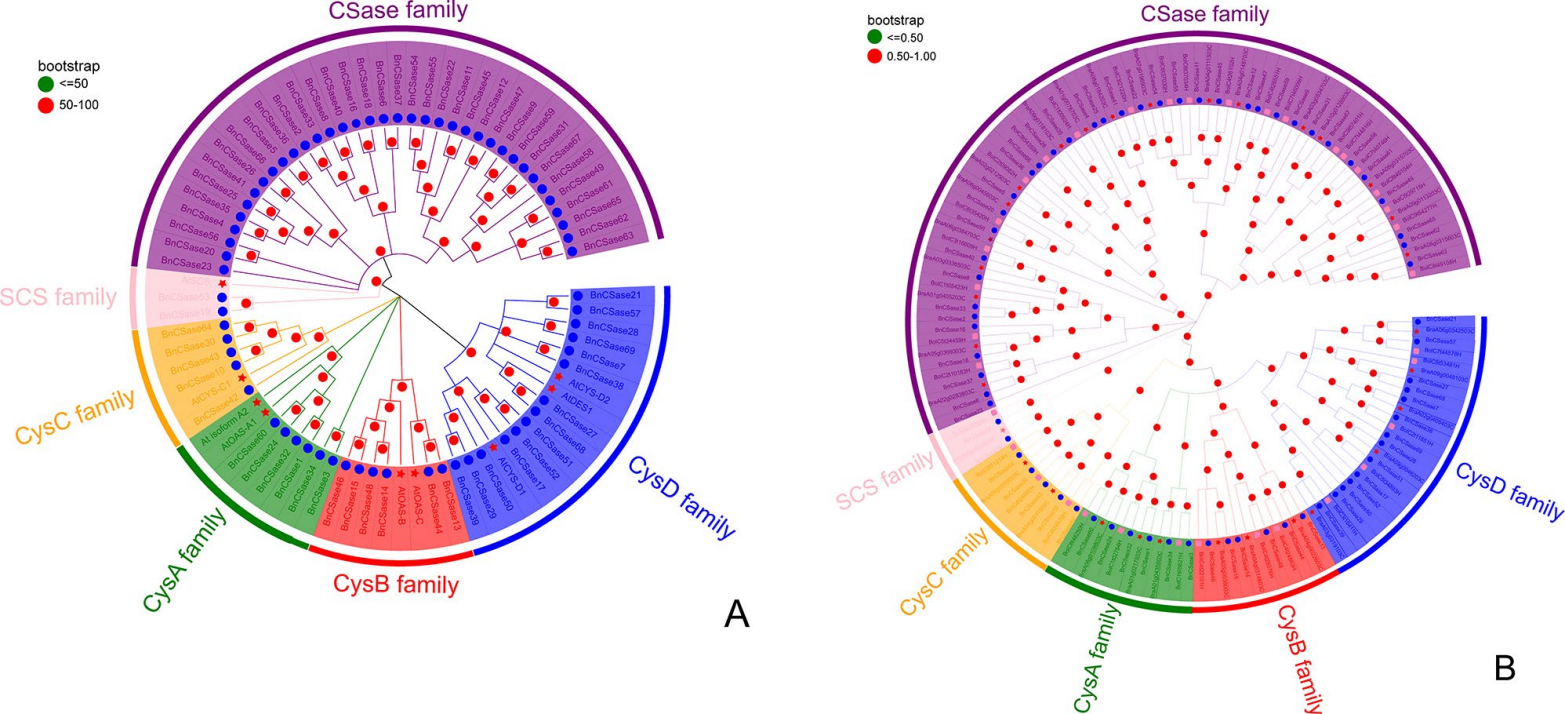
Phylogenetic tree of CSase proteins. **(A)** Phylogenetic tree of CSase proteins in *B. napus* and *(A) thaliana*. **(B)** Phylogenetic tree of CSase proteins in *(B) napus*, *B. rapa*, and *B. oleracea*

To explore the phylogenetic relationships among CSase proteins, a phylogenetic tree was constructed by combining 69 CSase proteins from *B. napus* with 8 CSase proteins from *Arabidopsis thaliana*. Based on their genetic relationships and the positioning of the AtCSase proteins within the tree, the CSase proteins were classified into six groups: CSase family, SCS family, CysC family, CysA family, CysB family, and CysD family (Fig. 2A). The distribution of proteins within these groups varied, with the CSase family containing the largest number of proteins, including 36 BnCSase. The CysD family comprised 3 AtCSase and 14 BnCSase, the CysC family included 1 AtCSase and 5 BnCSase, the CysA family consisted of 2 AtCSase and 6 BnCSase, and the CysB family contained 2 AtCSase and 6 BnCSase. The SCS family had the fewest proteins, comprising only 1 AtCSase and 2 BnCSase (Table 1).

**Table 2:** Formulation of low nitrogen nutrient solutions

Chemical Reagent	Mother Liquor (g/L)	Low Nitrogen added nutrition (ml/L)^1^
KNO_3_	102	0.3
MgSO_4_.7H_2_O	98	5
KH_2_PO_4_	28	5
Ca(NO_3_)_2_.4H_2_O	236	0
FeSO_4_.7H_2_O	2.799	5
EDTA-2Na	3.722	5
MnCl_2_.4H_2_O	3.62	0.25
ZnSO_4_.7H_2_O	0.44	0.25
CuSO_4_.5H_2_O	0.16	0.25
H_3_BO_3_	5.72	0.25
Na_2_MoO_4_·4H_2_O	0.18	0
CaCl_2_	111	4.5

To further confirm the phylogenetic relationships of CSase proteins in Brassicaceae species, 32 CSase proteins from *Brassica rapa* and 36 CSase proteins from *Brassica oleracea* were identified using HMMER and NCBI-CDsearch methods. These proteins were also used to construct a phylogenetic tree (Table S2, Fig. 2B). The analysis revealed that these proteins could similarly be classified into four groups, and the grouping of BnCSase remained consistent, verifying the reliability of the phylogenetic tree. Notably, the CSase family contained the highest number of proteins, including 18 BrCSase and 21 BoCSase, followed by the CysD family, which contained 5 BrCSase and 5 BoCSase. The CysB family included 3 BrCSase and 3 BoCSase, while the CysA family included 3 BrCSase and 3 BoCSase. The CysC family contained 2 BrCSase and 3 BoCSase (Table 1, Figure S1). The SCS family contained the fewest proteins, with only 1 BrCSase and 1 BoCSase.

### Analysis of BnCSase family structure, conserved motifs and promoter cis-acting elements

Conserved motif analysis is a critical tool for understanding the function, structure, and evolutionary relationships of proteins. The conserved motif analysis of BnCSase proteins revealed that Motif2 and Motif3 are present in all BnCSase proteins, indicating their essential roles. Motif9 and Motif10 are specific to the CSase family, with no occurrences in other subfamilies. Motif1 is found across various subfamilies, though it is absent in most BnCSase proteins within the CSase family. Motif4 is missing in BnCSase21 from the CysD family and in some CSase proteins within the CSase family. Similarly, Motif5 is absent in BnCSase57 from the CysD family and in a few CSase family proteins. Motif6 is entirely absent in all CSase family proteins.

**Fig. 3:**
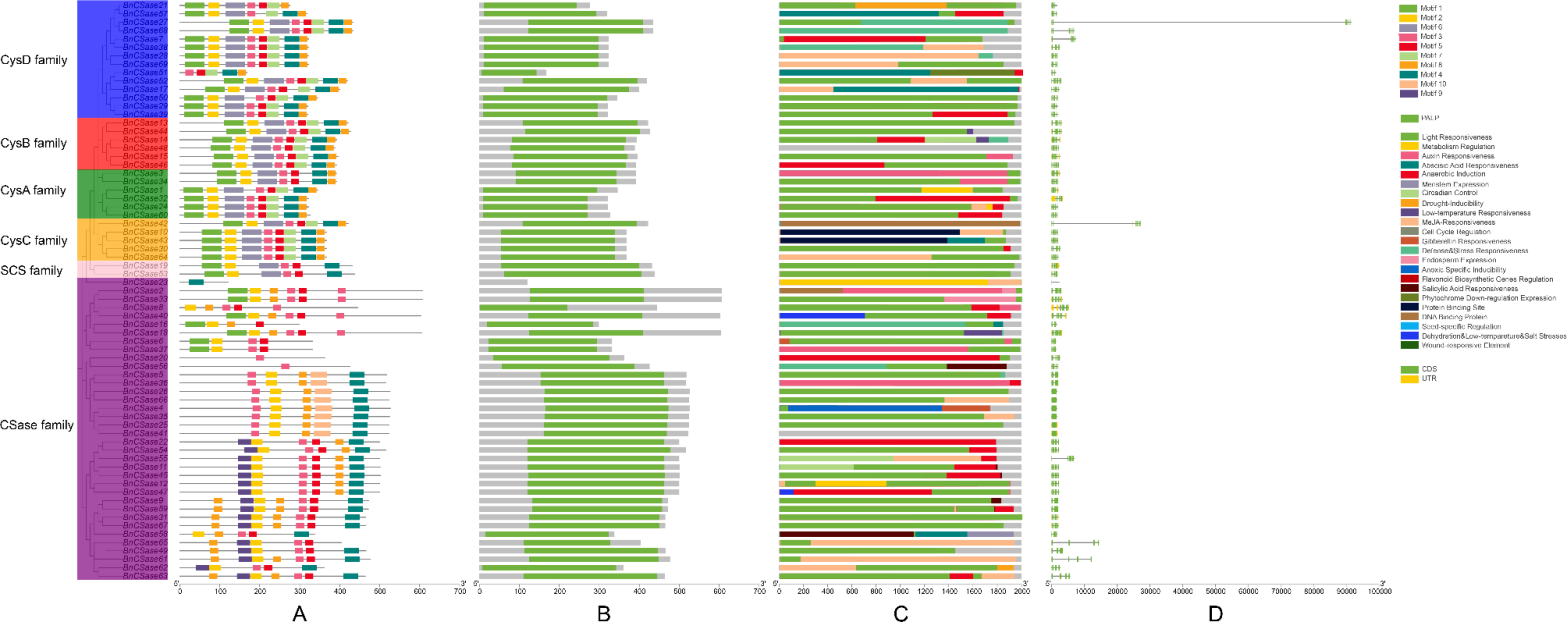
Gene structure analysis of *CSase* family in *B.napus*. **(A)** Conserved motifs of BnCSase family proteins. **(B)** Pfam structure of BnCSase family proteins. **(C)** Promoter cis-acting element of BnCSases. **(D)** The mRNA structure encoded by the *BnCSases*

**Fig. 4:**
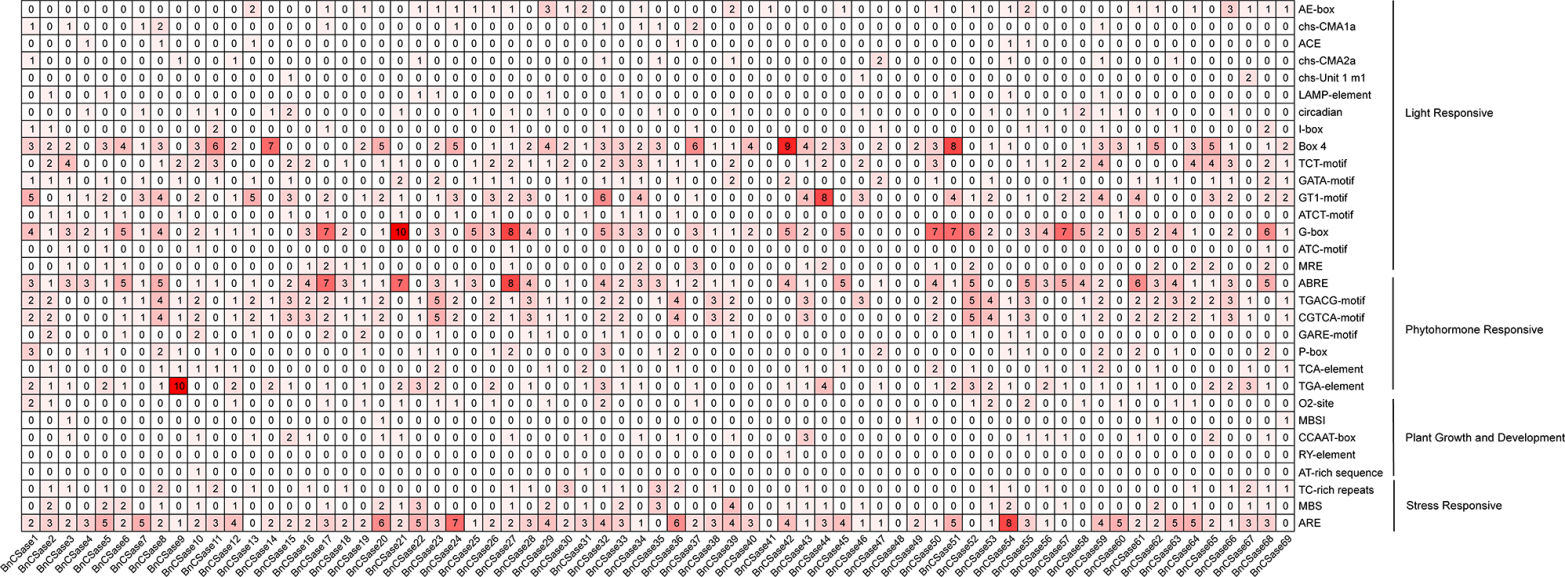
Classification of Cis-acting Elements in the Members of the *BnCSase* Gene Family

Furthermore, Motif7 is absent in BnCSase3 and BnCSase34 from the CysA family, as well as in all SCS and CSase family proteins. Motif8 is missing in BnCSase6, BnCSase20, BnCSase37, and BnCSase56 from the CSase family and in all SCS family proteins. Motif9 is exclusively present in some CSase proteins within the CSase family and does not appear in other subfamilies. Similarly, Motif10 is restricted to certain CSase proteins within the CSase family (Fig. 3A). This specificity suggests that Motif9 and Motif10 are unique conserved sequences in BnCSase proteins. Structural analysis revealed that all BnCSase proteins contain the PALP domain, a characteristic feature of this protein family (Fig. 3B). Promoter cis-acting element analysis suggested that BnCSase proteins are potentially involved in environmental stress responses, such as drought, low temperature, salt stress, pathogen attack, and mechanical damage, as well as in hormone signal responses (Fig. 3C). Among these elements, light-responsive elements were the most abundant, with a total of 763, far exceeding other types of cis-acting elements. Notably, *BnCSase32* had the highest number of cis-acting elements, reaching 44, indicating its potential role in the growth and development of rapeseed. Analysis of the mature mRNA structures of BnCSase proteins showed that these proteins contain between 1 and 13 coding sequences (CDS). For example, the BnCSase27 protein contains 8 CDS regions, located between 90,000 bp and 100,000 bp at the 3’ end of the sequence. Some BnCSase proteins also include 1 to 3 untranslated regions (UTRs) (Fig. 3D). Based on the analysis of the promoter regions of *BnCSase* gene family members using the PlantCARE online tool, a total of 103 cis-acting elements were identified. The following is a statistical summary of the cis-acting elements related to growth and development, stress response, plant hormones, and light response (Fig. 4). Among them, six elements are associated with growth and development, including the O2-site involved in zein protein metabolism regulation and the CCAAT-box, which regulates various cellular processes. Three elements are related to stress response, including the MYB-binding site MBS, which is involved in drought induction, TC-rich repeats associated with disease resistance and stress response, and the ARE element involved in anaerobic induction. Seven plant hormone-related cis-acting elements were identified, including ABRE, which regulates ABA signaling, the TCA-element for salicylic acid response, and the CGTCA-motif, which is involved in jasmonic acid response. The most abundant cis-acting elements are those related to light response, with a total of 16 elements, including the MRE that regulates target gene expression, the G-box, a common regulatory element responding to external environmental stimuli, and the Box, a conserved DNA module associated with light response.

### Collinearity analysis of CSase gene family

**Fig. 5:**
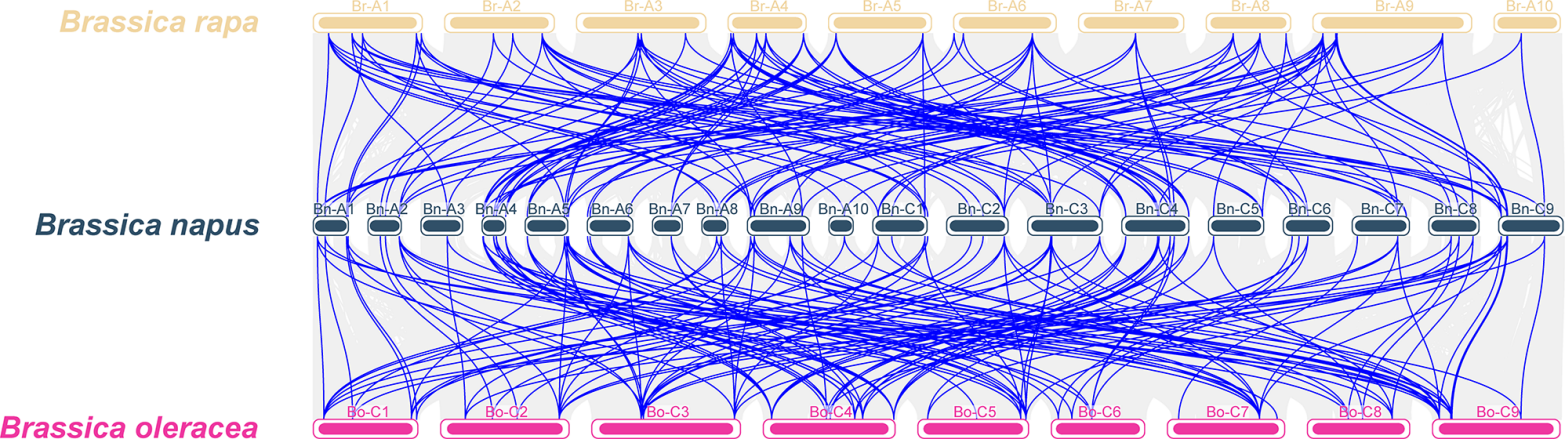
Collinearity of *CSase* genes in *B. napus*, *B. rapa*, and *B. oleracea*

**Fig. 6:**
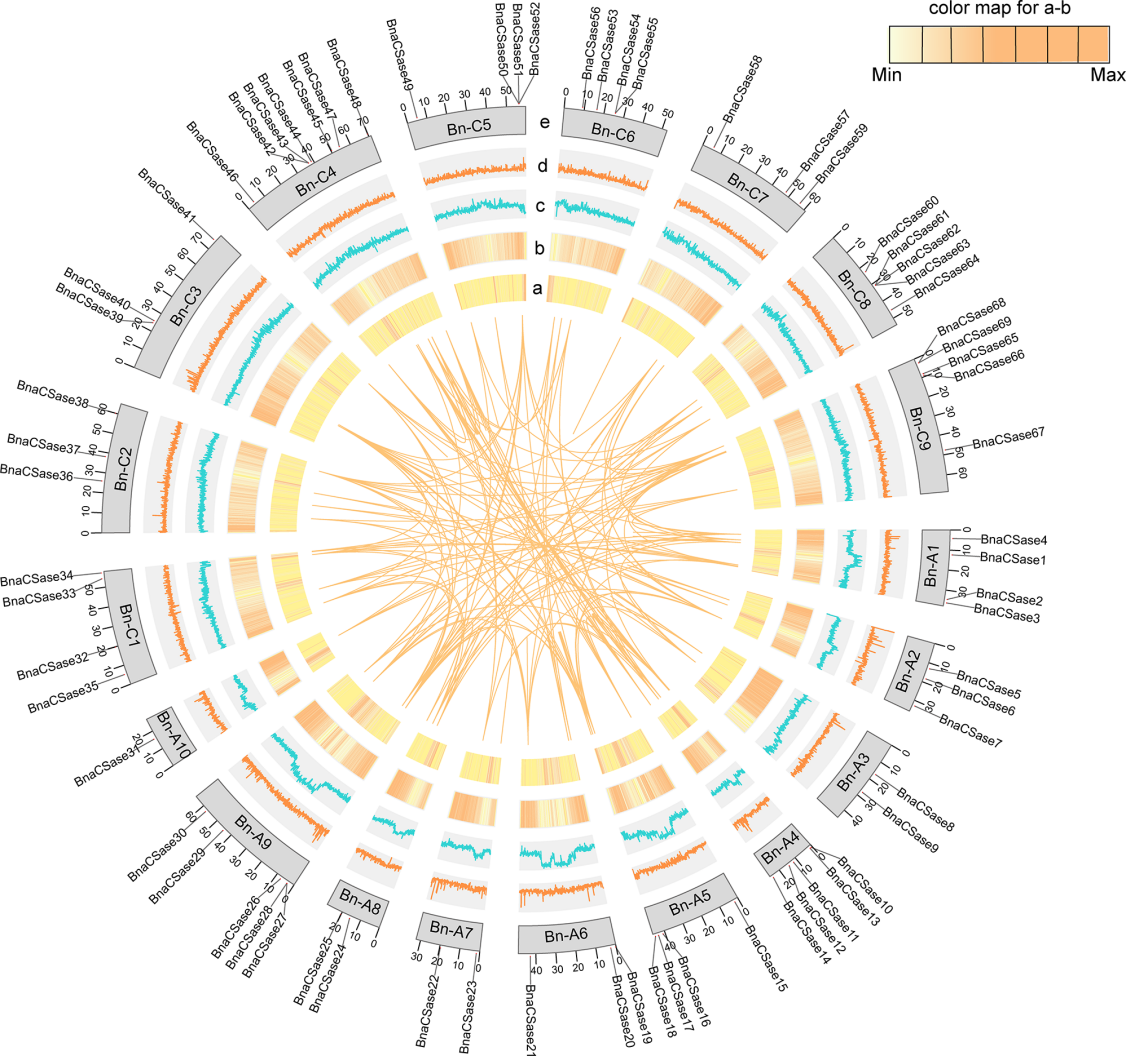
Collinearity of *BnCSases*. The circles in the figure from inside to outside represent the unknown base **(a)** N ratio, **(b)** gene density, **(c)** GC ratio, **(d)** GC skew, and **(e)** chromosome length of the *B. napus* genome

**Fig. 7:**
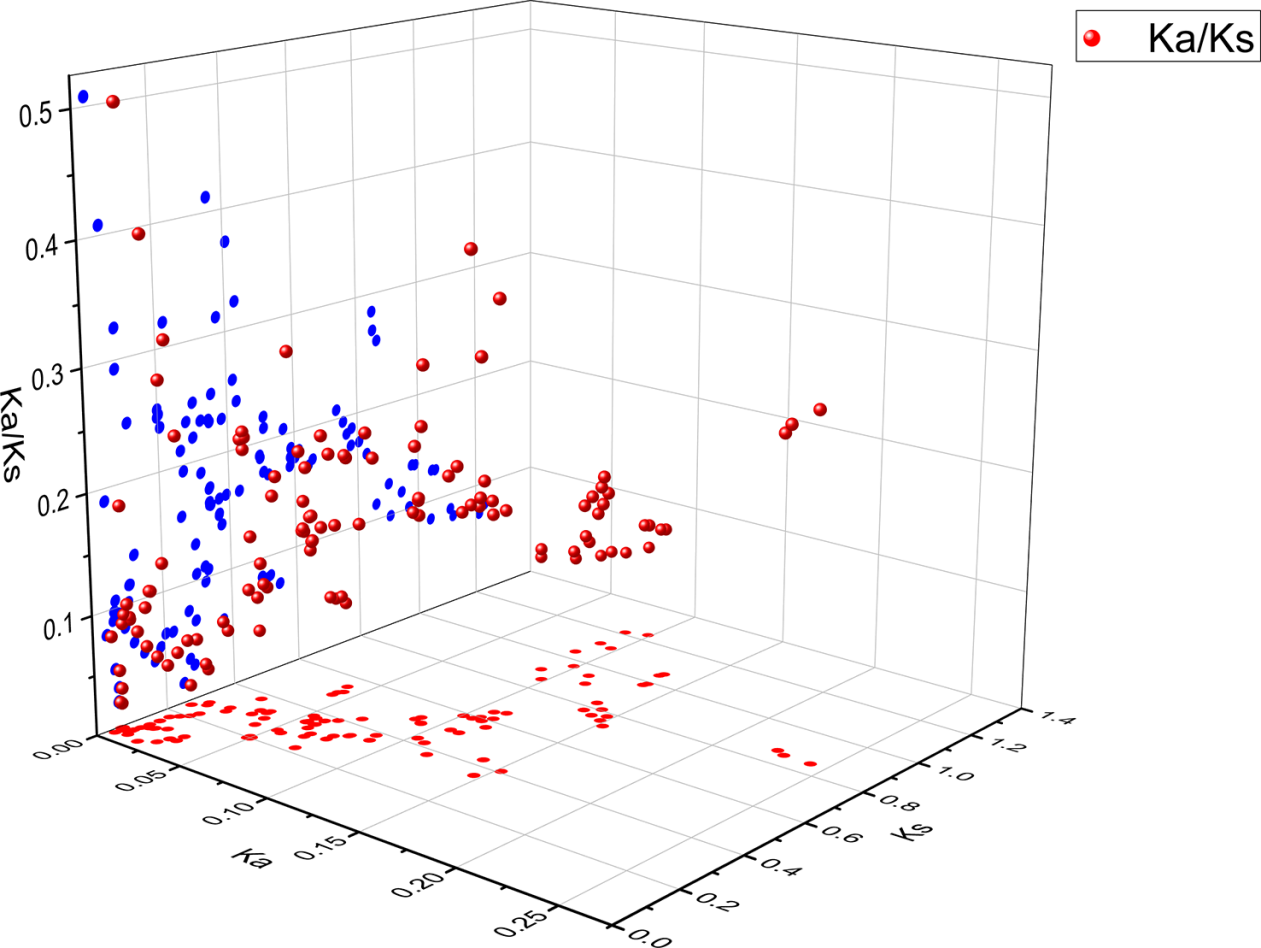
The selective evolutionary pressure on *BnCSases*. Blue dots represent the Ka/Ks values within *BnCSase* genes, and red dots represent the corresponding Ka and Ks values of *BnCSase* genes within species

To investigate the collinearity of *CSase* genes, a collinearity analysis was conducted among *Brassica napus* (oilseed rape), *Brassica rapa* (Chinese cabbage), and *Brassica oleracea* (cabbage) (Fig. 5). The results showed 139 pairs of collinear genes between *B. napus* and *B. rapa CSase* genes, and 147 pairs of collinear genes between B. napus and *B. oleracea CSase* genes. However, *BnCSase23*, *BnCSase43*, *BnCSase51*, *BnCSase55*, *BnCSase62*, and *BnCSase63* exhibited no collinearity with *B. rapa* or *B. oleracea* (Fig. 4 and Table S3). Further analysis of collinearity within the *B. napus* genome revealed 148 pairs of collinear genes related to *BnCSase* (Fig. 6 and Table S4), including 122 collinear gene pairs among *BnCSase* genes. To better understand the genetic relationship between these collinear gene pairs, Ka/Ks ratios were calculated to assess selection pressure (Fig. 7, Table S5). The results indicated that all 122 collinear gene pairs had Ka/Ks values less than 1, suggesting they were subject to purifying selection.

### Protein–protein interaction networks analysis

**Fig. 8:**
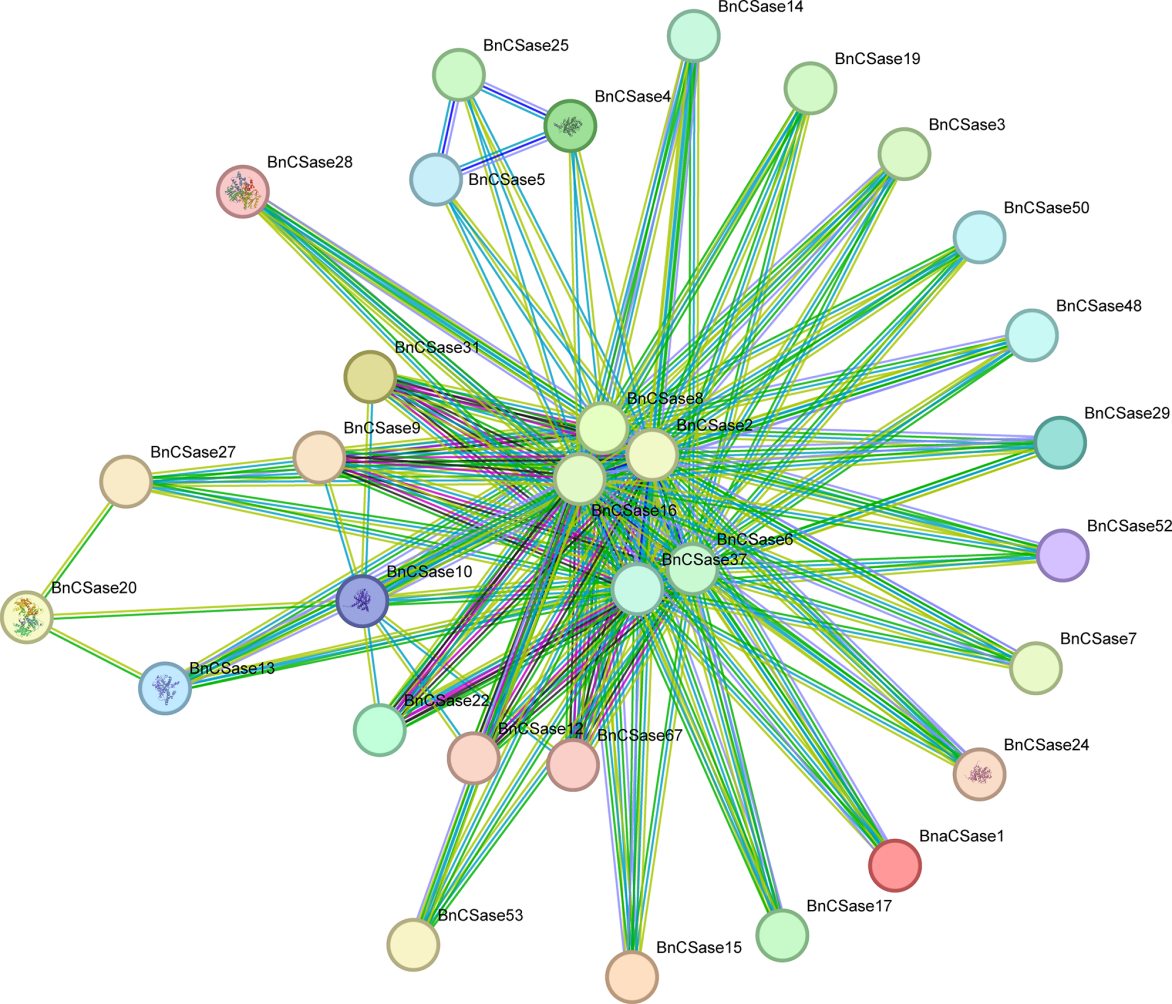
BnCSase proteins interaction network analysis

The PPI analysis revealed that a total of 32 BnCSase proteins were involved in interactions, resulting in 270 protein-protein interactions. Notably, BnCSase2, BnCSase8, and BnCSase16 exhibited the highest levels of interaction, each interacting with 28 BnCSase proteins. In contrast, BnCSase proteins located in the outer circle displayed fewer interactions (Fig. 8). It is worth mentioning that BnCSase4, BnCSase10, BnCSase13, BnCSase20, BnCSase24, and BnCSase28 were successfully matched to their corresponding tertiary protein structures in the STRING database (Figure S2).

### Analysis of interaction between MicroRNA and BnCSases target genes

**Fig. 9:**
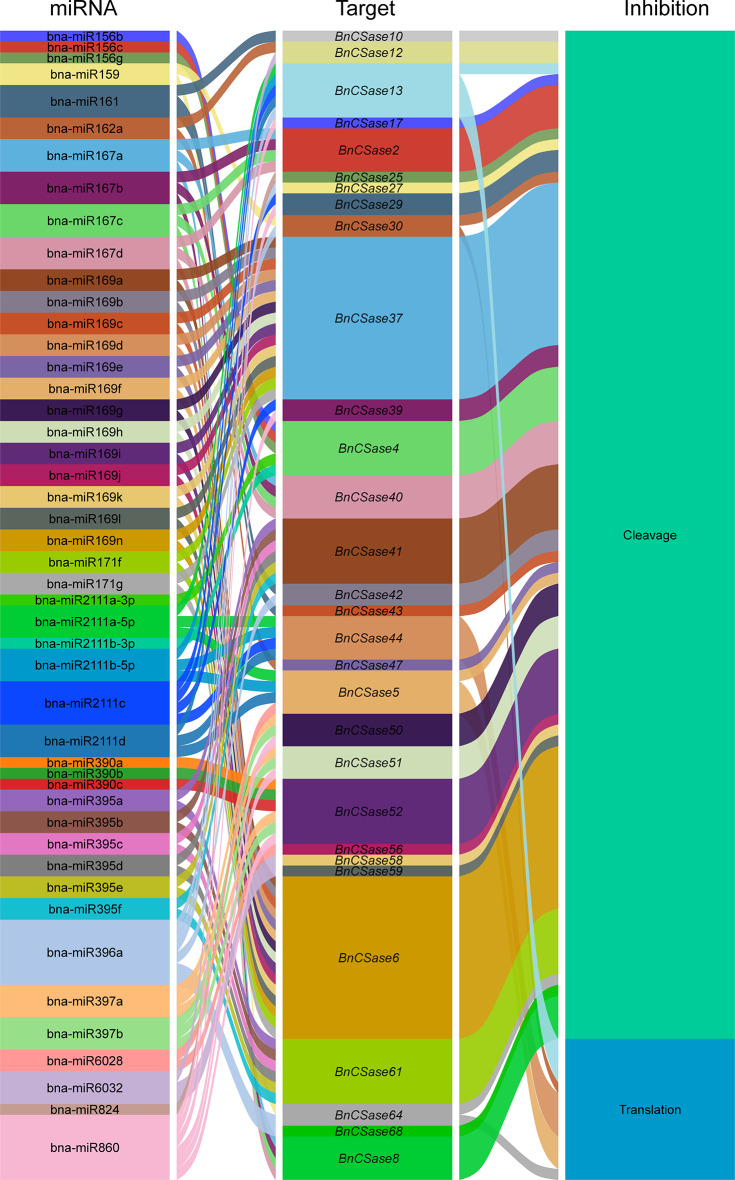
Analysis of the interaction between microRNA and *BnCSases*

The Sankey diagram illustrates the interactions among miRNAs, *BnCSase* genes, and their regulatory effects, including the regulatory relationships between *BnCSase* gene targets and post-transcriptional inhibition mechanisms. Each miRNA and its corresponding target gene are represented by distinct colors. A total of 47 miRNAs were identified to target multiple *BnCSase* genes, resulting in 106 distinct interactions (Fig. 9). The findings indicate that a single miRNA can target multiple *BnCsase* genes, while certain transcripts can also be targeted by multiple miRNAs, reflecting the complexity of the regulatory network.Notably, genes such as *BnCSase6* and *BnCSase37* act as key regulatory hubs, interacting with several miRNAs, suggesting their central role in the network. The right column categorizes the regulatory effects into two mechanisms: Cleavage (green) and Translation Inhibition (blue). The analysis reveals that cleavage is the predominant mechanism underlying most miRNA-BnCSase interactions, whereas translation inhibition occurs less frequently.These results highlight the intricate and multifaceted nature of miRNA-mediated regulation of *BnCSase* transcripts, with cleavage serving as the primary mode of action.

### Analysis of transcriptome expression pattern of BnCSase

**Fig. 10:**
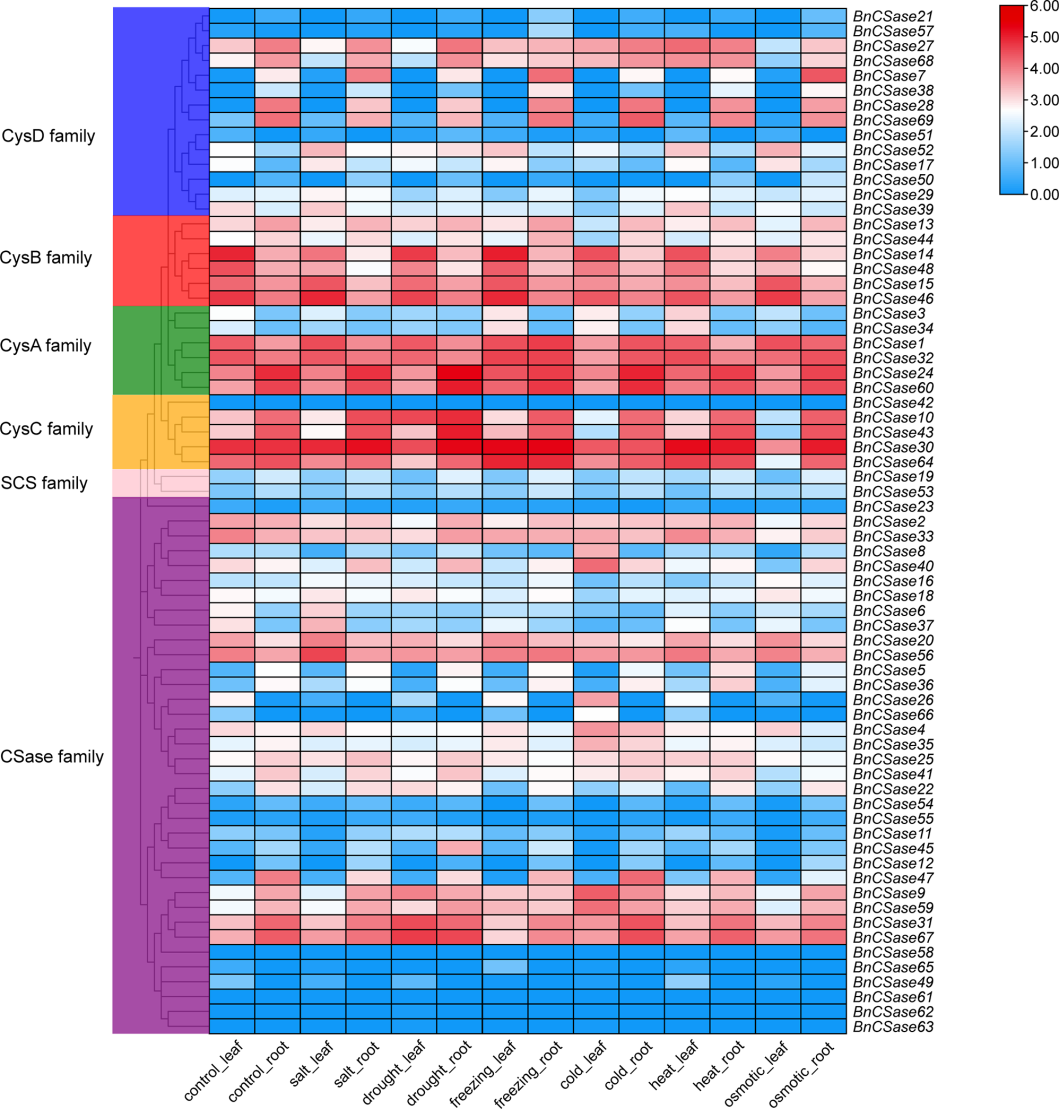
Analysis of expression patterns of *BnCSases* under abiotic stress treatments

To investigate the expression patterns of *BnCSase* genes under different abiotic stress conditions, transcriptome data (TPM values) of *BnCSases* in *B. napus* ZS11 were obtained for control (CK), salt, drought, freezing, cold, heat, and osmotic stress treatments. After data processing, expression heatmaps (log_10_(TPM + 1) were constructed (Fig. 10). The results revealed that certain BnCSases were either not expressed or expressed at low levels in both leaves and roots under different stresses. In contrast, genes such as *BnCSase1*, *BnCSase30*, *BnCSase32*, *BnCSase24*, *BnCSase60*, and *BnCSase30* displayed consistently high expression in both tissues across various stress conditions. Moreover, *BnCSase7*, *BnCSase27*, *BnCSase31*, *BnCSase67*, *BnCSase68*, *BnCSase28*, and *BnCSase69* exhibited high expression in roots under all stresses, with some also showing elevated expression in leaves under specific conditions. Additionally, *BnCSase14*, *BnCSase48*, *BnCSase15*, and *BnCSase46* were highly expressed predominantly in leaves across different stress treatments.

### Variation analysis of the CSase gene family in *B. napus*

**Fig. 11:**
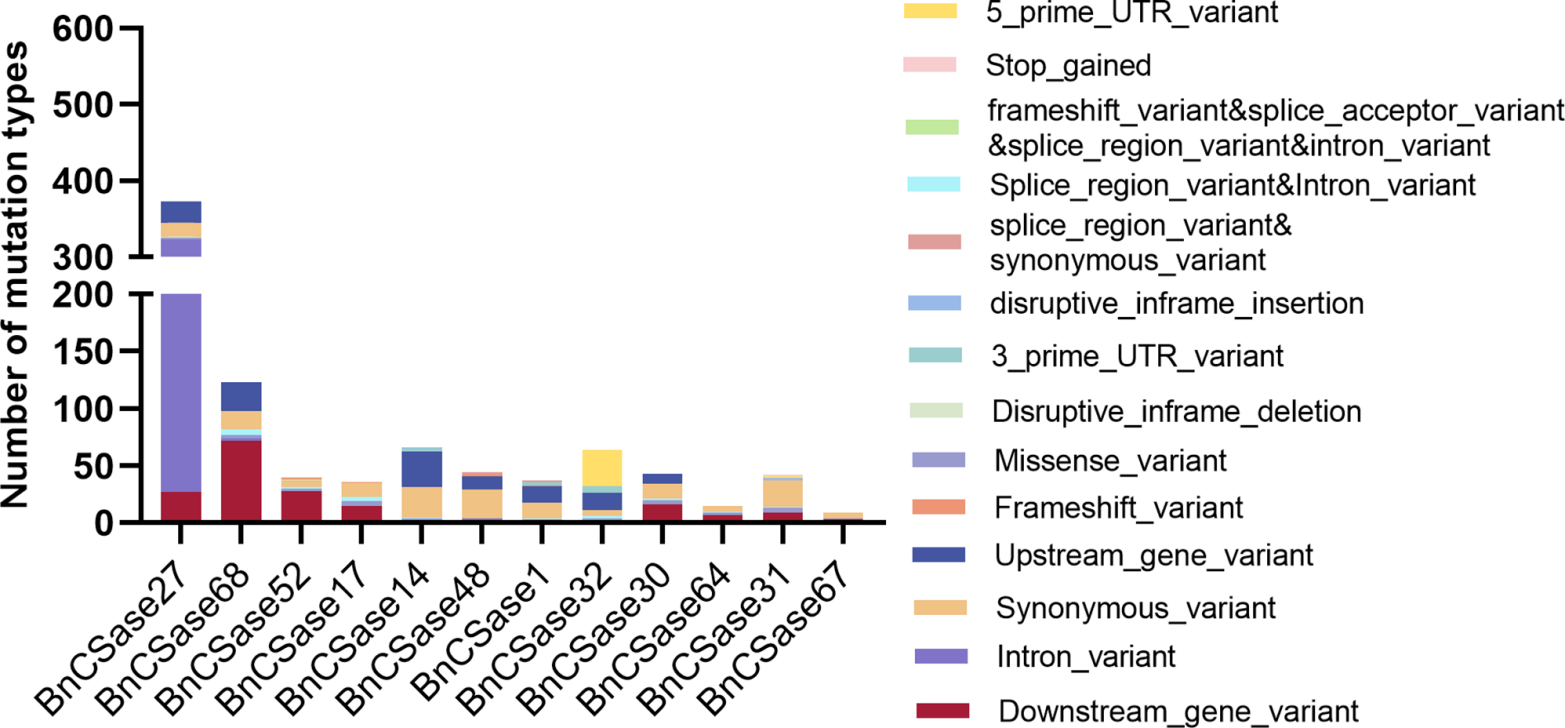
Statistics of *CSase* gene variation in *B. napus*

**Fig. 12:**
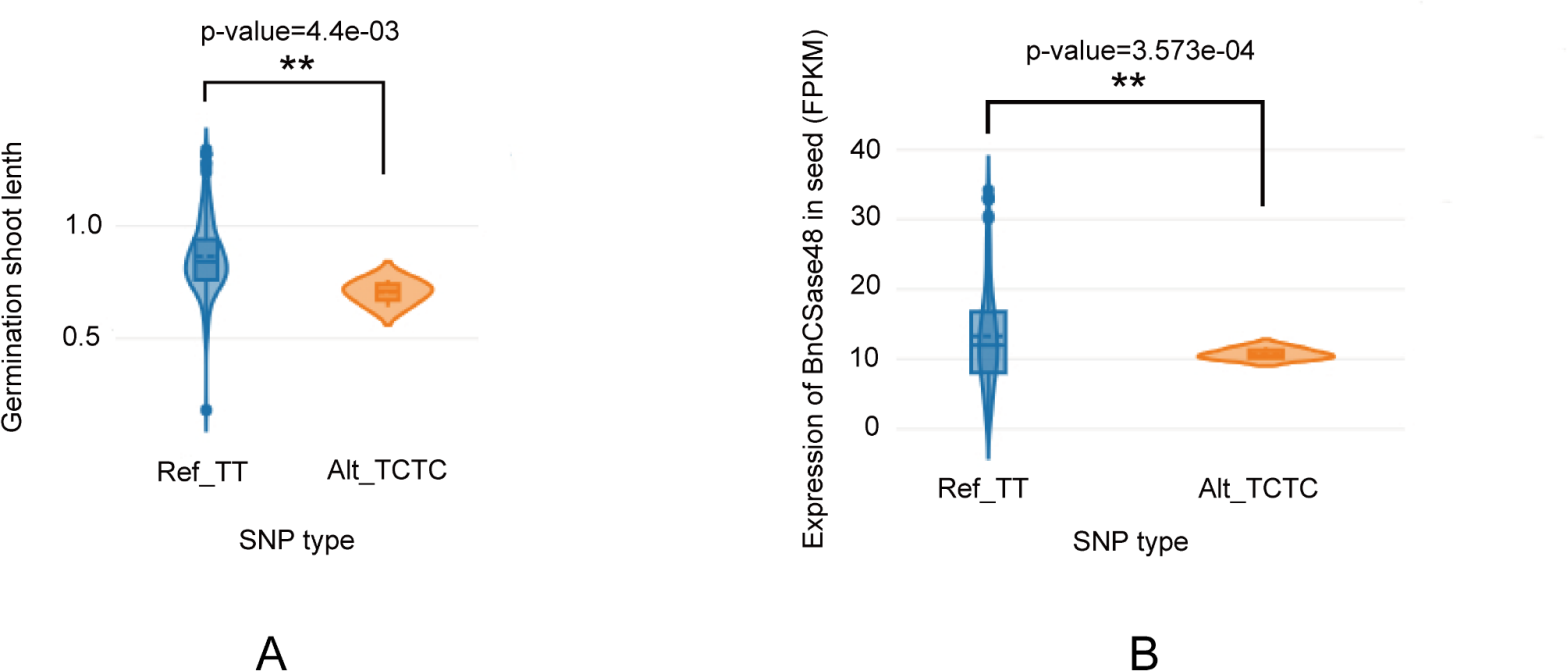
SNP variations in the *BnCSase48* frameshift mutant. **(A)** Germination shoot length under salt stress. **(B)** FPKM expression level of *BnCSase48* in seeds

The BnVIR database was employed to analyze SNP variations within the *CSase* gene family of *B. napus*. Certain SNP variations were identified in specific *BnCSase* genes, potentially contributing to differences in expression levels among family members. Based on the expression heatmap of *BnCSase* genes under abiotic stress, 12 highly expressed *BnCSase* genes were selected for SNP variation analysis, as most *BnCSase* genes exhibited low expression levels. The analysis revealed that *BnCSase27* had the highest number of SNPs (373), including 296 intron variants. Additionally, *BnCSase1*, *BnCSase17*, and *BnCSase52* each contained one frameshift mutation; however, these mutations showed no significant correlation with germination shoot length under salt stress. Notably, *BnCSase48* was identified with two frameshift mutations (Fig. 11, Table S6). Further investigation revealed a frameshift mutation in *BnCSase48* located at position 69,862,389 bp on chromosome C04, caused by a T→TC deletion. This mutation was significantly associated with germination shoot length under salt stress, with a T-test p-value < 0.01 (Fig. 12A). The mutation significantly reduced the FPKM expression level of *BnCSase48* in seeds compared to the unmutated *BnCSase48*, with a T-test p-value < 0.01 (Fig. 12B).

### Detection of BnCSases expression under various abiotic stresses by qPCR technique

**Fig. 13:**
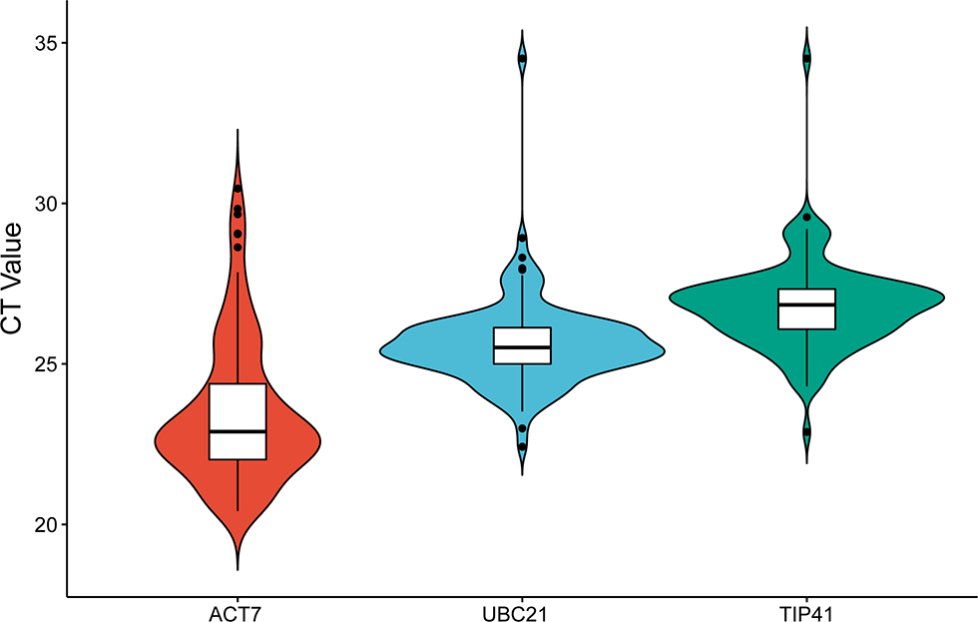
The CT value distribution of three candidate reference genes under abiotic stress

**Fig. 14:**
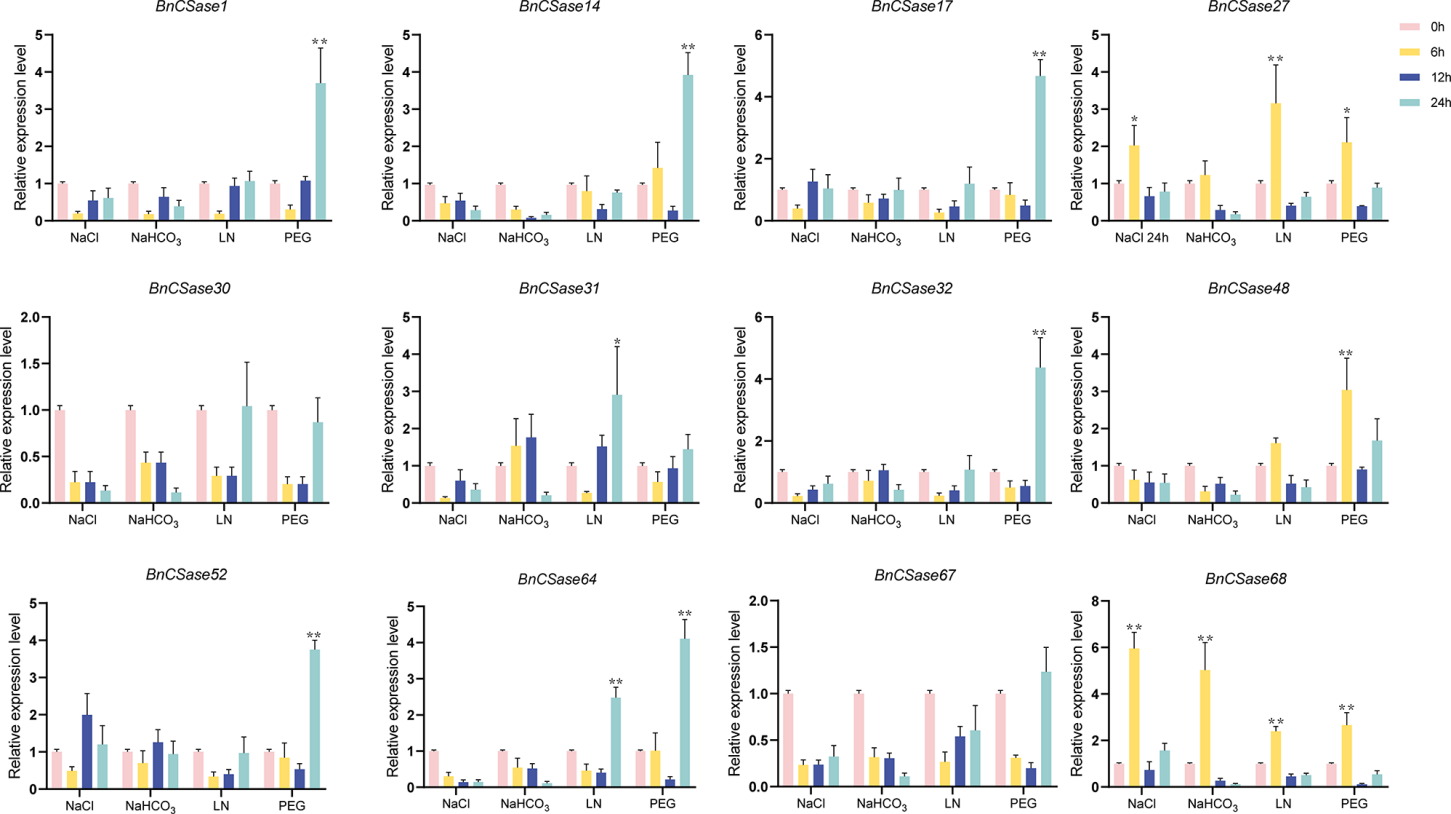
The relative expression of *BnCSase1*, *BnCSase14*, *BnCSase17*, *BnCSase27*, *BnCSase30*, *BnCSase31*, *BnCSase32*, *BnCSase48*, *BnCSase52*, *BnCSase64*, *BnCSase67*, *BnCSase68*, in leaves under 1.2% (w/v) NaCl, 0.2% (w/v) NaHCO_3_, Low Nitrogen (Table 2), and 20% (w/v) PEG 6000 after 0, 6 h, 12 h and 24 h. Data represent the mean ± standard error for threebiological experiments. Statistical differences between treatment groups were determined using one-way ANOVA. *: significant differences between treatments at *p* ≤ 0.05. **: significant differences between treatments at *p* ≤ 0.01

Recent studies have shown that *CSases* play a crucial role in plant responses to abiotic stress. To investigate the impact of *BnCSases* on the response of canola seeds to abiotic stress, we applied 1.2% (w/v) NaCl, 0.2% (w/v) NaHCO_3_, Low nitrogen (Table 2), and 20% (w/v) PEG 6000 at 6, 12, and 24 h. Three internal reference genes were used for expression validation (Fig. 13). ACT7 exhibited the highest CT values and expression levels across various abiotic stress conditions. Additionally, based on the assessment via NormFinder for the stability of three candidate reference genes, ACT7 demonstrated the strongest stability as a reference gene. Therefore, we selected ACT7 as the internal reference gene to analyze the relative expression of nine different bncsase genes (Fig. 14, Table S7). The results indicated that the expression of these genes significantly responded to different abiotic stresses. After NaCl treatment, the expression of *BnCSase1*, *BnCSase14*, *BnCSase17*, *BnCSase30*, *BnCSase31*, *BnCSase32*, *BnCSase48*, *BnCSase52*, *BnCSase64*, and *BnCSase67* showed no significant change at all time points, with the lowest expression at 6 h. The expression of *BnCSase27* and *BnCSase68* increased with the extension of treatment time, significantly increased at 6 h, reached a maximum, decreased at 12 h, and then increased again at 24 h. After NaHCO_3_ treatment, the expression of *BnCSase1*, *BnCSase14*, *BnCSase17*, *BnCSase27*, *BnCSase30*, *BnCSase31*, *BnCSase32*, *BnCSase48*, *BnCSase52*, *BnCSase64*, and *BnCSase67* did not show significant changes at all time points. The expression of *BnCSase68* first increased and then decreased, with a significant increase at 6 h, reaching a maximum, decreasing at 12 h, and then reaching the lowest expression at 24 h. After Low nitrogen treatment, the expression of *BnCSase1*, *BnCSase14*, *BnCSase17*, *BnCSase30*, *BnCSase32*, *BnCSase48*, *BnCSase5*2, and *BnCSase67* did not show significant changes at all time points. The expression of *BnCSase27* and BnCSase68 significantly increased at 6 h, reached a maximum, decreased at 12 h, and then increased again at 24 h. The expression of BnCSase31 and BnCSase64 showed different trends: *BnCSase31* increased at 12 h, while *BnCSase64* decreased at 12 h, and both significantly increased and reached their maximum expression at 24 h. After PEG 6000 treatment, the expression of *BnCSase30*, *BnCSase31*, and *BnCSase67* showed no significant changes at all time points. With the extension of treatment time, the expression of *BnCSase1* and *BnCSase32* decreased to the lowest at 6 h, increased at 12 h, and significantly increased at 24 h. The expression of *BnCSase17*, *BnCSase32*, *BnCSase52*, and *BnCSase64* decreased at 6 h, further decreased at 12 h, and then significantly increased and reached their maximum at 24 h. The expression of *BnCSase27*, *BnCSase48*, and *BnCSase68* significantly increased at 6 h, reached a maximum, decreased at 12 h, and then increased again at 24 h.

## Discussion

Gene family analysis facilitates the identification of key functional genes in crop genomes and provides a genetic foundation for the development of high-yield and high-quality germplasms. In this study, the CSase protein sequences of Arabidopsis thaliana were used as seed sequences, combined with Pfam domain search results. Ultimately, 69 members of the *B. napus CSase* gene family were identified, distributed across 19 defined chromosomes.

*Brassica napus* is an allotetraploid species with two sets of homologous chromosomes. It originated from the hybridization of *Brassica rapa* (AA: 2n = 20) and *Brassica oleracea* (CC: 2n = 18), forming an allotetraploid species (AACC: 2n = 38) [29]. Theoretically, the number of genes in *B. napus* should be the sum of those in *B. rapa* and *B. oleracea*. In this study, 32 BrCSase proteins and 36 BoCSase proteins were identified, while the number of *B. napus CSase* gene family members was slightly greater than the sum of the other two.

In the three subgroups (CysA, CysB, and CysC), the number of *B. napus CSase* genes matched the sum of those in *B. rapa* and *B. oleracea*. This similar distribution pattern of *CSase* genes across different species underscores their genetic conservation. However, within the CysD subfamily, *B. napus* harbors 14 CysD genes, a number notably higher than that of its diploid progenitor species, *B. rapa* and *B. oleracea* (5 genes each). This finding suggests that the CysD subfamily may have undergone gene expansion or duplication events during the speciation of *B. napus*. The increased copy number of CysD genes is likely associated with the expansion of the gene family, which is often correlated with enhanced adaptation to complex environmental stresses or developmental demands in plants. Moreover, the higher total number of *BnCSases* compared to the combined total of *BrCSases* and *BoCSases* further highlights the evolutionary distinctiveness of *BnCSases* (Fig. 2B; Table 1).

Phylogenetic analysis was conducted using the CSase protein sequences of *(A) thaliana* and *(B) napus*. Statistical analysis of the distribution of CSase proteins within the phylogenetic tree revealed that BnCSase proteins were evenly distributed across the four groups (CysA, CysB, CysC, and CysD). Theoretically, as a diploid, *(A) thaliana* should have half the number of genes found in *(B) napus*, a tetraploid species. However, the *CSase* family in *B. napus* exhibited a significantly higher number of members compared to other subgroups, emphasizing the evolutionary uniqueness of BnCSases.

Further analysis of gene structures and conserved domains suggests evolutionary conservation may exist among species, including wheat (*Triticum aestivum* L.) [28], foxtail millet (*Setaria italica* (L.) P. Beauvois) [30], alfalfa (*Medicago sativa* L.) [27]. Among all BnCSase proteins, Motif 2 and Motif 6 were identified as ubiquitous. This consistency indicates that these two motifs likely constitute characteristic sequences of BnCSase, playing pivotal roles in the structural and functional properties of these proteins. The promoters of *BnCSase* genes contain numerous light-responsive elements, suggesting their functions may be associated with photosynthesis. Cysteine, as a precursor of glutathione, can enhance the activities of superoxide dismutase (SOD) and catalase (CAT) enzymes [22], thereby improving the plant’s adaptability to oxidative stress. This process is closely related to the removal of reactive oxygen species (ROS) generated under light conditions. Therefore, light-responsive signals may regulate the expression of *CSase* genes to maintain the redox balance within cells. The photosynthetic process in plants is often accompanied by the accumulation of reactive oxygen species (ROS) under various stress conditions, such as salt stress and drought stress, leading to oxidative damage. Under salt stress, the electron transport chain in chloroplasts is disrupted, resulting in the overproduction of ROS, such as O₂⁻ and H₂O₂, which triggers membrane lipid peroxidation and chlorophyll degradation. Drought stress reduces stomatal conductance, causing excess light energy to be incompletely utilized by chlorophyll, thereby promoting ROS accumulation, including O₂⁻ and H₂O₂. The promoter regions of *BnCSase* are enriched with light-responsive elements, suggesting that *BnCSase* may mediate stress adaptation through light signaling and ROS regulation, thereby enhancing the stability of the photosynthetic system. Although the direct relationship between *CSase* activity and photosynthesis requires further experimental validation, it is evident that *CSases*, by enhancing the plant’s tolerance to oxidative stress, indirectly optimize the conditions necessary for efficient photosynthesis.

Ka/Ks analysis is a method used to measure selective pressure on genes, commonly applied to study the evolution of genes or gene families. In this context, Ka represents the nonsynonymous substitution rate (leading to amino acid changes), while Ks denotes the synonymous substitution rate (not resulting in amino acid changes) [31]. Ka/Ks analysis is a crucial tool for understanding gene evolution dynamics and uncovering key mechanisms behind adaptive evolution, making it suitable for discussion in scientific literature. Through genome collinearity analysis, we identified 139 collinear *CSase* gene pairs between *B. napus* and *B. rapa*, 147 collinear *CSase* gene pairs between *B. napus* and *B. oleracea* (Fig. 4), and 128 collinear *CSase* gene pairs within the *B. napus* genome (Fig. 5). All these genes exhibited Ka/Ks values less than 1 (Fig. 7, Table S5). indicating purifying selection, which suggests that their functions have been conserved during evolution. This implies that these genes may have already possessed important functions in *B. rapa* and *B. oleracea* and were further retained and refined in *B. napus*. Interestingly, the number of CysD genes in *B. napus* is greater than that in *B. rapa* and *B. oleracea*. Despite this increase in CysD gene number in *B. napus*, the presence of purifying selection indicates that these additional genes remain functionally constrained. This suggests that the CysD genes under purifying selection may play critical roles in the growth, development, or stress responses of *B. napus*. Notably, the number of collinear *CSase* gene pairs across species exceeds the number of *BnCSase* genes identified in this study, demonstrating that *CSase* genes are evolutionarily correlated and exhibit evolutionary conservation both within and across species.

miRNAs (microRNAs) are a class of small non-coding RNAs that regulate gene expression at the post-transcriptional level by inhibiting the translation of messenger RNAs (mRNAs) or promoting mRNA degradation [32]. miRNAs are approximately 20–24 nucleotides (nt) in length and are found in plants, animals, and certain viruses [33]. In plants, miRNAs primarily achieve gene silencing by mediating the cleavage of target RNAs or by repressing translation, thereby inhibiting gene expression [34]. They negatively regulate protein synthesis at the transcriptional or post-transcriptional levels, influencing plant growth, development, and responses to abiotic stress [35].

For example, in *B. napus*, bna-miR159, bna-miR6029, and bna-miR827 negatively regulate target genes related to the nitrogen metabolism pathway, thereby impacting nitrogen signaling and consequently affecting pod thickness [36]. Additionally, bna-miR156, bna-miR172, and bna-miR319 fine-tune the expression of key target genes involved in auxin-related pathways [37]. In transgenic lines overexpressing bna-miR319, abnormal development of serrated leaves and shoot apical meristems was observed. Fu et al. analyzed miRNA-mRNA expression in *B. napus* seedlings under treatment with 10 mg/L Cd²⁺ and reported that cadmium stress significantly altered the expression of 22 miRNAs belonging to 11 families in roots and 29 miRNAs belonging to 14 families in shoots. They identified eight miRNA-mRNA interaction pairs in roots and eight in shoots. Furthermore, four genes targeted by bn-miR398 were found to be involved in the detoxification pathway of superoxide radicals [38]. In this study, a total of 47 Brassica napus miRNAs were found to target 30 *BnCSase* genes through cleavage and translational repression (Fig. 9). It is hypothesized that miRNAs and *BnCSases* form a miRNA-target gene regulatory network, with B*nCSases* participating in the final step of cysteine biosynthesis. Interestingly, the number and distribution of cis-regulatory elements in *BnCSases* vary across different subgroups (Fig. 3), leading to distinct expression patterns (Fig. 9). For instance, *BnCSase10* and *BnCSase43* are both targeted by bna-miR161 and exhibit relatively consistent expression levels. They are almost undetectable in leaves under various abiotic stresses but show moderate expression in roots. However, their expression patterns differ from those of other *BnCSase* genes. The analysis of *BnCSase* expression patterns under abiotic stress conditions, as depicted in the heatmap (Fig. 10), revealed distinct tissue-specific expression profiles between leaves and roots. Significant variation in gene expression levels was observed for several BnCSase members, suggesting that these genes are regulated in an organ-specific manner. Notably, certain genes within the CysB family, such as *BnCSase14*, *BnCSase15*, *BnCSase46*, and *BnCSase48*, displayed pronounced upregulation in leaves. In contrast, specific members of the CysD family, including *BnCSase27*, *BnCSase28*, and *BnCSase69*, exhibited preferential expression in roots. These divergent expression patterns imply functional specialization among the *BnCSase* genes, with some likely contributing to oxidative stress mitigation and photosynthetic modulation in leaves, while others may facilitate nutrient acquisition and osmotic regulation in roots. These findings underscore the complex and tissue-specific regulatory mechanisms governing *BnCSase* gene expression in response to abiotic stress. Leaves are one of the primary organs for cysteine and glutathione synthesis. Recent studies have shown that *CSases* play a significant role in plant responses to abiotic stress. Previous research indicates that CSases enhance plant antioxidant capacity and mitigate the impact of environmental stress on photosynthesis by increasing cysteine and downstream glutathione levels [36, 38]. For example, under 4 h of high-temperature stress, members of the SCS subfamily, *TaCSase5*, *TaCSase7*, and *TaCSase9*, exhibit upregulated expression. Currently, research on the expression patterns of plant cysteine synthase genes under abiotic stress remains limited, especially for *B. napus*. Further exploration is needed to understand the role of *BnCSases* in abiotic stress responses. Therefore, elucidating the expression patterns of *BnCSase* genes during abiotic stress defense in *B. napus* is highly significant. Genetic variation analysis of the BnCSase gene family revealed a T→TC frameshift mutation inthe coding sequence of BnCSase48 (ChrC04: 69,862,389 bp)(Figure12). Notably, under salt stress conditions, the mutation significantly reduced the transcript levels (FPKM) of *BnCSase48* and the stem length of seedlings. A plausible explanation for this reduction is that the frameshift mutation introduced a premature termination codon (PTC), triggering nonsense-mediated mRNA decay (NMD), which in turn decreased mRNA abundance. Consistent with this notion, our results showed that the transcript levels of *BnCSase48* were indeed decreased in the mutant.

In this study, qPCR technology was used to measure the relative expression levels of *BnCSase1*, *BnCSase14*, *BnCSase17*, *BnCSase27*, *BnCSase30*, *BnCSase31*, *BnCSase32*, *BnCSase48*, *BnCSase52*, *BnCSase64*, *BnCSase67*, and *BnCSase68* in leaves at four time points: 0, 6, 12, and 24 h (Fig. 14). The findings revealed that under abiotic stress, *CSases* from different subfamilies exhibited similar expression trends, indicating that *BnCSase* genes have subfamily-specific expression patterns. For instance, *BnCSase17* and *BnCSase52* showed similar expression trends under different abiotic stresses at various time points. Interestingly, *BnCSase68* displayed a consistent expression trend across different abiotic stresses and time points. Its expression initially increased, then decreased, and either increased or decreased again. At 6 h, *BnCSase68* exhibited significantly high expression levels, suggesting its prominent role in responding to abiotic stress to mitigate potential damage to plants. Over time, as plants gradually adapted to the stress, the expression levels decreased. Moreover, certain genes (e.g., *BnCSase17*, *BnCSase32*), despite harboring an abundance of **cis**-acting elements within their promoter regions, exhibited relatively low expression levels under stress conditions. This apparent discrepancy may be attributed to post-transcriptional regulatory mechanisms. Although the promoter regions of these genes contain multiple stress-responsive **cis**-acting elements, such as ABREs, the activation of transcription likely requires the binding of specific transcription factors (TFs). Under certain stress conditions, the corresponding TFs may remain uninduced or inactive, thereby limiting transcriptional activation despite the presence of these **cis**-regulatory sequences. Furthermore, we observed that *BnCSase27* and *BnCSase68* exhibited similar expression patterns in response to four types of abiotic stresses. Since both *BnCSase27* and *BnCSase68* belong to the CysD subfamily, these findings suggest that certain members of the CysD subfamily are evolutionarily conserved, potentially indicating their critical roles in plant growth, development, and stress responses.

## Materials and methods

### Identification and chromosome mapping of BnCSase family members

The genomic data for ZS11 (*B. napus*), Chiifu (*B. rapa*), and HDEM (*B. oleracea*) were obtained from the BRAD database (http://brassicadb.cn/#/), including the whole-genome file, GFF3 file, CDS file, and protein sequence file for ZS11, as well as the whole-genome files, GFF3 files, and protein sequence files for Chiifu and HDEM [39]. Nine CSase protein sequences from *Arabidopsis thaliana* were downloaded from the TAIR database (https://www.arabidopsis.org/) and used as reference sequences. These sequences were employed to identify putative BnCSase proteins in the *B. napus* protein dataset through BLASTp analysis (e-value < 1e-10) [40]. The PF00291 conserved domain file was retrieved from the Pfam database and used with HMMER software (http://www.hmmer.org/) to search for potential BnCSase proteins in the *B. napus* protein sequences. Redundant sequences were removed, and high-confidence BnCSase candidates were identified [41, 42] The intersection of the results from BLASTp and HMMER analyses was taken to compile a final set of BnCSase protein candidates. These candidates were further validated using the NCBI CD-Search tool, resulting in the identification of 69 *BnCSase* gene family members. These genes were subsequently named *BnCSase1* to *BnCSase69* based on their chromosomal positions. The physical and chemical properties of the BnCSase proteins were predicted using the ExPASy website [43]. Subcellular localization predictions were performed using the Plant-mPLoc website [44]. The Gene Density Profile function of TBtools was used to extract the gene density of *B. napus* chromosomes. The chromosomal location data of the *BnCSase* genes were retrieved from the GFF3 files of *B. napus* and visualized using TBTools software [45].

### Phylogenetic analysis

The CSase protein sequences of *(A) thaliana* and the identified *(B) napus* were imported into MEGA 11 software for multiple sequence alignment using the ClustalW algorithm. A phylogenetic tree was constructed using the Neighbor-Joining (NJ) method with 1000 bootstrap replications. Similarly, the identified CSase protein sequences of *B. rapa* and *B. oleracea* were analyzed using the same method to construct their phylogenetic tree. Two sets of phylogenetic trees with bootstrap values were saved in NWK format. Finally, the phylogenetic trees were visualized and refined using the online platform Evolview 3.0 (https://www.evolgenius.info/evolview/) [46, 47].

### Prediction of gene structure, protein conserved motifs and promoter cis-acting elements

The full-length sequences of the initially identified BnCSase proteins were analyzed using the online tool MEME [48] (https://meme-suite.org/meme/tools/meme) to identify conserved sequences, key functional sites, and motifs, with the parameters set to identify 10 motifs. The promoter regions (2000 bp upstream) of the *BnCSase* genes were extracted using TBtools, and cis-acting elements were predicted with PlantCare [49]. Finally, the phylogenetic tree, motif analysis, and gene structure of the *BnCSase* genes were visualized using TBtools.

### Collinear analysis of csases gene

In TBtools, the Advanced Circos tool was used to perform intraspecific synteny analysis of the *BnCSase* genes in *B. napus*, while the MCScanX module was employed to analyze the interspecific synteny relationships between *B. napus*, *B. rapa*, and *B. oleracea* [50]. ParaAT and KaKs_Calculator were used together to calculate ka/ks [51, 52].

### Protein-protein interaction network of BnCSases

The protein sequence of BnCSase was initially submitted to the String database [53], and the protein-protein interaction (PPI) network data of BnCSase were visualized using the String database.

### Analysis of interaction between Microrna and BnCSases target genes

The CDS sequence of *BnCSase* was submitted to the psRNA Target website (https://www.zhaolab.org/psRNATarget/analysis?function=3/, accessed on September 25, 2024) [54]., and the database parameter for *B. napus* was selected to obtain the targeting relationship information between MicroRNA and *BnCSase*. The targeting relationship information was then visualized using the R software packages ‘ggplot2’ and ‘ggalluvial’.

### Transcriptome expression pattern analysis

To investigate the expression patterns of the BnCSase gene under abiotic stress conditions, the sequence of *BnCSase* was uploaded to the BnIR database [55]. Based on TPM (Transcripts Per Million) data, the expression levels of BnCSase under various stress treatments were retrieved, including control (CK, 12 h), salt stress (200 mmol·L⁻¹ NaCl for 12 h), drought (12 h air-drying exposure), freezing stress (-4 °C for 12 h followed by recovery at 25 °C), cold stress (4 °C for 12 h followed by recovery at 25 °C), heat stress (38 °C for 12 h followed by recovery at 25 °C), and osmotic stress (300 mmol·L⁻¹ mannitol for 12 h). The obtained TPM values were normalized using the formula log_10_(TPM + 1). The resulting matrix was then visualized using TBTools software.

### Analysis of CSase gene variations in *Brassica napus*

The variation information of the *CSase* gene in *B. napus* was obtained from the BnVIR database (http://yanglab.hzau.edu.cn/BnVIR) [56], and the correlation analysis was conducted using the T.test function.

### Plant materials and handling

The seeds of *B. napus* ZS11(provided by the research group of Professor Shen Jinxiong at the National Rapeseed Engineering Technology Research Center, Huazhong Agricultural University, China) were placed on wet gauze (soaked with water) and germinated in a plant growth chamber at 20 to 22 °C with 65% humidity under long-day conditions (16-hour light/8-hour dark cycle) [57]. After one week, the seedlings were transferred to the previously described hydroponic system [58] and cultured under the same conditions for an additional 14 days until the fourth leaves fully expanded (the plant samples were leaf tissues). In the stress treatment experiments, two-week-old hydroponically cultured ZS11 plants were subjected to stresses of 1.2% (w/v) NaCl, 0.2% (w/v) NaHCO_3_, low nitrogen (Table 2), and 20% (w/v) PEG 6000. Leaf samples were collected after 0, 6, 12, and 24 h of treatment. Seedlings without stress treatment were used as the control group. Each treatment and time point included three biological replicates. The collected leaf samples were immediately frozen in liquid nitrogen and used for the analysis of *BnCSase* gene expression levels.

### Relative expression of BnCSases under different abiotic stresses

Total RNA from the control and abiotic stress-treated ZS11 was extracted using the RNA isolater Total RNA Extraction Reagent (Vazyme, China), and cDNA was synthesized using the HiScript III RT SuperMix (Vazyme, China) kit. qPCR primers for the *BnCSase* gene were designed using Primer Premier 5 (Table 3), and *BnCSase* gene expression was analyzed using the AceQ Universal SYBR qPCR Master Mix (Vazyme, China). The relative expression levels of BnCSase genes were calculated using the 2^−ΔΔCt^ method. Each sample included three biological replicates, with each replicate containing three technical replicates. The CT values of ACT7, UBC21, and TIP41 at different time points under four abiotic stress treatments were calculated (Fig. 12). ACT7, as a reference gene, exhibited the highest expression levels under abiotic stress conditions. Furthermore, based on the evaluation of the stability of three candidate reference genes using NormFinder [59], ACT7 demonstrated the strongest stability as a reference gene (Table S7). Therefore, ACT7 was selected as the internal reference gene for subsequent analyses. Data are presented as the mean ± standard error of three biological experiments. Statistical differences between treatment groups were determined using one-way ANOVA. *: Significant differences between treatments at *p* ≤ 0.05. **: Significant differences between treatments at *p* ≤ 0.01.

**Table 3:** qPCR primer sequence of *BnCSase* genes

Gene Name	Forward Primer(5’→3’)	Reverse Primer(3’→5’)
*BnCsase1*	CCCTGCTCCAGCGTCAAA	CAGCCGCCGTGAATGCTA
*BnCsase14*	CCCGTTGACTTCTCGCCA	TGAGAGCAGCCGGTAAAGG
*BnCsase17*	CTGCGGCCGCTGCTATAA	TAAGTAACGTTCCCCGCCAC
*BnCsase27*	GGTTGCGTCGCACGTATTG	CGCCGGTTGCCTCAATCA
*BnCsase30*	GCATCAGCCAAAGCACGAG	CGCGTTTGGCCTGAGTTG
*BnCsase31*	TGGCTTGTGTTGGTGGGG	TCCGAATCCCGCAGCTTC
*BnCsase32*	CGGCTGCCAAGGGATACAA	GCGCCTTTCATGCCCTTG
*BnCsase48*	AAGGCGGCTGCTTTCACG	GAGCAGCATTCTCGGCGA
*BnCsase52*	ACGATCTCTGGAGCAGGGA	CCAGGTTGCCCTCCACTG
*BnCsase64*	GAGACGATCCCCCGCATC	TCGCGTTTGGCCTGAGTT
*BnCsase67*	GCCTCTGCCATCGTCTCG	CCGAACCGACCGAACGAA
*BnCsase68*	CACGTGTTGCCGCCAAG	ATTACCGCCGGTTGCCTC
*ACT7*	GCTGACCGTATGAGCAAAG	AAGATGGATGGACCCGAC
*UBC21*	CCTCTGCAGCCTCCTCAAGT	CATATCTCCCCTGTCTTGAAATGC
*TIP41*	TGAAGAGCAGATTGATTTGGCT	ACACTCCATTGTCAGCCAGTT

## Conclusions

A total of 69 *CSase* genes were identified in the rapeseed genome, distributed across 18 chromosomes. Comprehensive analyses were conducted, including phylogenetic relationships, physicochemical properties of proteins, subcellular localization, gene structures, promoter cis-acting elements, co-expression relationships, protein-protein interaction (PPI) networks, associated miRNAs, and SNP variations. Additionally, transcriptional expression patterns and responses to abiotic stress treatments were investigated. The results indicated that the expression patterns of *BnCSase* genes vary significantly under different abiotic stresses, suggesting that rapeseed exhibits stress-specific responses depending on the type of stress. This study provides valuable theoretical insights into the *BnCSase* gene family and its potential applications in improving stress resistance in rapeseed breeding.

## Electronic supplementary material

Below is the link to the electronic supplementary material.

Supplementary Material 1

Supplementary Material 2

Supplementary Material 3

Supplementary Material 4

Supplementary Material 5

Supplementary Material 6

Supplementary Material 7

Supplementary Material 8

Supplementary Material 9

## Data Availability

All the data generated or analyzed during this study are included in this published article and its supplementary information files.
