# Supplementary material for: Identification and expression analysis of the cysteine synthase (CSase) gene family in Brassica napus L. under abiotic stress
Source: BMC Plant Biol. 2025 Jun 5;25:770. doi: 10.1186/s12870-025-06532-8 (PMC12139137; doi:10.1186/s12870-025-06532-8)
Supplement: Supplementary file 1 — Supplementary Material 1 [file 12870_2025_6532_MOESM1_ESM.pdf]

>AtOAS-A1
MASRIAKDVTELIGNTPLVYLNNVAEGCVGRVAAKLEMMEPCSSVKDRIGFSMISDAEKKGLI
KPGESVLIEPTSGNTGVGLAFTAAAKGYKLIITMPASMSTERRIILLAFGVELVLTDPAKGMKG
AIAKAEEILAKTPNGYMLQQFENPANPKIHYETTGPEIWKGTGGKIDGFVSGIGTGGTITGAGK
YLKEQNANVKLYGVEPVESAILSGGKPGPHKIQGIGAGFIPSVLNVDLIDEVVQVSSDESIDMA
RQLALKEGLLVGISSGAAAAAAIKLAQRPENAGKLFVAIFPSFGERYLSTVLFDATRKEAEAM
TFEA
>AtOAS-A2
MIADAEAKGLIKPGESVLIEPTSGNTGVGLAFTAAAKGYKLVITMPASMSIERRIILLAFGAELI
LTDPAKGMKGAVAKAEEILAKTPNGYMLQQFENPANPKIHYETTGPEIWKGAGKYLKEQNTN
IKLYGVEPVESPILSGGKPGPHKIQGIGAGFIPGILDVDLIDEVVQVSSEESIDMARLLAREEGLL
VGISSGAAATAAIKLAKRPENAGKLIVAVFPSFGERYLSTVLFDAARKEAETMTFEP
>AtOAS-B
MAATSSSAFLLNPLTSRHRPFKYSPELSSLSLSSRKAAAFDVSSAAFTLKRQSRSDVVCKAVSI
KPEAGVEGLNIADNAAQLIGKTPMVYLNNVVKGCVASVAAKLEIMEPCCSVKDRIGYSMITD
AEEKGLITPGKSVLVESTSGNTGIGLAFIAASKGYKLILTMPASMSLERRVLLRAFGAELVLTEP
AKGMTGAIQKAEEILKKTPNSYMLQQFDNPANPKIHYETTGPEIWEDTRGKIDILVAGIGTGGT
ITGVGRFIKERKPELKVIGVEPTESAILSGGKPGPHKIQGIGAGFVPKNLDLAIVDEYIAISSEEAI
ETSKQLALQEGLLVGISSGAAAAAAIQVAKRPENAGKLIAVVFPSFGERYLSTQLFQSIREECE
QMQPEL
>AtOAS-C
MVAMIMASRFNREAKLASQILSTLLGNRSCYTSMAATSSSALLLNPLTSSSSSSTLRRFRCSPEI
SSLSFSSASDFSLAMKRQSRSFADGSERDPSVVCEAVKRETGPDGLNIADNVSQLIGKTPMVY
LNSIAKGCVANIAAKLEIMEPCCSVKDRIGYSMVTDAEQKGFISPGKSVLVEPTSGNTGIGLAFI
AASRGYRLILTMPASMSMERRVLLKAFGAELVLTDPAKGMTGAVQKAEEILKNTPDAYMLQQ
FDNPANPKIHYETTGPEIWDDTKGKVDIFVAGIGTGGTITGVGRFIKEKNPKTQVIGVEPTESDI
LSGGKPGPHKIQGIGAGFIPKNLDQKIMDEVIAISSEEAIETAKQLALKEGLMVGISSGAAAAA
AIKVAKRPENAGKLIAVVFPSFGERYLSTPLFQSIREEVEKMQPERVSG
>AtCYS-D1
MEEDRCSIKDDATQLIGNTPMVYLNNIVDGCVARIAAKLEMMEPCSSVKERIAYGMIKDAED
KGLITPGKSTLIEATSGNTGIGLAFIGAAKGYKVVLTMPSSMSLERKIILLALGAEVHLTDPSKG
VQGIIDKAEEICSKNPDSIMLEQFKNPSNPQTHYRTTGPEIWRDSAGEVDILVAGVGTGGTLSG
SGRFLKEKNKDFKVYGVEPTESAVISGGKPGTHLIQGIGAGLIPDNLDFNVLDEVIQVTSVEAI
ETAKLLALKEGLLVGISSGAAAAAAIKVAKRPENAGKLIVVIFPSGGERYLSTSLFESVRHEAE
NLPIQ
>AtCYS-D2
MEDRCLIKNDITELIGNTPMVYLNNVVDGCVARIAAKLEMMEPCSSVKDRIAYSMIKDAEDK
GLITPGKSTLIEPTAGNTGIGLACMGAARGYKVILVMPSTMSLERRIILRALGAELHLSDQRIGL
KGMLEKTEAILSKTPGGYIPQQFENPANPEIHYRTTGPEIWRDSAGKVDILVAGVGTGGTATGV
GKFLKEQNKDIKVCVVEPVESPVLSGGQPGPHLIQGIGSGIVPFNLDLTIVDEIIQVAGEEAIETA
KLLALKEGLLVGISSGAAAAAALKVAKRPENAGKLIVVVFPSGGERYLSTKLFDSIRYEAENLP
IE
>AtDES1
MEDRVLIKNDVTELIGNTPMVYLNKIVDGCVARIAAKLEMMEPCSSIKDRIAYSMIKDAEDKG
LITPGKSTLIEATGGNTGIGLASIGASRGYKVILLMPSTMSLERRIILRALGAEVHLTDISIGIKGQ
LEKAKEILSKTPGGYIPHQFINPENPEIHYRTTGPEIWRDSAGKVDILVAGVGTGGTVTGTGKF
LKEKNKDIKVCVVEPSESAVLSGGKPGPHLIQGIGSGEIPANLDLSIVDEIIQVTGEEAIETTKLL
AIKEGLLVGISSGASAAAALKVAKRPENVGKLIVVIFPSGGERYLSTELFESVRYEAENLPVE
>AtCYS-C1
MASVSRRLLRRETIPCFSHTVRKLFSTVGSPSFAQRLRDLPKDFPSTNAKRDASLLIGKTPLVFL
NKVTEGCEAYVAAKQEHFQPTCSIKDRPAIAMIADAEKKKLIIPGKTTLIEPTSGNMGISLAFM
AAMKGYRIIMTMPSYTSLERRVTMRSFGAELVLTDPAKGMGGTVKKAYDLLDSTPDAFMCQ
QFANPANTQIHFDTTGPEIWEDTLGNVDIFVMGIGSGGTVSGVGRYLKSKNPNVKIYGVEPAE

SNILNGGKPGPHAITGNGVGFKPEILDMDVMESVLEVSSEDAIKMARELALKEGLMVGISSGA
NTVAAIRLAKMPENKGKLIVTIHASFGERYLSSVLFDELRKEAEEMKPVSVD
>AtSCS
MDLKSNNHQQQQVLDGSDIVELVENEKVFDKFVEQKFQQLDQDEDGKLSVTELQPAVADIG
AALGLPAQGTSPDSDHIYSEVLNEFTHGSQEKVSKTEFKEVLSDILLGMAAGLKRDPIVILRM
DGEDLSEFVHGPGYEIESISVFSELSSSKDASLRDCIVKALQSLSVDHGMPPSNDPWVMSNIVE
PIVDSCLDEEDKREKSASQERFLEAFKRVVESVAQRLNEQPVIVAHSENTFDGSGIRRLLSNKF
EFDKALNVAMETIPKDRQGKVSKGYLRAVLDTVAPSATLPPIGAVSQMDNMIMEALKMVNG
DDGNVVKEEEFKKTMAEILGSIMLQLEGSPISVSSNSVVHEPLTSATFLPSTSTDTEEPSN
>BnCSase1
MIDCVYFGQLIGNTPLVYLNSVAEGCVGRVAAKLEMMEPCSSVKDRIGFSMISDAEKKGL
IKPGESVLIEPTSGNTGVGLAFTAAAKGYKLIITMPASMSVERRIILLAFGVELVLTDPA
KGMKGAIAKAEEILAKTPNGYMLQQFENPANPKVTASYFTSAYVNMFLSVIELLTVQFQI
HYETTGPEIWKGTDGKIDGFISGIGTGGTITGAGKYLKEQNPNVKLYGVEPIESAILSGG
KPGPHKIQGIGAGFIPSVLEVNLIDEVVQVSSDESIDMARLLARKEGLLVGISSGAAAAA
AIKLAKRPENAGKLFVAVFPSFGERYLSTVLFDATRKEAESMTFEA
>BnCSase2
MDSVRLPTAPSSLRSQMLGQPLHRIPLPPCNGRSNLRFKPVIGTNLSRENHVSPVAVITR
DETSVAPLASPQPRLKVSPSSLQYPAGYLGAVPERASDPENGSIAEAMEYLTNILSTKVY
DVAIESPLHLAKKLSERLGVRLFLKREDLQPVFSFKLRGAYNMMVKLPAEQLAKGVICSS
AGNHAQGVALSAAKLGCTAVIVMPRTTPEIKWQSVENLGATVVLVGDSYDEAQAFAKQRA
EEEGLTFIPPFDHPDVIAGQGTVGMEITRQAKGPLHAIFVPIGGGGLIAGIASYVKRVCP
EVKIIGVEPADANTMALSLHHGERVILDQVGGFADGVAVKVVGEETFRISRKLVDGVVLV
TRDAICASIKDMFEEQRNILEPAGALAIAGAEAYCKYYGLKDVNVVAITSGANMNFDKLR
IVTELANVGRQQEAVLATILPEKPGSFKKFCELVGAMNITEFKYRCGSEKESVVLYSVGV
HTPGELKELEKRMESSQLRTRNLTSSDLVKDHLRYLMGGRSSVEEEVLCQFTFPERPGAL
MNFLDSFSPRWNISLFHYRAEGGAGANVLVGIQVPEQEMVEFRNRAQVLGYEYVLVSEDT
VFKLLMH
>BnCSase3
MALSSPSLLRLLPHHPFTLTTSKRHRFLSLNHEPSSSSLVVAAVSSKPSTRTKQKSKSKS
KPPPPPPPPVTTVSHEVGTEDSETVNIAEDVTQLIGSTPMVYLNRVTDGCVADVAAKLES
MEPCRSVKDRIGLSMINEAEDRGDITPRKSVLVEPTTGNTGLGIAFVAAAKGYKLIVTMP
ASINVERRMLLRALGAEIVLTSPEKGLKGAVEKAKEIVLKTKNAYMFQQFDNTANTKIHF
ETTGPEIWEDTMGNVDIFVAGIGTGGTVTGTGSFLKMMNPDIKVVGVEPSERSVISGDSP
GYVPGILDVKLLDEVFKVSNEEAIEMARRLALEEGLLVGISSGAAAVAAISLAKRAENTG
KLITVLFPSHGERYITTALFSSINKEVQEMSH
>BnCSase4
MTSSSLFNASLSSLSPNQDLFRRHTSSPLLRYRPVLVSCTSPADGNNTTTPIETITKPRR
TENTIRDDARLQRSTAANPFSARYVPFNAPPGSSEHYSLDEIVYRSRSGGLLDVEHDMEA
LKRFDGAYWRDLFDSRVGKSTWPYGSGVWSKKEWVLPEIDDDDIVSAFEGNSNLFWAERF
GKKFLGMNDLWVKHCGISHTGSFKDLGMTVLVSQVNRLRKMKRPVVGVGCASTGDTSAAL
SAYCASAGIPSIVFLPANKISMAQLVQPIANGAFVLSIDTDFDGCMKLIREITAELPIYL
ANSLNSLRLEGQKTAAIEILQQFDWQVPDWVIVPGGNLGNIYAFYKGFKMCQELGLVDRI
PRMVCAQAANANPLYLHYKSGWKDFKPMTASTTFASAIQIGDPVSIDRAVYALKKCDGIV
EEATEEELMDAMAQADSTGMFICPHTGVALTALFKLRNRGVIAPTDRTVVVSTAHGLKFT
QSKIDYHSKAIPDMACRFSNPPVEVKADFGAVMDVLKSYLGSQTLRS
>BnCSase5
MASFSLLHSATYFPSHSKTSFKPHSTASSTVRCTSQTTPSPAAPPQKHRRSDENIRDEAR
RRPQLQNLSARYVPFDAPPLSTESYSLDEIVYRSQSGGLLDVQHDFAALKRYDGAFWRNL
FDSRVGKTTWPYGSGVWSKKEWVLPEIDDDDIISAFEGNSNLFWAERFGKQYLQMNDLWV
KHCGISHTGSFKDLGMTVLVSQVNRLRKMNKPVVGVGCASTGDTSAALSAYCAAAGIPSI
VFLPADKISTAQLVQPIANGAFVLSLDTDFDGCMHLIREVTAELPIYLANSLNSLRLEGQ
KTAAIEILQQFNWEVPDWVIVPGGNLGNIYAFYKGFHMCKELGLVDRIPRLVCAQAANAN

PLYLHYKSGFDQDFNPMKADTTFASAIQIGDPVSIDRAVYALKKSNGIVEEATEEELMDA
TALADSTGMFICPHTGVALTALMKLRESGVIEANDRTVVVSTAHGLKFTQSKIEYHSKNI
QEMACRLANPPVKVKAEFGSVMDVLKEYLKNNESKNVD
>BnCSase6
MEAKKRHEYAADLSSIKKAHERIKPYIHKTPVLTSESLNSISGRSLFFKCECFQKGGAFK
FRGACNAVLSLDAEQAAKGVVTHSSGNHAAALSLAAKMQGIPAYIVVPKGAPKCKVDNVI
RYGGKVIWSEATMSSREEVASRVLHETGSVLIHPYNDGRIISGQGTVALELLEQIQEIDT
IIVPISGGGLISGVALAAKSIKPSIRIIAAEPKGADDAAQSKVAGRIITLPVANTIADGL
RASLGDLTWPVVRDMVDGVVVVEDGEIIEAMRMCYEMLKVSVEPSGAIGLAAVLSTSFRS
NPCWKDCKNIGIVLPGGNVDLGVLWDSLKSSE
>BnCSase7
MEDRCLIKNDVTELIGNTPMVYLNKVVDGCLARIAAKLEMMEPCSSVKDRIAYSMIKDAE
DKGLITPGKSTLIEPTAGNTGIGLACIGAARGYKVILLMPSTMSLERRIILKALGAELHL
TDVKIGIQGMLEKTEEILSKTPGGFVPQQFENPANPEIHYRTTGPEIWRDSAGKVDILVA
GVGTGGTISGVGKFLKEMNKDIKVCAVEPAESPVLSGGERGPHLIQGIGSGIIPTNLELS
IVDEIIQVKGEEAIETAKLLARKEGLLVGISSGAAAAAALKVAKRPENAGKLIVVVFPSG
GERYLSTKLFDSVRFEAENLPIE
>BnCSase8
MMVKLTSEQLAKGVICSSAGNHAQGVAMSAAKLGCTAVIVMPRTTPEIKWQSVEDLGATV
VLVGDSYDEAQAFAKQRAEEEGLSFIPPFDHPDVIAGQGTVGMEITRQAKGPLHAIFVPI
GGGGLIAGIASYVKRVCPEVKIIGVEPADANTMALSLHHGERVILDQVGGFADGVAVKEV
GKETFRICQNLVDGVVLVTRDAICASIKDMFEEQRNILEPAGALALAGAEAYCKYYGLKD
VNVVAITSGANMNFDKLRIVTELANVGRQQEAVLATLLPEKPGSFKQFCELIGPMNITEF
KYRCGSEKESVVLYSVGVHTAGELKALEKRMESSQLRTRNLTTSDLVKDHLRYLMGGRSS
VEEEVLCQFTFPERPGALMNFLDSFSPRWNISLFHYRAEGGAGANVLVGIQVSEQEMEEF
RNRAQVLGYEYVLVSEDAIFNLLMH
>BnCSase9
MATSGTASTFRPSISASSRLTHLRSPPFKVPNFTPLPSSRSRSFSVSCTIAKDPTFLMAE
AEKNKAAGSDPTLWKRPDSFGRFGKFGGKYVPETLMHALSELETAFYSLATDDDFQRELA
GILKDYVGRESPLYFAERLTEHYRRENGEGPLIYLKREDLNHTGAHKINNAVAQALLAKR
LGKKRIIAETGAGQHGVATATVCARFGLQCIIYMGAQDMERQALNVFRMRLLGAEVRGVH
SGTATLKDATSEAIRDWVTNVETTHYILGSVAGPHPYPMMVRDFHAVIGKETRRQAMEKW
GGKPDVLVACVGGGSNAMGLFHEFVDDTEVRMIGVEAAGFGLDSGKHAATLTKGDVGVLH
GAMSYLLQDDDGQIIEPHSISAGLDYPGVGPEHSFLKDMGRAEYYSVTDEEALEAFKRVS
RLEGIIPALETSHALAHLEKLCPTLPDGARVVLNFSGRGDKDVQTAIKYLEV
>BnCSase10
MATVWRRLLKTETIPRISQSTRKLFSTDAPSSFADRLRNLPKDFPSTQAKRDASLLIGRT
PLVFLNRVTEGCEAYIAAKQEHFQPTCSVKDRPALAMVADAEKKNLITPGKTTLIEPTSG
NMGISMAFIAALKGYKIIMTMPSYTSLERRVTMRSFGAELVLTDPAKGMGGTVKKAYDLL
ESTPDAHMLQQFANPANTQIHFDTTGPEIWEDTLGNVDIFVMGIGSGGTVSGVGQYLKSK
NPNVKIYGVEPAESNILNGGKPGPHAITGNGVGFKPDILDMDVMESVLEVSSEDAIKMAR
ELALKEGLMVGISSGANTVAAIRLAKMPENKGKLIVTIHASFGERYLSSVLFDELRKEAE
AMKPVSVD
>BnCSase11
MAAQLLLPPNPFTKSASAKLFFTGDDSTLKRKSKHPTRVSNGFSLRANAALRSNHSSSVE
IPSRWYNIVADLSVKPPPPLHPKTYEPIKPEDLAHLFPNEIIKQEATLERFIDIPEEVLE
IYKLWRPTPLIRAKRLEKLLQTPARIYFKYEGGSPAGSHKPNSAVPQAYYNAKEGVKNLV
TETGAGQWGSSLAFASSLFGLNCEVWQVANSYHQKPYRRLMMQTWGAKVHPSPSDLTEAG
RRILQVDPSSPGSLGIAISEAVEVAARNEDTKYCLGSVLNHVLLHQTVIGEECIKQMEAF
GETPDVIIGCTGGGSNFAGLSFPYIREKLKGKINPVIRAVEPSACPSLTKGVYAYDFGDT
AGLTPLMKMHTLGHDFIPDPIHSGGLRYHGMAPLISHVYEQGFMEAISIPQTECFQGRAI
QFARTEGIIPAPEPTHAIAATIREALRCKETGEAKVILMAMCGHGHFDLSSYDKYLRGEL
IDLSFSEERIRESLSKVPHVV

>BnCSase12
MASQLLLPTNKFTNSSLDKVFVTGDDLTLKRKPNHATRVSYGFSLRANAALISSHRSSVE
VPRQWYNLVADLSVKPPPQLHPKTFEPIKPDDLTHLFPNEIIKQEETLERFIDIPEEVLE
IYKLWRPTPLIRAKRLEKLLQTPARIYFKYEGGSPAGSHKPNSAVPQAYYNAKEGVKNVV
TETGAGQWGSSLAFASSLFGLDCEVFQVAHTYQQKPYRRLMMQTWGAKVHRSPSELTEAG
RRILQADPSSTGSLGIAISEAVEVASRNEDTKYCLGSVFNHVLLHQTVIGEECIKQMEEY
GETPDVIIGCTGGGSNFAGLSFPYIREKLKGKINPIIRAVEPSACPSLTKGVYAYDFGDT
AGLTPLMKMHTLGHDFIPSPIHSGGLRYHGMAPLVSHIYDQGFMEAISIPQTECFQGAIQ
FARTEGIIPAPEPTHAIAATIREALRCKETGEAKVILMAMCGHGHFDLASYEKYLRGELV
DLSFSEEKIQESLSKVPLVV
>BnCSase13
MAMLMASRFNSEAKIASRFLSTLFRNQRVTASSSSSSSSLLLNPLTSSTLRHFRSSPEIS
SLSFSASGFPLAMKSQQQSRSYGDVSKRDPCEAVKRENGADGLNIAENVSQLIGKTPMVY
LNSMAKGCVANIAAKLEIMEPCCSVKDRIGYSMVTDAEQKGFISPGKSVLVEPTSGNTGI
GLAFIAASRGYRLILTMPSSMSMERRVLLKAFGAELVLTDPAKGMTGAVQKAEEILKSTP
DAYMLQQFDNPANPKIHYETTGPEIWEDTKGKVDIFVAGIGTGGTITGVGRFIKEKNPKV
QVIGVEPTESDILSGGKPGPHKIQGIGAGFIPKNLDRAIMDEVIAISSEEAIETAKQLAL
KEGLMVGISSGAAAAAAIMVAKRPENAGKLIAVVFPSFGERYLSTPLFQSIREEVEKMQP
EI
>BnCSase14
MAAATSSSAFLLNPLTSRHRPFNYSPQLASLSLSSTKAATFCPLPAALKSKSQRCEDVVC
KAVSAKVEAGVESLNIAENAAQLIGKTPMVYLNNIVKGCVASVAAKLEIMEPCCSVKDRI
GYSMITDAEEKGLITPGKSVLVESTSGNTGIGLAFIAASKGYKLILTMPASMSLERRVLL
RAFGAELVLTEPAKGMTGAIQKAEEILKNTPNSYMLQQFDNPANPKIHYETTGPEIWEDT
RGKVDILVAGIGTGGTITGVGRFIKERKPELKVIGVEPTESAILAGGKPGPHKIQGIGAG
FIPKNLDQTVVDEYISISSDEAIETAKQLALQEGLLVGISSGAAAAAAIQVAKRPENAGK
LIAVVFPSFGERYLSTMLFQSIREECEKMQPEI
>BnCSase15
MAAAISSSAFLLNPLTSRHRPFKYSPELSSLSLSSRKAATFDVLPASLSLKRQSQRCSSG
VVCKAVSVKPEAGIEGLNIAENAAQLIGKTPMVYLNNIVKGCVASVAAKLEIMEPCCSVK
DRIGYSMITDAEEKGLITPGKSVLVESTSGNTGIGLAFIAASKGYKLILTMPASMSLERR
VLLRAFGAELVLTEPAKGMTGAIQKAEEILKNTPDSYMLQQFDNPANPKIHYETTGPEIW
EDTRGKVDILVAGIGTGGTITGVGRYIKERKPELKVIGVEPTESAILSGGKPGPHKIQGI
GAGFIPKNLDQTVVDEYIAISSEEAIEFAKQLALQEGLLVGISSGAAAAAAIQVAKRPEN
AGKLIAVVFPSFGERYLSTLLFQSIRDECENMQPEL
>BnCSase16
MEYLTSILSTKVYDVAIETPLHLAKKLSERLGVSMFLKREDLQPVFSFKIRGAYNMMAKL
PSEQLAKGVICSSAGNHAQGVAMSAAKLGCTAVIVMPRTTPEIKWQSVEDLGATVVLVGD
TYDEAQAFAKQRAEEEGLTFIPPFDHPDVIAGQGTVGMEITRQAKGPLHAIFVPSGWWFD
SWYCCYVKRVSPEVKIIGVEPADANSMALSLHHGERVILNQIGGFADGVAVKEVGEETFR
ICRKLMDGVVLVTRDAMCASIKDMFEEKRNILEPAGALAIAGAEAYCKYYGLKDVNVVP
>BnCSase17
MRYNTQSLLYFIDGSTIALTLSTTINTSLSISISFVDQSLLSPAWNYKDLYSMEYSIKDD
VTQLIGNTPMVYLNNIVDGCVARIAAKLEMMQPCSSVKDRIAYGMIKDAEDKGLISPGKN
ILIEPTSGNTGIGIAMVGAARGYKVVITMPASVSIERRIVLLALGAELHLTDPSKGVIGV
IVKAEEILSKTPDGFMLEQFRNPSNPQSHYETTGPEIWRDSAEKVDMLVVGVGTGGTISG
AGKFLKEKNKDFKVYGVEPAESAVLKLTTLVLEHSTLIFKQKKIINIRIKVNIRRQPGPH
GIQGIGAGLIPDNLDFSVLDEVIQVTSVEAIETAKLLALKEGLLVGISSGAAAAAAIKVA
KRPENAGKLIVVVFPSGGERYLSTPMFDSIRCEAENLAIE
>BnCSase18
MDSVRLPTAPSSLRSQTLGHLLPHRLRHIPLPPCTSKPLIAMITRSRNRVSPIAVLSGNE
TSISPPDSPPPRLKVNPSSLQYPAGYLGAVPDRASDPENGSITEAMEYLTSILSTKVYDV
AIETPLHLAKKLSERLGVSMFLKREDLQPVFSFKIRGAYNMMAKLPSEQLAKGVICSSAG

NHAQGVAMSAAKLGCTAVIVMPRTTPEIKWQSVEDLGATVVLVGDTYDEAQAFAKQRAEE
EGLTFIPPFDHPDVIAGQGTVGMEITRQAKGPLHAIFVPIGGGGLIAGIAAYVKRVSPEV
KIIGVEPADANSMALSLHHGERVILNQIGGFADGVAVKEVGEETFRICRKLMDGVVLVTR
DAMCASIKDMFEEKRNILEPAGALAIAGAEAYCKYYGLKDVNVVAITSGANMNFDKLRIV
TELANVGRQQEAVLATILPEKPGSFKQFCELVGPMNITEFKYRCGSRKEAVVLYSVGVHT
PGELKALEKRMESSQLKTTNLTTSDLVKDHLRYLMGGRSSVENEVLCRFIFPERPGALMK
FLDSFSPRWNISLFHYRAEGAAGANVLVGIQVPENEIEEFRNRAQVLGYEYVLVSEDINF
KLLMQ
>BnCSase19
MAPVKITGAVVAAAATMVMLSYCFFRFSSDKLDSCSSSSSSKKKKKLSTRNGLVDAIGNT
PLIRINSLSDATGSEILGKCEFLNPGGSVKDRVAVKIIEEALESGLLFPGGIVTEGSAGS
TAISLATVAPAYGCQCHVVIPDDAAIEKSQIIEALGATVERVRPVSITHKDHFVNIARRR
ADQANDLASSSKTRLASRINVAHQEKTNGCTAEEQKVPSLFSESVTGGFFADQFENLANY
RAHYEGTGPEIWQQTHGNIDAFVAAAGTGGTLAGVSRFLQEKNEKVKCFLIDPPGSGLFN
KVTRGVMYTREEAEGRRLKNPFDTITEGIGINRLTQNFLMAKIDGGFRGTDKEAVEMSRF
LLKKDGLFVGSSSAMNCVGAVRVAQALGPGHTIVTILCDSGMRHLSKFHDPQYLALYGLT
PTAVGLEFLGIK
>BnCSase20
MSATATPMADFLTKSPYTPPPWASHLRPLPSHTFSLAHRPTPIHRWNLPNLPNGTELWIK
RDDFTGMELSGNKVRKLEFLMADAVEQQADTVITIGGIQSNHCRATTLLADGDPGLVGNL
LVERLVGANVHLISKEEYSSIGSEALTSALKEKLEKEGKKPYVIPVGGSNSLGTWGYIEA
AREIEEQLKCRTDGLKFDDIVVACGSGGTIAGISLGSWLGDLKAKVHAFSVCDDPDYFYD
FVQGLLDGLEAGVNARDIVSIHNAKGKGYAMNTSEELKFLKDLASATSVILDPVYSGKAA
YGLINEMTKDPKSWEGKKILFIHTGGLLGLYDKVDQMASLMGNWSRMDVQESVPRKEGVG
KMF
>BnCSase21
MEDRCLIKNDVTELIGNTPMVYLNKIADGCVARIAAKLEMMEPCSSIKDRIAYSMIKDAE
DKGLITPGESTLIEPTAGNTGIGLACIGAARGYKVIILMSSSMSLERRIILRALGAELLL
TDRSIGFKGMLEKTEEMLSKTPGGFVPQQFENPSNPEIHYRTTGPEIWRDSAGTIDILVA
GVGTGGTVTGVGKFLKEMNQNIKVCAVEPTESPVLSGGEPGPHLIQGIGAGIIPTNLDLT
IVDEIIQVIFASGGERYLSTKLFDSVRYEAENLPIE
>BnCSase22
MTSQLLLPPNPFTRPVSAKVFLTGDDLTLKRKSNQATRVSNGFSLRAKAALRSNHSSSVE
IPNQWYNLIADLSVKPPPPLHPKTFEPIKPEDLSHLFPNELIKQEATLERFIDIPEEVLE
IYKLWRPTPLIRAKRLEKLLQTPARIYFKYEGGSPAGSHKPNTAVPQAYYNAKEGVKNVV
TETGAGQWGSSLAFASSLFGLDCEVWQVANSYHQKPYRRLMMETWGAKVHPSPSDLTEAG
RKILESDPSSPGSLGIAISEAVEVAARNEDTKYCLGSVLNHVLLHQTVIGEECIKQMEDF
GETPDVIIGCTGGGSNFAGLSFPFIREKLKGNISPVIRTVEPSACPSLTKGVYAYDFGDT
AGLTPLMKMHTLGHDFIPDPIHAGGLRYHGMAPLISHVYEQGFMEAISIPQIECFQGAIQ
FARAEGIIPAPEPTHAIAATIREALRCKETGEAKVILMAMCGHGHFDLSSYDKYLKGELV
DLSFSEDKIRESLSKVPHVV
>BnCSase23
MTTTAAIHGTGEEAIETVKLLALKEGLLVGISTGAAAAAALKVAKRQENGGKLIALLRQP
ERAKTAFQDIEQVVEWAKNDSSMEEKQSIIQLVTNLEQAQQKTLPCKLDGKKTSNYSVTF
S
>BnCSase24
MASRIAKDVTELIGNTPLVYLNNVAEGCVGRVAAKLEMMEPCSSVKDRIGFSMISDAEEK
GLIKPGESVLIEPTSGNTGVGLAFTAAAKGYKLIITMPASMSVERRIILLAFGVELVLTD
PAKGMKGAIAKAEEILAKTPNGYMLQQFENPANPKIHYETTGPEIWKGTDGKIDGFVSGI
GTGGTITGAGKYLKEQNPNVKLYGVEPIESAILSGGKPGPHKIQGIGAGFIPSVLEVDLI
DEVVQVSSDESIDMARLLALKEGLLVGISSGAAAAAAIKLAKRPENAGKLFVAVFPSFGE
RYLSTVLFDATRKEAESMTFQA
>BnCSase25

MSSSSLFNASLSPLNPNQHPIRRHPSPSLLRHRPVAVSCASDGNTTTPIETSVKPRRNEN
TIRDDARLHRSTAVNPFSARYVPFNAPPNSSEHYSLDEIVYRSRSGGLLDVEHDMDALKH
FDGAYWRDLFDSRVGKSTWPYGSGVWSKKEWVLPEIDDDDIVSAFEGNSNLFWAERFGKQ
FLEMNDLWVKHCGISHTGSFKDLGMTVLVSQVNRLRKMNRPVVGVGCASTGDTSAALSAY
CAAAGIPSIVFLPANKISMAQLVQPIANGAFVLSIDTDFDGCMKLIREVTSELPIYLANS
LNSLRLEGQKTAAIEILQQFDWQAPEWVIVPGGNLGNIYAFYKGFKMCQELGLVDRIPRL
VCAQAANANPLYLHYKSGWKDFKPMTASTTFASAIQIGDPVSIDRAVYALKQCDGIVEEA
TEEELMDAMAQADSTGMFICPHTGVALTALFKLRKQGVIAPTDRTVVVSTAHGLKFTQSK
VDYHSKAIPDMACRFSNPPVEVKADFGAVMDVLKSYLGSQKLRS
>BnCSase26
MTSCSFSSSSLFSFNLPTQNSLHRRPPTLPRHAILSSTDGSSNGASSSSSSSPTVKPTRT
EDNIRDEARRHRSTSANPFSARYVPFNAPPGSTESYSLDDIVYRSESGGLLDVQHDLDAL
RAHDGAYWRNLFDSRVGKTKWPYGSGVWSKKEWVLPEIDHDDIVSAFEGNSNLFWAERFG
KTFLGMNDLWVKHCGISHTGSFKDLGMTVLVSQVNRLRKMNKPVVGVGCASTGDTSAALS
AYCASAGIPSIVILPANKISMAQLVQPIANGAFVLSIDTDFDGCMKLIREITSELPIYLA
NSLNSLRLEGQKTAAIEILQQFNWQVPDWVIVPGGNLGNIYAFYKGFKMCQELGLVDRIP
RLVCAQAANANPLYLHYKSGWKEFKPVKANATFASAIQIGDPVSIDRAVYALRNCDGIVE
EATEEELMDAMAQADSTGMFVCPHTGVALTALFKLRSQGVIAPTDRTVVVSTAHGLKFTQ
AKIDYHSKAIPDMACRFSNPPVEVKADFGAVMDVLKGYLGSKELRS
>BnCSase27
MAEKAVTIRTRKFMTNRLLSRKQFVIDVLHPGRANVSKAELKEKLARMYEVKDPNAIFVF
KFRTHFGGGKSSGFGLIYDNVESAKKFEPKYRLIRNGLDTKIEKSRKQIKERKNRAKKIR
GVKKLIGNTPMVYLNKIVDGCVARIAAKLEMMEPCSSIKDRIAYSMIKDAEDKGLITPGK
STLIEATGGNTGIGLASIGAARGYRVILLMPSTMSLERRIILRALGAELHLTDMNIGIKG
MLEKAEEILSKTPGGYIPHQFLNPENPEIHYRTTGPEIWRDSAGEVDILVAGAGTGGTVS
GTGRFLKKMNKNIKVCVVEPTESAVLSGGEPGPHLIQGIGPGVIPTNLDLSIVDEVIQVT
SEEAIETAKLLALKEGLLVGISSGAAAAAALKVAKRPENAGKLIVVIFPSGGERYLSTEL
FESVRYEAEHLSID
>BnCSase28
MEDGCMIKKDVTELIGYTPMVYLNRIVDGCVARIAAKLEMMQPCSSVKDRIAYSMIKDAE
DKGLIKPGESTLIEPTAGNTGIGLACIGAARGYKVTLLMPSTMSLERRIILKALGAELHL
TDMSIGIKGLLEKTEEMLNKTPGGFVPQQFENLANPEIHYQTTGPEIWRDSAGKVDIFIA
GVGTGGTVTGVGRFLKEMNKDIKVIAVEPTESPVLSGGEPGRHLIQGIGAGIIPANLDLS
IVDEIIQVTGEEAIETAKLLALKEGLLVGISSGAAAAAALKVAKRPENAGKLIAVLFPSG
GERYLSTKLFDSVRFEAENLPVE
>BnCSase29
MECSIKDDVTQLIGNTPMVYLNNIVDGCVARIAAKLEMMEPCSSVKERIAYGMIKDAEDK
GLITPGKSTLIEPTSGNTGIGLAFVGAARGYKVVLTMPETMSLERKIILLALGAEVHLTD
TKKGVQGLLDKAEEILSKTPDGIILHQFKNPSNPQTHYRTTGPEIWRDSAGEVDILVAGV
GTGGTISGSGKFLKEKNKDLKVYGVEPTESAVLSGGQPGPHLIQGIGAGIVPDNLDFNVL
DEIIQVTSVEAIETARLLALKEGLLVGISSGAAAAAAIKVAKRPENAGKLIVVVFPSGGE
RYLSTQLFDSVRREAESLTFE
>BnCSase30
MASVWRRLLKTETIPRISQSTRKLFSTDGSSSFAERLRNLPKEFPATQAKRDASLLIGRT
PLVFLNRVTEGCGAYIAAKQEHFQPTCSVKDRPALAMVADAEKKNLITPGKTTLIEPTSG
NMGISMAFMAALKGYKIIMTMPSYTSLERRVTMRSFGAELVLTDPTKGMGGTVKKAYDLL
ESTPDAHMLQQFANPANTQIHYDTTGPEIWEDTLGNVDIFVMGIGSGGTVSGVGQYLKSK
NPNVKIYGVEPAESNILNGGKPGPHAITGNGVGFKPDILDMDVMESVLEVSSEDAIKMAR
ELALKEGLMVGISSGANTVAAIKLAKMPENKGKLIVTIHASFGERYLSSILFDELRKEAE
AMKPVSVD
>BnCSase31
MAASGTVASFRTSVSSSPQLTHLRSPSKALKFTPLPSSRSRPSFSVSCTIAKDPPVLMSA
GSDPSLWQRPDSFGRFGKFGGKYVPETLMHALSELETAFHSLATDDDFQRELAGILKDYV

GRESPLYFAERLTEHYRRENGEGPLIYLKREDLNHTGAHKINNAVAQALLAKRLGKKRII
AETGAGQHGVATATVCARFGLECIIYMGAQDMERQALNVFRMRLLGAEVRAVHSGTATLK
DATSEAIRDWVTNVETTHYILGSVAGPHPYPMMVRDFHAVIGKETRRQALEKWGGKPDVL
VACVGGGSNAMGLFHEFVNDTEVRLIGVEAAGFGVDSGKHAATLTKGDVGVLHGAMSYLL
QDDDGQIIEPHSISAGLDYPGVGPEHSFLKDMGRAEYFSVTDEEALEAFKRVSRLEGIIP
ALETSHALAHLEKLCPTLPDGSRVVLNFSGRGDKDVQTVAKYLEV
>BnCSase32
MASCIANDVTELIGNTPLVYLNSVAEGCVGRVAAKLEMMEPCSSVKDRIGFSMISDAEKK
GLIKPGESVLIEPTSGNTGVGLAFTAAAKGYKLIITMPASMSVERRIILLAFGVELVLTD
PAKGMKGAIAKAEEILAKTPNGYMLQQFENPANPKIHYETTGPEIWKGTDGKIDGFVSGI
GTGGTITGAGKYLKEQNPNVKLYGVEPIESAILSGGKPGPHKIQGIGAGFIPSVLEVNLI
DEVVQVSSDESIDMARLLARKEGLLVGISSGAAAAAAIKLAKRPENAGKLFVAVFPSFGE
RYLSTVLFDATRKEAESMTFEA
>BnCSase33
MDSVRLPTAPSSLRSQMLGQPLHRIPLPPCNGRSNLRFKPVIGTNLSRKNHVSPVAVITR
DETSVAPLASPQPRLKVSPSSLQYPAGYLGAVPERASDPENGSIAEAMEYLTNILSTKVY
DVAIESPLHLAKKLSERLGVRLFLKREDLQPVFSFKLRGAYNMMVKLPAEQLAKGVICSS
AGNHAQGVALSAAKLGCTAVIVMPRTTPEIKWQSVENLGATVVLVGDSYDEAQAFAKQRA
EEEGLTFIPPFDHPDVIAGQGTVGMEITRQAKGPLHAIFVPIGGGGLIAGIASYVKRVCP
EVKIIGVEPADANTMALSLHHGERVILDQVGGFADGVAVKVVGEETFRISRKLVDGVVLV
TRDAICASIKDMFEEQRNILEPAGALAIAGAEAYCKYYGLKDVNVVAITSGANMNFDKLR
IVTELANVGRQQEAVLATILPEKPGSFKKFCELVGAMNITEFKYRCGSEKESVVLYSVGV
HTPGELKALEKRMESSQLRTRNLTSSDLVKDHLRYLMGGRSSVEEEVLCQFTFPERPGAL
MNFLDSFSPRWNISLFHYRAEGGAGANVLVGIQVPEQEMEEFRNRAQVLGYEYVLVSEDT
VFKLLMH
>BnCSase34
MALSSPSLLRLLPHHPFTLTTSKRHRFLSFNHEPSSSSLVVAAVSSKPSTGTKPKSKSKS
KPPPPPPAPVTTVAHEVGTEESETVNIAEDVTQLIGSTPMVYLNRVTDGCVADVAAKLES
MEPCRSVKDRIGLSMINEAEDRGDITPRKSVLVEPTTGNTGLGIAFVAAAKGYKLIVTMP
ASINVERRMLLRALGAEIVLTSPEKGLKGAVDKAKEIVLKTKNAYMFQQFDNTANTKIHF
ETTGPEIWEDTLGNVDIFVAGIGTGGTVTGTGSFLKMMNPDIKVVGVEPSERSVISGDSP
GYVPGILDVKLLDEVFKVSNEEAIEMARRLALEEGLLVGISSGAAAVAAISLAKRAENTG
KLITVLFPSHGERYITTALFSSIHKEVQEMSH
>BnCSase35
MASSCLSFNASVSPNHDLFRRHPSSPLLRHRPVLISCTSPADGNNTTTPIETITKPRRTE
NTIRDDARLQRSTAANPFSARYVPFNAPPGSSEQYSLDEIVYRSRSGGLLDVEHDMEALK
RFDGAYWRDLFDSRVGKSTWPYGSGVWSKKEWVLPEIDDDDIVSAFEGNSNLFWAERFGK
KFLGMNDLWVKHCGISHTGSFKDLGMTVLVSQVNRLRKMKRPVVGVGCASTGDTSAALSA
YCASAGIPSIVFLPANKISMAQLVQPIANGAFVLSIDTDFDGCMKLIREITAELPIYLAN
SLNSLRLEGQKTAAIEILQQFDWQVPDWVIVPGGNLGNIYAFYKGFKMCQELGLVDRIPR
MVCAQAANANPLYLHYKSGWKDFKPMTASTTFASAIQIGDPVSIDRAVYALKKCDGIVEE
ATEEELMDAMAQADSTGMFICPHTGVALTALFKLRNRGVIAPTDRTVVVSTAHGLKFTQS
KIDYHSKAIPDMACRFSNPPVEVKADFGAVMDVLKSYLGSQTLRS
>BnCSase36
MAYFSLLHSATYFPSHSKTSFKPHSTASSTVRCTSQTTPSPAAPPQKHRRSDENIRDEAR
RRPQLQNLSARYVPFDAPPLSTESYSLDEIVYRSQSGGLLDVEHDFAALKRYDGAFWRNL
FDSRVGKTTWPYGSGVWSKKEWVLPEINDDDIVSAFEGNSNLFWAERFGKQYLQMNDLWV
KHCGISHTGSFKDLGMTVLVSQVNRLRKMNKPVVGVGCASTGDTSAALSAYCAAAGIPSI
VFLPADKISTAQLVQPIANGAFVLSIDTDFDGCMHLIREVTAELPIYLANSLNSLRLEGQ
KTAAIEILQQFNWEVPDWVIVPGGNLGNIYAFYKGFHMCKELGLVDRIPRLVCAQAANAN
PLYLHYKSGFDQDFNPMKADTTFASAIQIGDPVSIDRAVYALKKSNGIVEEATEEELMDA
TALADSTGMFICPHTGVALTALMKLRKAGVIEANDRTAVVSTAHGLKFTQSKIDYHSKSI
QEMACRLANPPVKVKAEFGSVMDVLKEYLKNNESKC

>BnCSase37
MEAKKRHEYAADLSSIKEANERIKPYIHKTPVLTSESLNSISGRSLFFKCECFQKGGAFK
FRGACNAVLALGAEQAAKGVVTHSSGNHAAALSLAAKMQGIPAYIVVPKGAPKCKVDNVI
RYGGKVIWSEATMSSREEVASRVLQETGSVLIHPYNDGRIISGQGTVALELLEQIQEIDT
IIVPISGGGLISGVALAAKSIKPSIRIIAAEPKGADDAAQSKVAGRIITLPVTNTIADGL
RASLGDLTWPVVRDMVDDVVVLEDREIIEAMRMCYEMLKVCVEPSGAIGLAAVLSTSFRS
NPCWKDCKNIGIVLSGGNVDLGVLWDSFKSSV
>BnCSase38
MEDRCLIKNDVTELIGNTPMVYLNKVVDDCLARIAAKLEMMEPCSSVKDRIAYSMIKDAE
DKGLITPGKSTLIEPTAGNTGIGLACIGAARGYKVILLMPSTVSLERRIILKALGAELHL
TDVKIGIQGMLEKTEEILSKTPGGFVPQQFENPANPEIHYRTTGPEIWRDSAGKVDILVA
GVGTGGTVSGVGKFLKEMNKDIKVCAVEPAESPVLSGGERGPHLIQGIGSGIIPTNLELS
IVDEIIQVKGEEAIETAKLLALKEGLLVGISSGAAAAAALKVAKRPENAGKLIVVVFPSG
GERYLSTKLFDSVRFEAENLLIE
>BnCSase39
MECSIKDDVTQLIGNTPMVYLNNIVDGCVARIAAKLEMMEPCSSVKERIAYGMIKDAEDK
GLITPGKSTLIEPTSGNTGIGLAFVGAAKGYKVVLTMPETMSLERKIILLALGAEVHLTD
TKKGVQGLLDKAEEILSKTPDGIILHQFKNPSNPQTHYRTTGPEIWRDSAGEVDILVAGV
GTGGTISGSGKFLKEKNKDLKVYGVEPTESAVLSGGQPGPHLIQGIGAGIVPDNLDFNVL
DEIIQVTSVEAIETARLLALKEGLLVGISSGAAAAAAIKVAKRPENAGKLIVVVFPSGGE
RYLSTQLFDSVRREAESLTFE
>BnCSase40
MDSVKLPTAPSSLRTQMSPHHFHHLPLPHRSFRLKPVIGITRSRSHHVSPVAVLFREETS
LAPLDLPLPRLKVSPNSLQYPPGYLGAVPERASDHDNGSIVEAMEYLTNILSTKVYDIAV
ESPLHLAKKLSERLGVRIFLKREDLQPVKSFKLRGAYNMMVKLTSEQLAKGVICSSAGNH
AQGVAMSAAKLGCTAVIVMPRTTPEIKWQSVEDLGATVVLVGDSYDEAQAFAKQRAEEEG
LTFIPPFDHPDVIAGQGTVGMEITRQAKGPLHAIFVPIGGGGLIAGIASYVKRVCPEVKI
IGVEPADANTMALSLHHRERVILDQVGGFADGVAVKEVGKETFRICQNLVDGVVLVTRDA
ICASIKDMFEEQRNILEPAGALALAGGEAYCKYYGLKDVNVVAITSGANMNFDKLRIVTE
LANVGRQQEAVLATLLPEKPGSFKQFCELIGPMNITEFKYRCGSEKESVVLYSVGMHTAG
ELKALEKRMESSQLRTRNLTTSDLVKDHLRYLMGGRSSVEEEVLCRFTFPERPGALMNFL
DSFSPRWNISLFHYRAEGGAGANVLVGIQVSDQEIEEFRNRAQVLGYEYVLVSEDAIFNL
LMH
>BnCSase41
MASSSLFNASLSSLNPNQHPIRRHPSPSLLRHRPVAVSCADNNTTPIETSVKPPRRTENT
IRDDARLHRSTAVNPFSARYVPFNAPPNSAEHYSLDEIVYRSRSGGLLDVEHDMGALKHF
DGAYWRDLFDSRVGKSTWPYGSGVWSKKEWVLPEIDDDDIVSAFEGNSNLFWAERFGKQF
LEMNDLWVKHCGISHTGSFKDLGMTVLVSQVNRLRKMNRPVVGVGCASTGDTSAALSAYC
AAAGIPSIVFLPANKISMAQLVQPIANGAFVLSIDTDFDGCMKLIREVTSELPIYLANSL
NSLRLEGQKTAAIEILQQFDWQPPEWVIVPGGNLGNIYAFYKGFKMCQELGLVDRIPRLV
CAQAANANPLYLHYKSGWKDFKPMTASTTFASAIQIGDPVSIDRAVYALKQCDGIVEEAT
EEELMDAMAQADSTGMFICPHTGVALTALFKLRKQGVIAPTDRTVVVSTAHGLKFTQSKV
DYHSKAIPDMACRFSNPPVEVKADFGAVMDVLKSYLGSQKLRS
>BnCSase42
MFLKPDRILTRYNYWLSGQTVSTGSIEPGFEVEPDHRFYRFVAGFTEFSQIRVLNRTGLG
FFNEPRSDRFDRPVRVLRFADHSLGSVRQRTGFTRKEFPATQAKRDASLLIGRTSLVFLN
RVTEGCKAYIAAKQEHFQPTCSIKDRPALAMVADAEKKNLITPGKTTLIEPTSGNMGISM
AFMAAMKGYRIITTMPSYTSLERRVTMRSFGAQLVLTDPAKGMAGTFKKLMTSLRVFLTL
ICCNSFANPANTQIHFDTTGPEIWEDTLGNVDIFVMGIGSGGTVSGVGQYLKSKNRNVKI
YGVEPAESNILNGGKPGPHAITGNGVGFKPDILNMDVMECVLEVSSEDAINMARELALKE
GLMVGISSGANTVAAIRLVKMPENKGKLIVTVHASFGERYLSSVLFDELRKEAEAMKPVS
VD
>BnCSase43

MATVWRRLLKTETIPRISQSTRKLFSTDASSSFADRLRNLPKDFPSTQAKRDASLLIGRT
PLVFLNRVTEGCEAYIAAKQEHFQPTCSVKDRPALAMVEDAEKKNLITPRKTTLIEPTSG
NMGISMAFIAALKGYRIIMTMPSYTSLERRVTMRSFGAELVLTDPAKGMGGTVKKAYDLL
ESTPDAHMLQQFANPANTQIHFDTTGPEIWEDTLGNVDIFVMGIGSGGTVSGVGQYLKSK
NPNVKIYGVEPAESNILNGGKPGPHAITGNGVGFKPDILDMDVMESVLEVSSEDAIKMAR
ELALKEGLMVGISSGANTVAAIRLAKMPENKGKLIVTIHASFGERYLSSVLFDELRKEAE
AMKPVSVD
>BnCSase44
MAMLMASRFNSEAKIASRFLSTLLRNQRVTASSSSSMASASSSSALLLNPLTSSTLRHFR
SSPEISSLSFSASGFPLGMKSQQSRSYGDVSKRDPCEAVKLETGADGLNIAQNVSQLIGK
TPMVYLNSMAKGCVANIAAKLEIMEPCCSVKDRIGYSMVTDAEQKGFISPGKSVLVEPTS
GNTGIGLAFIAASRGYRLILTMPSSMSMERRVLLKAFGAELVLTDPAKGMTGAVQKAEEI
LKSTPDAYMLQQFDNPANPKIHYETTGPEIWEDTKGKVDIFVAGIGTGGTITGVGRFIKE
QNPKVQVIGVEPTESDILSGGKPGPHKIQGIGAGFIPKNLDRAIMDEVIAISSEEAIETA
KQLALKEGLMVGISSGAAAAAAIMVAKRPENAGKLIAVVFPSFGERYLSTPLFQSIREEV
EKMQPEI
>BnCSase45
MAAQLLLPRPNPFTKSASAKLFFTGDDSTLKRKSKHPTRVSNGFSLRANAAFRSNHSSSV
EIPSQWYNIVSDLSVKPPPPLHPKTFEPIKPEDLAHLFPNEIIKQEATLERFIDIPEEVL
EIYKLWRPTPLIRAKRLEKLLQTPARIYFKYEGGSPAGSHKPNSAVPQAYYNAKEGVKNL
VTETGAGQWGSSLAFASSLFGLNCEVWQVANSYHQKPYRRLMMQTWGAKVHPSPSDLTEA
GRRILQVDPSSPGSLGIAISEAVEVAARNEDTKYCLGSVLNHVLLHQTVIGEECIKQMED
FGETPDVIIGCTGGGSNFAGLSFPYIREKLKGKINPVIRAVEPSACPSLTKGVYAYDFGD
TAGLTPLMKMHTLGHDFIPDPIHSGGLRYHGMAPLISHVYEQGFMEAISIPQTECFQGRA
IQFARTEGIIPAPEPTHAIAATIREALRCKETGEAKVILMAMCGHGHFDLTSYDKYLRGE
LIDLSFSEERIRESLSKVPHVV
>BnCSase46
MAAATSSSAFLLNPLTSRHRPLKYSPELSSLSLSSRKAAAFDVLPAPLSLKSQRCSSRVV
CKAVSVKPGVEGLNIAENAAQLIGKTPMVYLNNIVKGCVASVAAKLEIMEPCCSVKDRIG
YSMITDAEEKGLITPGKSVLVESTSGNTGIGLAFIAASKGYKLILTMPASMSLERRVLLR
AFGAELVLTEPAKGMTGAIQKAEEILKNTPDSYMLQQFDNPANPKIHYETTGPEIWEDTR
GKVDILVAGIGTGGTITGVGRYIKERKPELKVIGVEPTESAILSGGKPGPHKIQGIGAGF
IPKNLDQTVVDEYIAISSEEAIEFAKQLALQEGLLVGISSGAAAAAAIQVAKRPENAGKL
IAVVFPSFGERYLSTLLFQSIRDECENMQPEL
>BnCSase47
MASQLLLPTNTFTNSSLDKVFVTGDDLTLKRKPNHATRVSYGFSLRANAALNSSHSSYVE
VPRQWYNLVADLSVKPPPQLHPKTFEPIKPEDLAHLFPNEIIKQEETLERFIDIPEEVLE
IYKLWRPTPLIRAKRLEKLLHTPARIYFKYEGGSPAGSHKPNSAVPQAYYNAKEGVKNVV
TETGAGQWGSSLAFASSLFGLDCEVFQVAHTYQQKPYRRLMMQTWGAKVHRSPSELTEAG
RRILQADPSSTGSLGIAISEAVEVASRNEDTKYCLGSVFNHVLLHQTVIGEECIKQMEEY
GETPDVIIGCTGGGSNFAGLSFPYIREKLKGKINPIIRAVEPSACPSLTKGVYAYDFGDT
AGLTPLMKMHTLGHDFIPSPIHSGGLRYHGMAPLVSHIYGQGFMEAISIPQTECFQGAIQ
FARTEGIIPAPEPTHAIAATIREALRCKETGEAKVILMAMCGHGHFDLASYEKYLRGELV
DLLFSEEKIQESLSKVPLVV
>BnCSase48
MAAATSSSSGFLLNPLTSRHRPFNYSPQLASLSLSSRKAAAFTLNSQSQRCSDVVCKAVS
AKVEAGVEGLNIAENAAQLIGKTPMVYLNNIVKGCVASVAAKLEIMEPCCSVKDRIGYSM
ITDAEEKGLITPGKSVLVESTSGNTGIGLAFIAASKGYKLILTMPASMSLERRVLLRAFG
AELVLTEPAKGMTGAIQKAEEILKNTPNSYMLQQFDNPANPKIHYETTGPEIWEDTRGKV
DILVAGIGTGGTITGVGRFIKERKPELKVIGVEPTESAILAGGKPGPHKIQGIGAGFIPK
NLDQSVVDEYISISSDEAIETAKQLALQEGLLVGISSGAAAAAAIQVAKRPENAGKLIAV
VFPSFGERYLSTMLFQSIREECEKMQPEI
>BnCSase49

MSSTKIFQVRGQPLPRFPVRNHRMINTVVCGFPIISHHRVSNVLSRTSGPFLGYVPARTD
ENPFLRGDSNGRFGKFGGKFVPETLMSCLRDLEDEFNFVLSDHEFQVEFTAALRDYVGRE
TPLYFAERLTQHYKNIARTTGDGPEIYLKREDLCHGGSHKINNALAQAMIARRLGCSRVV
AATGSGQHGVATAAACAKLSLECTVFMGTTDIEKQSSNVLSMKLLGAQVKSVQGTFQDAS
SEAIRNWVGKLETTYYLPGTVVGPHPSPVMVREFQSVIGKETRRQAKQLWGGKPDVLVAC
VGSGSNALGLFHEFVRDEDVRLVGVEAAGLGLDSGKHSATLAVGDVGVYHGSMSYLLQDD
QGQILRPHSIGVGLEYPGVGPEISFLKESGRAEFCTATDQEAIQACMLLSRLEGIIPALE
TSHALAILEKLVPTLRDGAKVVVNCSGRGDKDINTLIQRGMPSSLC
>BnCSase50
MECSIKDDVTQLIGNTPMVYLNNIVDGCVARIAAKLEMMEPCSSVKERIAYGMIKDAEDK
GLITPGKSTLIEPTSGNTGIGLAFVGAARGYKVVLTLPQTMSLERKTVLIALGADVHLTD
PRRGVKGLYEKANEILSKTPDGIILHQFKNPANPQVYIMDFPYTSLSFVISFSFLFLMQT
HYRTTGPEIWRDSAGEVDILVGGVGTGGTISGAGKFLKEKKKDFKVYAVEPKESAVLSGC
KPRLHLIQGIGAGIIPDNLDFSIVDEIIQVPGLEAIETARLIALKEGLLVGISSGAAAAA
AIKVAKRPENAGKLIVVVFPSGGERYISTELFDSVRREAESMTFE
>BnCSase51
MQSHYETTGPEIWRDSAEKVDMLVVGVGTGGTISGAGKFLKEKNKDFKVYGVEPAESAVL
SGGQPGPHGIQGIGAGLIPDNLDFSVLDEVIQVTSVEAIETAKLLALKEGLLVGISSGAA
AAAAIKVAKRPENAGKLIVVVFPSGGERYLSTPLFDSIRCEAENLAIE
>BnCSase52
MSLTFWSVCVLSLSLYLPPLTPLSLYLSLLWTNLYSLELGKFITFLLNSSSVFSPQFTNI
LNNLASSGSISAQELSLFVISSNQIWEVNLAGITRSLYSMEYSIKDDVTQLIGNTPMVYL
NNIVDGCVARIAAKLEMMQPCSSVKDRIAYGMIKDAEDKGLISPGKNILIEPTSGNTGIG
IAMVGAARGYKVVITMPASVSIERRIILLALGAELHLTDPSKGVIGVIVKAEEILSKTPD
GFMLEQFRNPSNPQSHYETTGPEIWRDCAEKVDMLVVGVGTGGTISGAGKFLKEKNKDFK
VYGVEPAESAVLSGGQPGPHGIQGIGAGLIPDNLDFSVLDEVIQVTSVEAIETAKLLALK
EGLLVGISSGAAAAAAIKVAKRPENAGKLIVVVFPSGGERYLSTPMFDSIRCEAENLAIE
>BnCSase53
MAPLKITGAVVAAATMVMLSYCYLGFFRFSDKLESCSSSKKKKKTKTKTKKEKLSTRNGL
VDAIGNTPLIRINSLSEATGCEILGKCEFLNPGGSVKDRVAVKIIEEALESGKLFPGGIV
TEGSAGSTAISLATVAPAYGCQCHVVIPDDAAIEKSQIIEALGATVERVRPVSITHKDHF
VNIARRRADEANDLASSKRRLASGINIAHQGKTNGCTAEEEKEPSLSSESVTGGFFADQF
ENLANYRAHYEGTGPEIWQQTHGNIDAFVAAAGTGGTLAGVSRFLQEKNEKVKCFLIDPP
GSGLYNKVTRGVMYTREEAEGRRLKNPFDTITEGIGINRLTQNFLMAKIDGGFRGTDKEA
VEMSRFLLKKDGLFVGSSSAMNCVGAVRVAQALGPGHTIVTILCESGMRHLSKFHDPQYL
ALYGLTPTAVGLEFLGIK
>BnCSase54
MTSQLLLPPNPFTRPVSAKVFLTGDDLTLIRKSNQATRVSNGFSLRAKAALRSNHSSSVE
IPNQWYNLIADLSVKPPPPLHPKTLEPIKPEDLSHLFPNELIKQEATLERFIDIPEEVLE
IYKLWRPTPLIRGLFLFFIIPSLALSLEQRDWRSFFRHLQGFTSKYEGSSPAGSHKPNTA
VPQAYYNAKEGVKNVVTETGAGQWGSSLAFASSLFGLDCEVWQVANSYHQKPYRRLMMQT
WGAKVHPSPSDLTEAGRKILESDPSSPGSLGIAISEAVEVAARNEDTKYCLGSVLNHVLL
HQTVIGEECIKQMEDFGETPDVIIGCTGGGSNFAGLSFPFIREKLKGNISPVIRAVEPST
CPSLTKGVYAYDFGDTAGLTPLMKMHTLGHDFIPDPIHAGGLRYHGMAPLISHVYEQGFM
EAISIPQIECFQGAIQFARTEGIIPAPEPTHAIAATIREALRCKETGEAKVILMAMCGHG
HFDLSSYDKYLKGELVDLSFSEDKIRESLSKVPHVV
>BnCSase55
MTSQLLLPPNPFTRPVSAKVFLTDDDLTLKRKSNQATRVSNGFSLRAKAALRSNHSSSVE
IPNQWYNLTADLSVKPPPPLHPKTLEPIKPEDLSHLFPNELIKQEATLERFIDIPEEVLE
IYKLWRPTPLIRAKRLEKLLQTPARIYFKYEGSSPAGSHKPNTAVPQAYYNAKEGVKNVV
TETGAGQWGSSLAFASSLFGLDCEVWQVANSYHQKPYRRLMMQTWGAKVHPSPSDLTEAG
RKILESDPSSPGSLGIAISEAVEVAARNEDTKYCLGSVLNHVLLHQTVIGEECIKQMEDF
GETPDVIIGCTGGGSNFAGLSFPFIREKIKGNISPVIRAVEPSACPSLTKGVYAYDFGDT

AGLTPLMKMHTLGHDFIPDPIHAGGLRYHGMAPLISHVYEQGFMEAISIPQIECFQGAIE
FARAEGIIPAPEPTHAIAATIREALRCKETGEAKVILMAMCGHGHFDLSSYDKYLKGELV
DLSFSEDKIRESLSKVPHVV
>BnCSase56
MRRRLLTLSRVALLELELTKRRMSAATPMADFLTKSPYSPPSWASHLRPLPSHTFSLAHR
PTPIHRWNLPGLPNGTELWIKRDDFTGMELSGNKVRKLEFLMADAVEQQADTVITIGGIQ
SNHCRATTVASNYLNLDTHLILRTSKVSSFLRIISHQNLSFHLTLFLGLQLLADGDPGLV
GNLLVERLVGANVHLISKEEYSSIGSEALTSALKEKLEKEGKKPYVIPVGGSNSLGTWGY
IEAAREIEEQLKCRTDGLKFDDIVVACGSGGTIAGISLGSWLGALKAKVHAFSVCDDPDY
FYDFVQGLLDGLQAGVNSRDIVSIHNAKGKGYAMNTSEELKFLKDTASATSVILDPVYSG
KAAYGLINEMTKDPKSWEGKKILFIHTGGLLGLYDKVDQMASLMGNWSRMDVQESVPRKE
GVGKMF
>BnCSase57
MEDRCLIKNDVTELIGNTPMVYLNKIADGCVARIAAKLEMMEPCSSIKDRIAYSMIKDAE
DKGLITPGESTLIEPTAGNTGIGLACIGAARGYKVIILMSSSMSLERRIILRALGAELHL
TDRSIGFKGMLEKTEEMLSKTSGGFVPQQFENPSNPEIHYRTTGPEIWRDSAGKVDILVA
GVGTGGTVTGVGKFLKEMNQNIKIQKLHYPVLSGGEPGPHLIQGIGAGIIPTNLNLSIVD
EIIQISEEATETAKLLALKEGLLVGISSGATAAAALKVAKRPENAGKLIVVIFASGGERY
LSTKLFDSVRYEAENLQIE
>BnCSase58
MFWDARNTRKAGGDIMGVDVLLLDQKRLGKKRIIAETGAGQHGVATATVCAQYGLECIIS
MGAQDMERQALNVFRMRLLGAEVRGVHSGTATLKDATSEAIRDWVTNVETTHCILGSVAG
PHPYPMMVRDFHAVIGKETRRQALEKWGGKPDVMVACVSGGSNAMGLFHEFVDDAEVRMI
GVEAAGFGLDSGKHAAPLTKGDVGVLHGAMSYLLQDDDGQIIEPQLDYPGVGPEHSFLKD
MGRAEYYSVTDEEALEGLYQCVVSVFFIKVKTCSLTRRFVAFNRVSRLQGIISALETSHA
LPHLEKLCPTLPDGARVVLNFSGRGHKDVQTVAKYLEV
>BnCSase59
MATSGTASTFRPSVSASSRLTHLRSSPFKVPNFTPLPSSRSHSFSVSCTIAKDPTFLMAE
AEKTKAAGSDPTLWKRPDSFGRFGKFGGKYVPETLMHALSELETAFYSLATDDDFQRELA
GILKDYVGRESPLYFAERLTEHYRRENGEGPLIYLKREDLNHTGAHKINNAVAQALLAKR
LGKKRIIAETGAGQHGVATATVCARFGLQCIIYMGAQDMERQALNVFRMRLLGAEVRGVH
SGTATLKDATSEAIRDWVTNVETTHYILGSVAGPHPYPMMVRDFHAVIGKETRRQAMEKW
GGKPDVLVACVGGGSNAMGLFHEFVDDTEVRMIGVEAAGFGLDSGKHAATLTKGDVGVLH
GAMSYLLQDDDGQIIEPHSISAGLDYPGVGPEHSFLKDMGRAEYYSVTDEEALEAFKRVS
RLEGIIPALETSHALAHLEKLCPTLPDGARVVLNFSGRGDKDVQTAIKYLEV
>BnCSase60
MASRIAKDVTELIGNTPLVYLNNVAEGCVGRVAAKLEMMEPCSSVKDRIGFSMISDAEKK
GLIKPGESVLIEPTSGNTGVGLAFTAAAKGYKLIITMPASMSVERRIILLAFGVELVLTD
PAKGMKGAIAKAEEILAKTPNGYMLQQFENPANPKIHYETTGPEIWKGTDGKIDGFVSGI
GTGGTITGAGKYLKEQNPNVKLYGVEPIESAILSGGKPGPHKIQGIGAGFIPSVLEVDLI
DEVVQVSSDESIDMARLLALKEGLLVGISSGAAAAAAIKLAKRPENAGKLFVAVFPSFGE
RYLSTVLFDATRKEAESMTFQACIVSP
>BnCSase61
MPSTKIQLRWQPLPRVPERNHRMINSVVFGVPIKCHHRVSDVLSRTNGPSFGSVTSVSIR
TKARPFLLGDANGRFGRFGGKFVPETLMSRLRDLEEELDFVLSDHEFQAELTTALRDYVG
REMPLYLAERLTEHYRNKSRTTGDGPEIYLKREDLGHSGSHKMNNALAQAMIARRFGCSR
VVVATGAGQHGVATAAACAKLSLECTVFMGTTDIEKQSSNVLSMKLLGAQVSSLEGTFQD
ASSEAIRNWVENLETTYYLSGTVVGPHPSPVMVREFQSVIGKETRKQAKRLWGGKPDVLV
ACVGSGSNALGLFHEFVGDEDVRLVGVEAAGLGLDSGKHSATLAVGDVGVYHGSMSYLLQ
DDQGQILRPHSIGVELEYPGVGPEISFLKETGRVEFYTATDKEAIQACMLLSRLEGIIPA
LEASHALAFLDKLVPILHDGAKVIVNCSGRGDKDIDTLIKRGNPSSLSRVTKMSHYS
>BnCSase62
MEELTTALRDYVGRETPVYFAGRLTEHYKNISQTTGGGPEIYLKREDLSHCGSHKINNAL

GQAMIARRLGCKRVVAATGAGQQGVATAAACAKFSMECTVFMGTADIEKQSSNVLSMKLL
GAQVKSVEGTFKDASSEAIRNWVGNLETTYYLSGTVVGPHPSPLMVREFQSVIGKETRRQ
AKQLWGGKPDVLVACVGSGSNALGLFHEFLGDEDVRLVGVEAAGLGLDSGKHSATLAVGD
VGVYHGSMSYLLQDDQGQILKPHSIGVGLEYPGVGPEISFLKESGRAEFYTATGQEAVQA
CMLLSRLEGIIPALEASHALAFLDKLVPTLRDGAKVVVNCSGRGDKDLDTLIQRGMPSSL
C
>BnCSase63
MSSTKIQIRGQPLSKVLTRNHGMINSVVCGVPIKRHHRVSNVLRTSDPPLGSVPTRTDES
QFLRGDGNGRFGRFGGKFVPETLMSPLRDLEDEFNFVLNDHEFQEELTTALRDYVGRETP
LYFAGRLTEHYKNISQTTGGGPEIYLKREDLSHCGSHKINNALGQAMIARRLGCKRVVAA
TGAGQHGVATAAACAKFSMECTVFMGTADIEKQSSNVLSMKLLGAQVKSVEGTFKDAISE
AIRNWVGNLETTYYLSGTVVGPHPSPLMVREFQSVIGKETRRQAKQLWGGKPDVLVACVG
SGSNALGLFHEFLGDEDVRLVGVEAAGLGLDSGKHSATLAVGDVGVYHGSMSYLLQDDQG
QILKPHSIGVGLEYPGVGPEISFLKESGRAEFYTATDQEAVQACMLLSRLEGIIPALEAS
HALAFLDKLVPTLRDGAKVVVNCSGRGDKDLDTLIQRGMPSSLC
>BnCSase64
MATIWRRLLKTETIPRISQSTRKLFSTDGSSSFADRLRNLPKEFPATQAKRDASLLIGRT
PLVFLNRVTEGCGAYIAAKQEHFQPTCSVKDRPALAMVADAEKKNLITPGKTTLIEPTSG
NMGISMAFMAALKGYKIIMTMPSYTSLERRVTMRSFGAELVLTDPTKGMGGTVKKAYDLL
ESTPDAHMLQQFANPANTQIHYDTTGPEIWEDTLGNVDIFVMGIGSGGTVSGVGQYLKFK
NPNVKIYGVEPAESNILNGGKPGPHAITGNGVGFKPDILDMDVMESVLEVSSEDAIKMAR
ELALKEGLMVGISSGANTVAAIRLAKMPENKGKLIVTIHASFGERYLSSILFDELRKEAE
AMKPVSVD
>BnCSase65
MSWTKIQVRGQPVPRVPARNHRMINSFVCGVSIKSHHRVSNVLRTNGSPLGSVPIRTTES
QFLRGDVNGRFGRFGGKFVPETLMSLLRDLEDEFNFVLSDHEFQEELTTALRDYVGRETP
LYFAGRLTEHYKSISRTIGDGPEIYLKREDLSHCGSHKINNALAQAMIARRLGCSRVVAA
TGAGQHGVATAAACAKFSVECIVFMGTADKEKQFSNILSMKLLGAQVKSVEGTFKDASSE
AIRNWVGNLETTYYLSGTVVGPHPSPLMVREFQSVIGKETRRQANQLWGGKPDVLVACVG
SGSNALGLFHEFVGDEDVRLVGIEAAGLGLDSGKHSATLAVGDVGVYHGSMSYLLQDDEG
QILKPHSVGVGLEYPGVGPEISFLKETGRAEFYTATDQEAIQGY
>BnCSase66
MTSCSFSSSSLFSFNLPNQNSLHRRPPTLPRHAIVSSTDGSSNGASSSSSPTVKPTRTED
NIRDEARRHRSASANPFSARYVPFNAPPGSTESYSLDEVVYRSESGGLLDVQHDLDALRS
HDGAYWRNLFDSRVGKTKWPYGSGVWSKKEWVLPEIDDDDIVSAFEGNSNLFWAERFGKT
FLGMNDLWVKHCGISHTGSFKDLGMTVLVSQVNRLRKMNKPVVGVGCASTGDTSAALSAY
CASAGIPSIVILPANKISMAQLVQPIANGAFVLSIDTDFDGCMKLIREITSELPIYLANS
LNSLRLEGQKTAAIEILQQFNWQVPDWVIVPGGNLGNIYAFYKGFKMCQELGLVDRIPRL
VCAQAANANPLYLHYKSGWKEFKPVKANATFASAIQIGDPVSIDRAVYALRNCDGIVEEA
TEEELMDAMAQADSTGMFVCPHTGVALTALFKLRSQGVIAPTDRTVVVSTAHGLKFTQAK
IDYHSKAIPDMACRFSNPPVEVKADFGAVMDVLKGYLGSEELRS
>BnCSase67
MAASGTVASFRTSVSSSPQLTHLRSPSKALKFTPLPSSRSRPSFSVSCTIAKDPPVLMSA
GSDPTLWQRPDSFGRFGKFGGKYVPETLMHALSELETAFHSLATDDDFQRELAGILKDYV
GRESPLYFAERLTEHYRRENGEGPLIYLKREDLNHTGAHKINNAVAQALLAKRLGKKRII
AETGAGQHGVATATVCARFGLECIIYMGAQDMERQALNVFRMRLLGAEVRAVHSGTATLK
DATSEAIRDWVTNVETTHYILGSVAGPHPYPMMVRDFHAVIGKETRRQALEKWGGKPDVL
VACVGGGSNAMGLFHEFVDDTEIRLIGVEAAGFGVDSGKHAATLTKGDVGVLHGAMSYLL
QDDDGQIIEPHSISAGLDYPGVGPEHSFLKDMGRAEYFSVTDEEALEAFKRVSRLEGIIP
ALETSHALAHLEKLCPTLPDGSRVVLNFSGRGDKDVQTVAKYLEV
>BnCSase68
MAEKAVTIRTRKFMTNRLLSRKQFVIDVLHPGRANVSKAELKEKLARMYEVKDPNAIFVF
KFRTHFGGGKSSGFGLIYDNVESAKKFEPKYRLIRNGLDTKIEKSRKQIKERKNRAKKIR

GVKKLIGNTPMVYLNKIVDGCVARVAAKLEMMEPCSSIKDRIAYSMIKDAEDKGLITPGK
STLIEATGGNTGIGLASIGAARGYRVILLMPSTMSLERRIILRALGAEVHLTDMNIGIKG
MLEKAEEILSKTPGGYIPHQFLNPENPEIHYRTTGPEIWRDSAGEVDILVAGAGTGGTVS
GTGRFLKKMNKNIKVCVVEPTESAVLSGGEPGPHLIQGIGPGVIPTNLDLSIVDEVIQVT
SEEAIETAKLLALKEGLLVGISSGAAAAAALKVAKRPENAGKLIVVIFPSGGERYLSTEL
FEAVRYEAEHLSID
>BnCSase69
MEDGCMIKKDVTELIGYTPMVYLNRIVDGCVARIAAKLEMMQPCSSVKDRIAYSMIKDAE
DKGLIKPGESTLIEPTAGNTGIGLACIGAARGYKVTLLMPSTMSLERRIILKALGAELHL
TDMSIGIKGLLEKTEEILNKTPGGFVPQQFENPANPEIHYQTTGPEIWRDSAGKVDIFIA
GVGTGGTVTGVGRFLKEMNKDIKVFAVEPTESPVLSGGEPGRHLIQGIGAGIIPANLDLS
IVDEIIQVTGEEAIETAKLLALKEGLLVGISSGAAAAAALKVAKRPENAGKLIAVLFPSG
GERYLSTKLFDSVRFEAENLPVE
>BraA01g007870.3C
MASSCLSFNASVSPNHDLFRRHPSSPLLRHRPVLVSCTSSADGNTTTPIETVVKPRRTEN
TIRDDARLQRSTAANPFSARYVPFNAPPGSSEHYSLDEIVYRSRSGGLLDVEHDMEALKR
FDGAYWRDLFDSRVGKSTWPYGSGVWSKKEWVLPEIDDDDIVSAFEGNSNLFWAERFGKK
FLGMNDLWVKHCGISHTGSFKDLGMTVLVSQVNRLRKMKRPVVGVGCASTGDTSAALSAY
CASAGIPSIVFLPANKISMAQLVQPIANGAFVLSIDTDFDGCMKLIREITAELPIYLANS
LNSLRLEGQKTAAIEILQQFDWQVPDWVIVPGGNLGNIYAFYKGFKMCQELGLVDRIPRM
VCAQAANANPLYLHYKSGWKDFKPMTASTTFASAIQIGDPVSIDRAVYALKKCDGIVEEA
TEEELMDAMAQADSTGMFICPHTGVALTALFKLRNRGVIAPTDRTVVVSTAHGLKFTQSK
IDYHSKAIPDMACRFSNPPVEVKADFGAVMDVLKSYLGSQTLRS*
>BraA01g021750.3C
MASRIANDVTELIGNTPLVYLNSVAEGCVGRVAAKLEMMEPCSSVKDRIGFSMISDAEKK
GLIKPGESVLIEPTSGNTGVGLAFTAAAKGYKLIITMPASMSVERRIILLAFGVELVLTD
PAKGMKGAIAKAEEILAKTPNGYMLQQFENPANPKVTASYFTSAYVNMFLSVIELLTVQF
QIHYETTGPEIWKGTDGKIDGFISGIGTGGTITGAGKYLKEQNPNVKLYGVEPIESAILS
GGKPGPHKIQGIGAGFIPSVLEVNLIDEVVQVSSDESIDMARLLARKEGLLVGISSGAAA
AAAIKLAKRPENAGKLFVAVFPSFGERYLSTVLFDATRKEAESMTFEA*
>BraA01g040520.3C
MDSVRLPTAPSSLRSQMLGQPLHRIPLPPCNGRSNLRFKPVIGTNLSRKNHVSPVAVITR
DETSVAPLASPQPRLKVSPSSLQYPAGYLGAVPERTGDPENGSIAEAMEYLTNILSTKVY
DVAIESPLHLAKKLSERLGVRLFLKREDLQPVFSFKLRGAYNMMVKLPAEQLAKGVICSS
AGNHAQGVALSAAKLGCTAVIVMPRTTPEIKWQSVENLGATVVLVGDSYDEAQAFAKQRA
EEEGLTFIPPFDHPDVIAGQGTVGMEITRQAKGPLHAIFVPIGGGGLIAGIASYVKRVCP
EVKIIGVEPADANTMALSLHHGERVILDQVGGFADGVAVKVVGEETFRISRNLVDGVVLV
TRDAICASIKDMFEEQRNILEPAGALAIAGAEAYCKYYGLKDVNVVAITSGANMNFDKLR
IVTELANVGRQQEAVLATILPEKPGSFKKFCELVGSMNITEFKYRCGSEKESVCCELLLS
VGVHTPGELKALEKRMESSQLRTRNLTSSDLVKDHLRYLMGGRSSVEEEVLCQFTFPERP
GALMNFLDSFSPRWNISLFHYRAEGGAGANVLVGIQVPEQEMEEFRNRAQVLGYEYVLTK
SEEVVEAMGVFLVSMVVLELSVGTKPVSLGHRMNSGGTSASYWPWFKKMEEIVSSSLATK
CASGEDRSGRSLGNTVKPARR*
>BraA01g043580.3C
MALSSPSLLRLLPHHPFTLTTSKRHRFLSFNHEASSSSLVVAAVSSKPSTGTKPKSKSKS
KPPPPPPPAPVTTVAHEVGTEESETVNIAEDVTQLIGSTPMVYLNRVTDGCVADVAAKLE
SMEPCRSVKDRIGLSMINEAEDRGDITPRKSVLVEPTTGNTGLGIAFVAAAKGYKLIVTM
PASINVERRMLLRALGAEIVLTSPEKGLKGAVDKAKEIVLKTKNAYMFQQFDNTANTKIH
FETTGPEIWEDTLGNVDIFIVGVEPSERSVISGDSPGYVPGILDVKLLDEVFKVSNEEAI
EMARRLALEEGLLVGISSGAAAVAAISLAKRAENTGKLITVLFPSHGERYITTALFSSIH
KEVQEMSH*
>BraA02g021250.3C
MASFSLLHSATYFPSHSKTSFKPHSTASSTVRCTSQTTPSPAAPPQKHRRSDENIRDEAR

RRPQLQNLSARYVPFDAPPLSTESYSLDEIVYRSQSGGLLDVQHDFAALKRYDGAFWRNL
FDSRVGKTTWPYGSGVWSKKEWVLPEIDDDDIISAFEGNSNLFWAERFGKQYLQMNDLWV
KHCGISHTGSFKDLGMTVLVNRLRKMNKPVVGVGCASTGDTSAALSAYCAAAGIPSIVFL
PADKISTAQLVQPIANGAFVLSLDTDFDGCMHLIREVTAELPIYLANSLNSLRLEGQKTA
AIEILQQFNWEVPDWVIVPGGNLGNIYAFYKGFHMCKELGLVDRIPRLVCAQAANANPLY
LHYKSGFDQDFNPMKADTTFASAIQIGDPVSIDRAVYALKKSNGIVEEATEEELMDATAL
ADSTGMFICPHTGVALTALMKLRESGVIEANDRTVVVSTAHGLKFTQSKIEYHSKNIQEM
ACRLANPPVKVKAEFGSVMDVLKEYLKNNESKNVD*
>BraA02g028360.3C
MEAKKRHEYAADLSSIKEAHERIKPYIHKTPVLTSESLNSISGRSLFFKCECFQKGGAFK
FRGACNAVLSLDAAKGVVTHSSGNHAAALSLAAKMQGIPAYIVVPKGAPKCKVDNVIRYG
GKVIWSEASMSSREEVASRVLHETGSVLIHPYNDGRIISGQGTVALELLEQIQEIDTIIV
PISGGGLISGVALAAKSIKPSIRIIAAEPKGADDAAQSKVAGRIITLPVTNTIADGLRAS
LGDLTWPVVRDMVDDVVVVEDGEIIEAMRMCYEMLKVSVEPSGAIGLAAVLSTSFRSNPC
WKDCKNIGIVLSGGNVDLGVLWDSLKSSEILIRSLKQNLSSAASPVVVFGSSIPHIAIGA
LASYAPILHFDNIVPLTSFYDAIYQDFKVIVSLARGNSPAFFSARVTSLHTWKLTLLETI
IVAKCMA*
>BraA02g040540.3C
MEDRCLIKNDVTELIGNTPMVYLNKVVDGCLARIAAKLEMMEPCSSVKDRIAYSMIKDAE
DKGLITPGKSTLIEPTADNTGIGLACIGAARGYKVILLMPSTMSLERRIILKALGAELHL
TDVKIGIQGMLEKTEEILSKTPGGFVPQQFENPANPEIHYRTTGPEIWRDSAGKVDILVA
GVGTGGTISGVGKFLKEMNKDIKVYAVEPAESPVLSGGERGEAINITLSSKFLIITKRYK
TLVGPHLIQGIGSGIIPTNLELSIVDEIIQVKGEEAIETAKLLLALKEGLLVGISSGAAAA
AALKVAKRPENAGKLIVVVFPSGGERYLSTKLFDSVRFEAENLPTE*
>BraA03g031910.3C
MAEKAVTIRTRKFMTNRLLSRKQFVIDVLHPGRANVSKAELKEKLARMYEVKDPNAIFVF
KFRTHFGGFGLIYDNVESAKKFEPKYRLIRLIGNTPMVYLNNIVDGCVARIAAKLEMMEP
CSSVKERIAYGMIKDAEDKGLITPGKSTLIEPTSGNTGIGLAFVGAAKGYKVVLTMPETM
SLERKIILLALGAEVHLTDTKKGVQGLLDKAEEILSKTPDGIILHQFKNPSNPQTHYRTT
GPEIWRDSAGEVDILVAGVGTGGTISGSGKFLKEKNKDLKVYGVEPTESAVLSGGQPGPH
LIQGIGAGIVPDNLDFNVLDEIIQVTSVEAIETARLLALKEGLLVGISSGAAAAAAIKVA
KRPENAGKLIVVVFPSGGERYLSTQLFDSVRREAESLTFE*
>BraA03g033650.3C
MDSVKLPTAPSSLRTQMSPHHFHHLPLPHRSFPLKPVIGITRSRSHHVSPVAVLFREETS
LAPLDLPLPRLKVSPNSLQYPSGYLGAVPERASDYDNGSIVEAMEYLTNILSTKVYDIAV
ESPLHLAKKLSERLGVRMLLKREDLQPVKSFKLRGAYNMMVKLTSEQLAKGVICSSAGNH
AQGVAMSAANLGCTAVIVMPRTTPEIKWQSVEDLGAKVVLVGDSYDEAQAFAKQRAEEEG
LSFIPPFDHPDVIAGQGTVGMEITRQAKGPLHAIFVPIGGGGLIAGIASYVKRVCPEVKI
IGVEPADANTMALSLHHGERVILDQVGGFADGVAVKEVGKETFRICQNLVDGVVLVTRDA
ICASIKDMFEEQRNILEPAGALALAGAEAYCKYYGLKDVNVVAITSGANMNFDKLRIVTE
LANVGRQQEAVLATLLPEKPGSFKQFCELIGPMNITEFKYRCGSEKESVVLYSVGVHTAG
ELKALEKRMESSQLRTRNLTTSDLVKDHLRYLMGGRSSVEEEVLCQFTFPERPGALMNFL
DSFSPRWNISLFHYRAEGGAGANVLVGIQVSEQEMEEFRNRAQVLGYEYVLVSEDAIFNL
LMQ*
>BraA03g053470.3C
MATSGTASTFRPSVSASSRLTHLRSPPSKVPLFTPLPSSRSRSFSVSCTIAKDPTFLMAE
AENTKTAGSDPTLWKRPDSFGRFGKFGGKYVPETLMHALSELETAFYSLATDDDFQRELA
GILKDYVGRESPLYFAERLTEHYRRENGEGPLIYLKREDLNHTGAHKINNAVAQALLAKR
LGKKRIIAETGAGQHGVATATVCARFGLQCIIYMGAQDMERQALNVFRMRLLGAEVRGVH
SGTATLKDATSEAIRDWVTNVETTHYILGSVAGPHPYPMMVRDFHAVIGKETRRQAMEKW
GGKPDVLVACVGGGSNAMGLFHEFVDDTEVRMIGVEAAGFGLDSGKHAATLTKGDVGVLH
GAMSYLLQDDDGQIIEPHSISAGLDYPGVGPEHSFLKDMGRAEYYSVTDEEALEAFKRVS
RLEGIIPALETSHALAHLEKLCPTLPDGARVVLNFSGRGDKDVQTAIKYLEV*

>BraA04g001190.3C
MATVWRRLLKTETIPRISQSTRKLFSTDAPSSFADRLRNLPKDFPSTQAKRDASLLIGRT
PLVFLNRVTEGCEAYIAAKQEHFQPTCSVKDRPALAMVADAEKKNLITPGKTTLIEPTSG
NMGISMAFIAALKGYKIIMTMPSYTSLERRVTMRSFGAELVLTDPAKGMGGTVKKAYDLL
ESTPDAHMLQQFANPANTQIHFDTTGPEIWEDTLGNVDIFVMGIGSGGTVSGVGQYLKSK
NPNVKIYGVEPAESNILNGGKPGPHAITGNGVGFKPDILDMDVMESVLEVSSEDAIKMAR
ELALKEGLMVGISSGANTVAAIRLAKMPENKGKLIVTIHASFGERYLSSVLFDELRKEAE
AMKPVSVD*
>BraA04g002260.3C
MAMLMASRFNSEAKIASRFLSTLFRNQRVTASSSSSSSSLLLNPLTSSTLRHFRSSPEIS
SLSFSASGFPLAMKSQQQSRSYGDVSKRDPCEAVKRENGADGLNIAENVSQLIGKTPMVY
LNSMAKGCVANIAAKLEIMEPCCSVKDRIGYSMVTDAEQKGFISPGKSVLVEPTSGNTGI
GLAFIAASRGYRLILTMPSSMSMERRVLLKAFGAELVLTDPAKGMTGAVQKAEEILKSTP
DAYMLQQFDNPANPKIHYETTGPEIWEDTKGKVDIFVAGIGTGGTITGVGRFIKEKNPKV
QVIGVEPTESDILSGGKPGPHKIQGIGAGFIPKNLDRAIMDEVIAISSEEAIETAKQLAL
KEGLMVGISSGAAAAAAIMVAKRPENAGKLIAVVFPSFGERYLSTPLFQSIREEVEKMQP
EI*
>BraA04g011130.3C
MAAQLLLPPNPFTKSASAKLFFTGDDSTLKRKSKHPTRVSNGFSLRANAALRSNHSSSVE
IPSRWYNIVADLSVKPPPPLHPKTYEPIKPEDLAHLFPNEIIKQEATLERFIDIPEEVLE
IYKLWRPTPLIRAKRLEKLLQTPARIYFKYEGGSPAGSHKPNSAVPQAYYNAKEGVKNLV
TETGAGQWGSSLAFASSLFGLNCEVWQVANSYHQKPYRRLMMQTWGAKVHPSPSDLTEAG
RRILQVDPSSPGSLGIAISEAVEVAARNEDTKYCLGSVLNHVLLHQTVIGEECIKQMEAF
GETPDVIIGCTGGGSNFAGLSFPYIREKLKGKINPVIRAVEPSACPSLTKGVYAYDFGDT
AGLTPLMKMHTLGHDFIPDPIHSGGLRYHGMAPLISHVYEQGFMEAISIPQTECFQGRAI
QFARTEGIIPAPEPTHAIAATIREALRCKETGEAKVILMAMCGHGHFDLSSYDKYLRGEL
IDLSFSEERIRESLSKVPHVV*
>BraA04g014870.3C
MASQLLLPTNTFTNSSLVKVFVTGDDLTLKRKPNHATRVSYGFSLRANAALISSHRSSVE
VPRQWYNLVADLSVKPPPQLHPKTFEPIKPDDLTHLFPNEIIKQEETLERFIDIPEEVLE
IYKLWQSKSINTNCFCFDNRAKRLEKLLQTPARIYFKYEGGSPAGSHKPNSAVPQAYYNA
KEGVKNVVTETGAGQWGSSLAFASSLFGLDCEVFQVAHTYQQKPYRRLMMQTWGAKVHRS
PSELTEAGRRILQADPSSTGSLGIAISEAVEVASRNEDTKYCLGSVFNHVLLHQTQMEEY
GETPDVIIGCTGGGSNFAGLSFPYIREKLKGKINPIIRAVEPSACPSLTKGVYAYDFGDT
AGLTPLMKMHTLGHDFIPSPIHSGGLRYHGMAPLVSHIYDQGFLEAISIPQTECFQGAIQ
FARTEGIIPAPEPTHAIAATIREALRCKETGEAKVILMAMCGHGHFDLASYEKYLRGELV
DLSFSEEKIQESLSKVPLVV*
>BraA04g031460.3C
MAAATSSSSAFLLNPLTSRHRPFNYSPQLASLSLSSRKAAAFTLNSQRQRCEDVVCKAVS
AKVEAGVESLNIAENAAQLIGKTPMVYLNNIVKGCVASVAAKLEIMEPCCSVKDRIGYSM
ITDAEEKGLITPGKSVLVESTSGNTGIGLAFIAASKGYKLILTMPASMSLERRVLLRAFG
AELVLTEPAKGMTGAIQKAEEILKNTPNSYMLQQFDNPANPKIHYETTGPEIWEDTRGKV
DILVAGIGTGGTITGVGRFIKERKPELKVIGVEPTESAILAGGKPGPHKIQGIGAGFIPK
NLDQSVVDEYISISSDEAIETAKQLALQEGLLVGISSGAAAAAAIQVAKRPENAGKLIAV
VFPSFGERYLSTMLFQSIREECEKMQPEI*
>BraA05g003800.3C
MAAATSSSAFLLNPLTSRHRPFKYSPELSSLTLSSRKAATFDVLPTPLSLKRQSQRCSSG
VVCKAVSVKPEAGVEGLNIAENAAQLIGKTPMVYLNNIVKGCVASVAAKLEIMEPCCSVK
DRIGYSMITDAEEKGLITPGKSVLVESTSGNTGIGLAFIAASKGYKLILTMPASMSLERR
VLLRAFGAELVLTEPAKGMTGAIQKAEEILKNTPDSYMLQQFDNPANPKIHYETTGPEIW
EDTRGKVDILVAGIGTGGTITGVGRYIKERKPELKVIGVEPTESAILSGGKPGPHKIQGI
GAGFIPKNLDQTVVDEYIAISSEEAIEFAKQLALQEGLLVAKRPENAGKLIAVVFPSFGE
RYLSTLLFQSIRDECENMQPEL*

>BraA05g036600.3C
MDSVRLPTAPSSLRTQTLGHLLPHRLRHIPLPPCTSKPLIAMITRSRNHVSPIAVLSGNE
TSISPPDSPPPRLKVNPSSLQYPAGYLGAVPDRASDPENGSITEAMEYLTSILSTKVYDV
AIETPLHLAKKLSERLGVSMFLKREDLQPVFSFKIRGAYNMMAKLPSEQLAKGVICSSAG
NHAQGVAMSAAKLGCTAVIVMPRTTPEIKWQSVEDLGATVVLVGDTYDEAQAFAKQRAEE
EGLTFIPPFDHPDVIAGQGTVGMEITRQAKGPLHAIFVPIGGGGLIAGIAAYVKRVSPEV
KIIGVEPADANSMALSLHHGERVILNQIGGFADGVAVKEVGEETFRICRKLMDGVVLVTR
DAMCASIKDMFEEKRNILEPAGALAIAGAEAYCKYYGLKDVNVVAITSGANMNFDKLRIV
TELANVGRQQEAVLATILPEKPGSFKQFCELVGPMNITEFKYRCGSRKEAVVLYSVGVHT
PGELKALEKRMESSQLKTTNLTTSDLVKDHLRYLMGGRSSVENEVLCRFIFPERPGALMK
FLDSFSPRWNISLFHYRAEGAAGANVLVGIQVPENEIEEFRNRAQVLGYQYVLVSEDINF
KLLMQ*
>BraA06g000280.3C
MAPVKITGAVVAAAATMVMLSYCFFRFSSDKLDSCSSSSSSKKKKKLSTRNGLVDAIGNT
PLIRINSLSDATGSEILGKCEFLNPGGSVKDRVAVKIIEEALESGLLFPGGIVTEGSAGS
TAISLATVAPAYGCQCHVVIPDDAAIEKSQIIEALGATVERVRPVSITHKDHFVNIARRR
ADQANDLASSSKTRLASRINVAHQEKTNGCTAEEQKVPSLFSESVTGGFFADQFENLANY
RAHYEGTGPEIWQQTHGNIDAFVAAAGTGGTLAGVSRFLQEKNEKVKCFLIDPPGSGLFN
KVTRGVMYTREEAEGRRLKNPFDTITEGIGINRLTQNFLMAKIDGGFRGTDKEAVEMSRF
LLKKDGLFVGSSSAMNCVGAVRVAQALGPGHTIVTILCDSGMRHLSKFHDPQYLALYGLT
PTAVGLEFLGIK*
>BraA06g004560.3C
MRRLSRVVAFLELSIRTRRMSATATPMADFLTKSPYTPPSWASHLRPLPSHTFSLAHRPT
PIHRWNLPNLPNGTELWIKRDDFTGMELSGNKVRKLEFLMADAVEQQADTVITIGGIQSN
HCRATTLLADGDPGLVGNLLVERLVGANVHLISKEEYSSIGSEALTSALKEKLEKEGKKP
YVIPVGGSNSLGTWGYIEAAREIEEQLKCRTDGLKFDDIVVACGSGGTIAGISLGSWLGD
LKAKVHAFSVCDDPDYFYDFVQGLLDGLEAGVNARDIVSIHNAKGKGYAMNTSEELKFLK
DLASATSVILDPVYSGKAAYGLINEMTKDPKSWEGKKILFIHTGGLLGLYDKVDQMASLM
GNWSRMDVQESVPRKEGVGKMF*
>BraA06g034250.3C
MEDRCLIKNDVTELIGNTPMVYLNKIADGCVARIAAKLEMMEPCSSIKDRIAYSMIKDAE
DKGLITPGESTLIEPTAGNTGIGLACIGAARGYKVIILMSSSMSLERRIILRALGAELLL
TDRSIGFKGMLEKTEEMLSKTPGGFVPQQFENPSNPEVCAVEPTESPVLSGGEPGPHLIQ
GIGAGIIPTNLDLTIVDEIIQVTGEEAIETAKLLALKEGLLVGISSGATAAAALKVAKRP
ENEGKLIVVKLSLAHRNYHKLLS*
>BraA06g038470.3C
MKVQRSTIFAVNAKSNLHHFHSSQAELESVSKLLDRKWGLQSPATPIHRISGIGKLSFLN
NTRPRLGDEVSKKRSSFYILRDDLLHPLVNGNKARKLDALLPLLQDHKVTDLVTCGGCQS
AHTAAVAVSCAERGVRSHLLLRGEQPEVLTGYSLVSTMYGNVEYVPRSKYANREEMLRTH
ADLVAGEDGSVLWVKDLEAMDGFSSSEAASSRKVLIVNEGAGDALALLGMFRLVKYLSED
HLLGKKSRVKFVVDAGTGTSAVGLGVAAMSLGLPWEINAVMLADTLQNYKRHEARLLEEF
TRQFLPSIVCSSLDTIKWVERQRPRKFGKVLEGEVEMCRKIAQETGVLVDPMYTLAAWET
ATELVEDEESSIVVMLHTGGTLGMFGLAQRYKSFFTNSND*
>BraA07g019650.3C
MTSQLLLPPNPFTRPVSAKVFLTGDDLTLKRKSNQATRVSNGFSLRAKAALRSNHSSSVE
IPNQWYNLIADLSVKPPPPLHPKTFEPIKPEDLSHLFPNELIKQEATLERFIDIPEEVLE
IYKLWRPTPLIRAKRLEKLLQTPARIYFKYEGGSPAGSHKPNTAVPQAYYNAKEGVKNVV
TETGAGQWGSSLAFASSLFGLDCKVWQVANSYHQKPYRRLMMETWGAKVHPSPSDLTEAG
RKILESDPSSPGSLGIAISEAVEVAARNEDTKYCLGSVLNHVLLHQTVIGEECIKQMEDF
GETPDVIIGCTGGGSNFAGLSFPFIREKLKGNISPVIRAVEPSACPSLTKGVYAYDFGDT
AGLTPLMKMHTLGHDFIPDPIHAGGLRYHGMAPLISHVYEQGFMEAISIPQIECFQGAIQ
FARAEGIIPAPEPTHAIAATIREALRCKETGEAKVILMAMCGHGHFDLSSYDKYLKGELV
DLSFSEDKIRESLSKVPHVV*

>BraA08g010980.3C
MASRIAKDVTELIGNTPLVYLNNVAEGCVGRVAAKLEMMEPCSSVKDRIGFSMISDAEEK
GLIKPGESVLIEPTSGNTGVGLAFTAAAKGYKLIITMPASMSVERRIILLAFGVELVLTD
PAKGMKGAIAKAEEILAKTPNGYMLQQFENPANPKIHYETTGPEIWKGTDGKIDGFVSGI
GTGGTITGAGKYLKEQNPNVKLYGVEPIESAILSGGKPGPHKIQGIGAGFIPSVLEVDLI
DEVVQVSSDESIDMARLLALKEGLLVGISSGAAAAAAIKLAKRPENAGKLFVAVFPSFGE
RYLSTVLFDATRKEAESMTFQA*
>BraA08g018420.3C
MSSSSLFNASLSPLNPNQHPIRRHPSPSLLRHRPVAVSCTSDGNTTTPIETSVKPPRRTE
NTIRDDARLHRSTAVNPFSARYVPFNAPPNSSEHYSLDEIVYRSRSGGLLDVEHDMDALK
HFDGAYWRDLFDSRVGKSTWPYGSGVWSKKEWVLPEIDDDDIVSAFEGNSNLFWAERFGK
QFLEMNDLWVKHCGISHTGSFKDLGMTVLVSQVNRLRKMNRPVVGVGCASTGDTSAALSA
YCAAAGIPSIVFLPANKISMAQLVQPIANGAFVLSIDSDFDGCMKLIREVTSELPIYLAN
SLNSLRLEGQKTAAIEILQQFDWQAPEWVIVPGGNLGNIYAFYKGFKMCQELGLVDRIPR
LVCAQAANANPLYLHYKSGWKDFKPMTASTTFASAIQIGDPVSIDRAVYALKQCDGIVEE
ATEEELMDAMAQADSTGMFICPHTGVALTALFKLRKQGVIAPTDRTVVVSTAHGLKFTQS
KVDYHSKAIPDMACRFSNPPVEVKADFGAVMDVLKSYLGSQKLRS*
>BraA08g031560.3C
MSSTKIQIRGQPLFKVLTRNHRMINSVVCGVPIKRQHRVSNVLRTSDPPLGSVPTRTDES
QFLRGDGNGRFGRFGGKFVPETLMSPLRDLEDEFDFVLNDHEFQEELTTALRDYVGRETP
LYFAGRLTEHYKNISQTTGGGPEIYLKREDLSHCGSHKINNALGQAMIARRLGCKRVVAA
TGAGQHGVATAAACAKLSIECTVFMGTTDIEKHSSNVLSMKLLGAQVKSVEGTFKDASSE
AIRNWVGNLETTYYLSGTVVGPHPNPLMVREFQSVIGKETRRQAKQLWGGKPDVLVACVG
SGSNALGLFHEFLGDEDVRLVGVEAAGLGLDSGKHSATLAVGDVGVYHGSMSYLLQDDQG
QILKPHSIGVGLEYPGVGPEISFLKESGRAEFYTATDQEAVQACMLLSRLEGIIPALEAS
HALAFLDKLVPTLRDGAKVVVNCSGRGDKDLDTLIQRGMPSSLC*
>BraA08g031570.3C
MSSTKIQLRWQPLPRVPARNHRMINSVVFGVPIKSHHRVSDVLSRTSGPSFGSVTSVSVR
TNARPFLRGDGNGRFGRFGGKFVPETLMSRLRDLEEELDFVLSDHEFQAELTTALRDYVG
RETPLYLAGRLTEHYRNKSRTTGDGPEIYLKREDLGHSGSHKMNNALAQAMIARRLGCSR
VVAATGAGQHGVATAAACAKLCLECTVFMGTTDIEKQSSNVLSMKLLGAQVSSLEGTFQD
ASSEAIRNWIENLETTYYLSGTVVGPHPSPVMVREFQSVIGKETRRQAKRLWGGKPDVLV
ACVGSGSNALGLFNEFVGDEDVRLVGVKAAGLGLDSGKHSATLAVGDVGVYHGSMSYLLQ
DDQGQILRPHSIGVGLEYPGVGPEKSFLKETGRVEFYTATDKEAIQACMLLSRLEGIIPA
LEASHALAFLDKLVPILHDGAKVIVNCNGRGDKDIDTLIQRGMPSSLSRVTKMSHYT*
>BraA09g004810.3C
MEDRYMIKNDVTELIGNTPMVYLNKIVDGCVARIAAKLEMMEPCSSIKDRIAYSMIKDAE
DKGLITPGKSTLIEATGGNTGIGLASIGAARGYRVILLMPSTMSLERRIILRALGAELHL
TDMNIGIKGMLEKAEEILSKTPGGYIPHQFLNPENPEIHYRTTGPEIWRDSAGEVDILVA
GAGTGGTVSGTGRFLKKMNKNIKVCVVEPTESAVLSGGEPGPHLIQGIGPGVIPTNLDLS
IVDEVIQVTSEEAIETAKLLALKEGLLVGISSGAAAAAALKVAKRPENAGKLIVVIFPSG
GERYLSTELFESVRYEAEHLSID*
>BraA09g004820.3C
MEDGCMIKKDVTELIGYTPMVYLNRIVDGCVARIAAKLEMMQPCSSVKDRIAYSMIKDAE
DKGLIKPGESTLIEPTAGNTGIGLACIGAARGYKVTLLMPSTMSLERRIILKALGAELHL
TDMSIGIKGLLEKTEEMLNKTPGGFVPQQFENLANPEIHYQTTGPEIWRDSAGKVDIFIA
GVGTGGTVTGVGRFLKEMNKDIKVIAVEPTESPVLSGGEPGRHLIQGIGAGIIPANLDLS
IVDEIIQVTGEEAIETAKLLALKEGLLVGISSGAAAAAALKVAKRPENAGKLIAVLFPSG
GERYLSTKLFDSVRFEAENLPVE*
>BraA09g011320.3C
MSWTKIQVRGQPIPRVPARNHRMINSFVCGVSIKSHHRVSNVLRTNGSSLGSVPIRTTES
QFLRGDGNGRFGRFGGKFVPETLMSPLKDLEDEFNFVLGDHEFQEELTTALRDYAGRETP
LYFAGRLTEHYKSISRTTGDGPEIYLKREDLSHCGSHKINNALAQAMIARRLGCSRVVAA

TGAGQHGVATAAACAKFSLECTVFMGTADKEKQFSNVLSMKMLGAQVKSVEGTFKDASSE
AIRNWVGNLKTTYYLSGTVVGPHPSPLMVREFQSLIGKETRRQANQLWGGKPDVLVACVG
SGSNALGLFHEFVGDEDVRLVGVEAAGLGLDSGKLSATLAVGDVGVYHGSMSYLLQDDEG
QILKPHSVGVGLEYPGVGPEISFLKEAGRAEFYTATDQEAIQACRLLSRLEGIIPALEPS
HALAFLDKLVPTLRDGAKVVVNCSGRGDKDLDTLIQRGLPSSLC*
>BraA09g011810.3C
MTSCSFSSSSLFSFNLPNQNSLHRRPPTIPRHVILSSTDGSSNGASSSSPPTVKPSRTED
NIRDEARRHRSTSANPFSARYVPFNAPPGSTESYSLDDIVYRSESGGLLDVQHDLDALRA
HDGAYWRNLFDSRVGKTKWPYGSGVWSKKEWVLPEIDHDDIVSAFEGNSNLFWAERFGKT
FLGMNDLWVKHCGISHTGSFKDLGMTVLVSQVNRLRKMNKPVVGVGCASTGDTSAALSAY
CASAGIPSIVILPANKISMAQLVQPIANGAFVLSIDTDFDGCMKLIREITSELPIYLANS
LNSLRLEGQKTAAIEILQQFNWQVPDWVIVPGGNLGNIYAFYKGFKMCQELGLVDRIPRL
VCAQAANANPLYLHYKSGWKEFKPVKANATFASAIQIGDPVSIDRAVYALRNCDGIVEEA
TEEELMDAMAQADSTGMFVCPHTGVALTALFKLRSQGVIAPTDRTVVVSTAHGLKFTQAK
IDYHSKAIPDMACRFSNPPVEVKADFGAVMDVLKGYLGSEELRL*
>BraA09g050980.3C
MASVWRRLLKTETIPRISQSTRKLFSTDGSSSFAERLRNLPKEFPATQAKGDASLLIGRT
PLVFLNRVTEGCGAYIAAKQEHFQPTCSVKDRPALAMVADAEKKNLITPGKTTLIEPTSG
NMGISMAFMAALKGYKIIMTMPSYTSLERRVTMRSFGAELVLTDPTKGMGGTVKKAYDLL
ESTPDAHMLQQFANPANTQIHYDTTGPEIWEDTLGNVDIFVMGIGSGGTVSGVGQYLKSK
NPNVKIYGVEPAESNILNGGKPGPHAITGNGVGFKPDILDMDVMESVLEVSSEDAIKMAR
ELALKEGLMVGISSGANTVAAIKLAKMPENKGKLIVTIHASFGERYLSSILFDELRKEAE
AMKPVSVD*
>BraA10g012880.3C
MAASGTVASFRTSVSSSPQLTHLRSPSKALKFTPLPSSRSRPSFSVSCTIAKDPPVLMSA
GSDPSLWQRPDSFGRFGKFGGKYVPETLMHALSELETAFHSLATDDDFQRELAGILKDYV
GRESPLYFAERLTEHYRRENGEGPLIYLKREDLNHTGAHKINNAVAQALLAKRLGKKRII
AETGAGQHGVATATVCARFGLECIIYMGAQDMERQALNVFRMRLLGAEVRAVHSGTATLK
DATSEAIRDWVTNVETTHYILGSVAGPHPYPMMVRDFHAVIGKETRRQALEKWGGKPDVL
VACVGGGSNAMGLFHEFVNDTEVRLIGVEAAGFGVDSGKHAATLTKGDVGVLHGAMSYLL
QDDDGQIIEPHSISAGLDYPGVGPEHSFLKDMGRAEYFSVTDEEALEAFKRVSRLEGIIP
ALETSHALAHLEKLCPTLPDGSRVVLNFSGRGDKDVQTVAKYLEV*
>BolC1t00924H
MASSCLSFNASVSPNHDLFRRHPSSPLLRHRPVLISCTSPADGNNTTTPIETITKPRRTE
NTIRDDARLQRSTAANPFSARYVPFNAPPGSSEQYSLDEIVYRSRSGGLLDVEHDMEALK
RFDGAYWRDLFDSRVGKSTWPYGSGVWSKKEWVLPEIDDDDIVSAFEGNSNLFWAERFGK
KFLGMNDLWVKHCGISHTGSFKDLGMTVLVSQVNRLRKMKRPVVGVGCASTGDTSAALSA
YCASAGIPSIVFLPANKISMAQLVQPIANGAFVLSIDTDFDGCMKLIREITAELPIYLAN
SLNSLRLEGQKTAAIEILQQFDWQVPDWVIVPGGNLGNIYAFYKGFKMCQELGLVDRIPR
MVCAQAANANPLYLHYKSGWKDFKPMTASTTFASAIQIGDPVSIDRAVYALKKCDGIVEE
ATEEELMDAMAQADSTGMFICPHTGVALTALFKLRNRGVIAPTDRTVVVSTAHGLKFTQS
KIDYHSKAIPDMACRFSNPPVEVKADFGAVMDVLKSYLGSQTLRS*
>BolC1t02754H
MASCIANDVTELIGNTPLVYLNSVAEGCVGRVAAKLEMMEPCSSVKDRIGFSMISDAEKK
GLIKPGESVLIEPTSGNTGVGLAFTAAAKGYKLIITMPASMSVERRIILLAFGVELVLTD
PAKGMKGAIAKAEEILAKTPNGYMLQQFENPANPKIHYETTGPEIWKGTDGKIDGFVSGI
GTGGTITGAGKYLKEQNPNVKLYGVEPIESAILSGGKPGPHKIQGIGAGFIPSVLEVNLI
DEVVQVSSDESIDMARLLARKEGLLVGISSGAAAAAAIKLAKRPENAGKLFVAVFPSFGE
RYLSTVLFDATRKEAESMTFEA*
>BolC1t05423H
MDSVRLPTAPSSHRTQMLGQPLHHIPLPRPCNRRGNLRLKPVIGTTLSRNHVSPVAVITR
DETTSVSPLASPQPRLKVSPSSLQYPAGYLGAVPERASDPENGSIAEAMEYLTNILSTKV
YDVAIETPLHLAKKLSERLGVRLFLKREDLQPVFSFKLRGAYNMMVKLPAEQLAKGVICS

SAGNHAQGVALSAAKLGCTAVIVMPRTTPEIKWQSVENLGATVVLVGDSYDEAQAFAKQR
AEEEGLTFIPPFDHPDVIAGQGTVGMEITRQAKGPLHAIFVPIGGGGLIAGIASYVKRVC
PEVKIIGVEPADANTMALSLHHGERVILDQVGGFADGVAVKVVGEETFRISRKLVDGVVL
VTRDAICASIKDMFEEQRNILEPAGALAIAGAEAYCKYYGLKDVNVVAITSGANMNFDKL
RIVTELANVGRQQEAVLATILPEKPGSFKKFCELVGAMNITEFKYRCGSEKESCCVLLLS
VGVHTPGELKALEKRMESSQLRTRNLTSSDLVKDHLRYLMGGRSSVEEEVLCQFTFPERP
GALMNFLDSFSPRWNISLFHYRAEGGAGANVLVGIQVPEQEMVEFRNRAQVLGYEHRMNS
GGTSASYWPWFKKMEEIVSSSLATKCASGEDRSGRSLGNTVKPARR*
>BolC1t05821H
MALSSPSLLRLLPHHPFTLTTSKRHRFLSLNHEPSSSSLVVAAVSSKPSTRTKQKSKSKS
KPPPPPPPPVTTVSHEVGTEDSETVNIAEDVTQLIGSTPMVYLNRVTDGCVADVAAKLES
MEPCRSVKDRIGLSMINEAEDRGDITPRKSVLVEPTTGNTGLGIAFVAAAKGYKLIVTMP
ASINVERRMLLRALGAEIVLTSPEKGLKGAVEKAKEIVLKTKNAYMFQQFDNTANTKIHF
ETTGPEIWEDTMGNVDIFVAGIGTGGTVTGTGSFLKMMNPDIKVVGVEPSERSVISGDSP
GYVPGILDVKLLDEVFKVSNEEAIEMARRLALEEGLLVGISSGAAAVAAISLAKRAENTG
KLITVLFPSHGERYITTALFSSINKEVQEMSH*
>BolC2t09082H
MASFSLLHSATYFPSHSKTSFKPHSTVRCTSPTTPSPAAPPQKHRRSDENIRDEARRRPQ
LQNLSARYVPFDAPPLSTESYSLDEIVYRSQSGGLLDVQHDFAALKRYDGAFWRNLFDSR
VGKTTWPYGSGVWSKKEWVLPEINDDDIVSAFEGNSNLFWAERFGKQYLQMNDLWVKHCG
ISHTGSFKDLGMTVLVSQVNRLRKMNKPVVGVGCASTGDTSAALSAYCAAAGIPSIVFLP
ADKISTAQLVQPIANGAFVLSLDTDFDGCMHLIREVTAELPIYLANSLNSLRLEGQKTAA
IEILQQFNWEVPDWVIVPGGNLGNIYAFYKGFHMCKELGLVDRIPRLVCAQAANANPLYL
HYKSGFDQDFNPMKADTTFASAIQIGDPVSIDRAVYALKKSNGIVEEATEEELMDATALA
DSTGMFICPHTGVALTALMKLRKAGVIEANDRTVVVSTAHGLKFTQSKIDYHSKNIQEMA
CRLANPPVKVKAEFGSVMDVLKEYLKNNESKW*
>BolC2t10183H
MEAKKRHEYAADLSSIKEANERIKPYIHKTPVLTSESLNSISGRSLFFKCECFQKGGAFK
FRGACNAVLALGAEQAAKGVVTHSSGNHAAALSLAAKMQGIPAYIVVPKGAPKCKVDNVI
RYGGKVIWSEATMSSREEVASRVLQETGSVLIHPYNDGRIISGQGTVALELLEQIQEIDT
IIVPISGGGLISGVALAAKSIKPSIRIIAAEPKGADDAAQSKVAGRIITLPVTNTIADGL
RASLGDLTWPVVRDMVDDVVVLEDREIIEAMRMCYEMLKVCVEPSGAIGLAAVLSTSFRS
NPCWKDCKNIGIVLSGGNVDLGVLWDSFKSSV*
>BolC2t11851H
MEDRCLIKNDVTELIGNTPMVYLNKVVDDCLARIAAKLEMMEPCSSVKDRIAYSMIKDAE
DKGLITPGKSTLIEPTAGNTGIGLACIGAARGYKVILLMPSTMSLERRIILKALGAELHL
TDVKIGIQGMLEKTEEILSKTPGGFVPQQFENPANPEIHYRTTGPEIWRDSAGKVDILVA
GVGTVSGVGKFLKEMNKDIKVCAVEPAESPVLSGGERGPHLIQGIGSGIIPTNLELSIVD
EIIQVKGEEAIETAKLLALKEGLLVGISSGAAAAAALKVAKRPENAGKLIVVVFPSGGER
YLSTKLFDSVRFEAENLLIE*
>BolC3t16411H
MECSIKDDVTQLIGNTPMVYLNNIVDGCVARIAAKLEMMEPCSSVKERIAYGMIKDAEDK
GLITPGKSTLIEPTSGNTGIGLAFVGAAKGYKVVLTMPETMSLERKIILLALGAEVHLTD
TKKGVQGLLDKAEEILSKTPDGIILHQFKNPSNPQTHYRTTGPEIWRDSAGEVDILVAGV
GTGGTISGSGKFLKEKNKDLKVYGVEPTESAVLSGGQPGPHLIQGIGAGIVPDNLDFNVL
DEIIQVTSVEAIETARLLALKEGLLVGISSGAAAAAAIKVAKRPENAGKLIVVVFPSGGE
RYLSTQLFDSVRREAESLTFE*
>BolC3t16609H
MDSVKLPTAPSSLRTQMSPHHFHHLPLPHRSFRLKPVIGITRSRSHHVSPVAVLFREETS
LAPLDLPLPRLKVSPNSLQYPPGYLGAVPERASDHDNGSIVEAMEYLTNILSTKVYDIAV
ESPLHLAKKLSERLGVRIFLKREDLQPVKSFKLRGAYNMMVKLTSEQLAKGVICSSAGNH
AQGVAMSAAKLGCTAVIVMPRTTPEIKWQSVEDLGATVVLVGDSYDEAQAFAKQRAEEEG
LTFIPPFDHPDVIAGQGTVGMEITRQAKGPLHAIFVPIGGGGLIAGIASYVKRVCPEVKI

IGVEPADANTMALSLHHRERVILDQVGGFADGVAVKEVGKETFRICQNLVDGVVLVTRDA
ICASIKDMFEEQRNILEPAGALALAGGEAYCKYYGLKDVNVVAITSGANMNFDKLRIVTE
LANVGRQQEAVLATLLPEKPGSFKQFCELIGPMNITEFKYRCGSEKESVVLYSVGMHTAG
ELKALEKRMESSQLRTRNLTTSDLVKDHLRYLMGGRSSVEEEVLCRFTFPERPGALMNFL
DSFSPRWNISLFHYRAEGGAGANVLVGIQVSDQEIEEFRNRAQVLGYEYVLVSEDAIFNL
LMH*
>BolC3t21220H
MASSSLFNASLSSLNPNQHPIRRHPSPSLLRHRPVAVSCADNNTTPIETSVKPPRRTENT
IRDDARLHRSTAVNPFSARYVPFNAPPNSAEHYSLDEIVYRSRSGGLLDVEHDMGALKHF
DGAYWRDLFDSRVGKSTWPYGSGVWSKKEWVLPEIDDDDIVSAFEGNSNLFWAERFGKQF
LEMNDLWVKHCGISHTGSFKDLGMTVLVSQVNRLRKMNRPVVGVGCASTGDTSAALSAYC
AAAGIPSIVFLPANKISMAQLVQPIANGAFVLSIDTDFDGCMKLIREVTSELPIYLANSL
NSLRLEGQKTAAIEILQQFDWQPPEWVIVPGGNLGNIYAFYKGFKMCQELGLVDRIPRLV
CAQAANANPLYLHYKSGWKDFKPMTASTTFASAIQIGDPVSIDRAVYALKQCDGIVEEAT
EEELMDAMAQADSTGMFICPHTGVALTALFKLRKQGVIAPTDRTVVVSTAHGLKFTQSKV
DYHSKAIPDMACRFSNPPVEVKADFGAVMDVLKSYLGSQKLRP*
>BolC4t22101H
MAAATSSSAFLLNPLTSRHRPLKYSPELSSLSLSSRKAAAFDVLPAPLSLKSQRCSSRVV
CKAVSVKPGVEGLNIAENAAQLIGKTPMVYLNNIVKGCVASVAAKLEIMEPCCSVKDRIG
YSMITDAEEKGLITPGKSVLVESTSGNTGIGLAFIAASKGYKLILTMPASMSLERRVLLR
AFGAELVLTEPAKGMTGAIQKAEEILKNTPDSYMLQQFDNPANPKIHYETTGPEIWEDTR
GKVDILVAGIGTGGTITGVGRYIKERKPELKVIGVEPTESAILSGGKPGPHKIQGIGAGF
IPKNLDQTVVDEYIAISSEEAIEFAKQLALQEGLLVGISSGAAAAAAIQVAERPENAGKL
IAVVFPSFGERYLSTLLFQSIRNECENMQPEL*
>BolC4t24678H
LLKTLTIPHISQSTRKPFFTDASSSFAERLKNLPKEFPATQAKRDASLLIGRTSLVFLNR
VTEGCKAYIAAKQEHFQPTCSIKDRPALAMVADAEKKNLITPGKTTLIEPTSGNMGISMA
FMAAMKGYRIITTMPSYTSLERRVTMRSFGAQLVLTDPAKGMAGTFKKAYDLLESIPNAH
MLQQFANPANTQIHFDTTGPEIWEDTLGNVDIFVMGIGSGGTVSGVGQYLKSKNPNVKIY
GVEPAESNILNGGKPGPHAITGNGVGFKPDILNMDVMESVLEVSSEDAINMARELALKEG
LMVGISSGANTVAAIRLVKMPENKGKLIVTVHASFGERYLSSVLFDELRKEAEAMKPVSV
D*
>BolC4t24682H
MATVWRRLLKTETIPRISQSTRKLFSTDASSSFADRLRNLPKDFPSTQAKRDASLLIGRT
PLVFLNRVTEGCEAYIAAKQEHFQPTCSVKDRPALAMVEDAEKKNLITPRKTTLIEPTSG
NMGISMAFIAALKGYRIIMTMPSYTSLERRVTMRSFGAELVLTDPAKGMGGTVKKAYDLL
ESTPDAHMLQQFANPANTQIHFDTTGPEIWEDTLGNVDIFVMGIGSGGTVSGVGQYLKSK
NPNVKIYGVEPAESNILNGGKPGEYTNIESLLLYLVLNLGLGPHAITGNGVGFKPDILDM
DVMESVLEVSSEDAIKMARELALKEGLMVGISSGANTVAAIRLAKMPENKGKLIVTIHAS
FGERYLSSVLFDELRKEAEAMKPVSVD*
>BolC4t24863H
MAMLMASRFNSEAKIASRFLSTLLRNQRVTASSSSSMASASSSSALLLNPLTSSTLRHFR
SSPEISSLSFSASGFPLGMKSQQSRSYGDVSKRDPCEAVKLETGADGLNIAQNVSQLIGK
TPMVYLNSMAKGCVANIAAKLEIMEPCCSVKDRIGYSMVTDAEQKGFISPGKSVLVEPTS
GNTGIGLAFIAASRGYRLILTMPSSMSMERRVLLKAFGAELVLTDPAKGMTGAVQKAEEI
LKSIPDAYMLQQFDNPANPKIHYETTGPEIWEDTKGKVDIFVAGIGTGGTITGVGRFIKE
QNPKVQVIGVEPTESDILSGGKPGPHKIQGIGAGFIPKNLDRAIMDEVIAISSEEAIETA
KQLALKEGLMVGISSGAAAAAAIMVAKRPENAGKLIAVSRVLSDIYQPLCSSLSGKRLRK
CSLRYEQKQISFFYC*
>BolC4t26102H
MAAQLLLPRPNPFTKSASAKLFFTGNCDDSTLKRKSKHPTRVSNGFSLRANAAFRSNHSS
SVEIPSQWYNIVSDLSVKPPPPLHPKTFEPIKPEDLAHLFPNEIIKQEATLERFIDIPEE
VLEIYKLWRPTPLIRAKRLEKLLQTPARIYFKYEGGSPAGSHKPNSAVPQAYYNAKEGVK

NLVTETGAGQWGSSLAFASSLFGLNCEVWQVANSYHQKPYRRLMMQTWGAKVHPSPSDLT
EAGRRILQVDPSSPGSLGIAISEAVEVAARNEDTKYCLGSVLNHVLLHQTVIGEECIKQM
EDFGETPDVIIGCTGGGSNFAGLSFPYIREKLKGKINPVIRAVEPSACPSLTKGVYAYDF
GDTAGLTPLMKMHTLGHDFIPDPIHSGGLRYHGMAPLISHVYEQGFMEAISIPQTECFQG
RAIQFARTEGIIPAPEPTHAIAATIREALRCKETGEAKVILMAMCGHGHFDLTSYDKYLR
GELIDLSFSEERIRESLSKVPHVV*
>BolC4t26551H
MASQLLLPTNTFTNSSLDKVFVTGDDLTLKRKPNHATRVSYGFSLRANAALNSIHSSYVE
VPRQWYNLVADLSVKPPPQLHPKTFEPIKPEDLAHLFPNEIIKQEETLERFIDIPEEVLE
IYKLWRPTPLIRAKRLEKLLQTPARIYFKYEGGSPAGSHKPNSAVPQAYYNAKEGVKNVV
TETGAGQWGSSLAFASSLFGLDCEVFQVAHTYQQKPYRRLMMQTWGAKVHRSPSELTEAG
RRILQADPSSTGSLGIAISEAVEVASRNEDTKYCLGSVFNHVLLHQTVIGEECIKQMEEY
GETPDVIIGCTGGGSNFAGLSFPYIREKLKGKINPIIRAVEPSACPSLTKGVYAYDFGDT
AGLTPLMKMHTLGHDFIPSPIHSGGLRYHGMAPLVSHIYEQGFMEAISIPQTECFQGAIQ
FARTDGIIPAPEPTHAIAATIREALRCKETGEAKVILMAMCGHGHFDLASYEKYLRGELV
DLSFSEEKIQESLSKVPLVV*
>BolC4t28576H
MAAATSSSSGFLLNPLTSRHRPFNYSPQLASLSLSSRKAAAFTLNSQSQRCSDVVCKAVS
AKVEAGVEGLNIAENAAQLIGKTPMVYLNNIVKGCVASVAAKLEIMEPCCSVKDRIGYSM
ITDAEEKGLITPGKSVLVESTSGNTGIGLAFIAASKGYKLILTMPASMSLERRVLLRAFG
AELVLTEPAKGMTGAIQKAEEILKNTPNSYMLQQFDNPANPKIHYETTGPEIWEDTRGKV
DILVAGIGTGGTITGVGRFIKERKPELKVIGVEPTESAILAGGKPGPHKIQGIGAGFIPK
NLDQSVVDEYISISSDEAIETAKQLALQEGLLVGISSGAAAAAAIQVAKRPENAGKLIAV
SPQSLGNVTSRQCFSSRSVKNARKCSLRFEFPLVVS*
>BolC5t29715H
MSSTKIFQVRGQPLPRFPVRNHRMINTVVCGFPIISHHRVSNVLSRTSGPFLGYVPARTD
ENPFLRGDSNGRFGKFGGKFVPETLMSCLRDLEDEFNFVLSDHEFQVEFTAALRDYVGRE
TPLYFAERLTQHYKNIARTTGDGPEIYLKREDLCHGGSHKINNALAQAMIARRLGCSRVV
AATGSGQHGVATAAACAKLSLECTVFMGTTDIEKQSSNVLSMKLLGAQVKSVQGTFQDAS
SEAIRNWVGKLETTYYLPGTVVGPHPSPVMVREFQSVIGKETRRQAKQLWGGKPDVLVAC
VGSGSNALGLFHEFVRDEDVRLVGVEAAGLGLDSGKHSATLAVGDVGVYHGSMSYLLQDD
QGQILRPHSIGVGLEYPGVGPEISFLKESGRAKFCTATDQEAIQACMLLSRLEGIIPALE
TSHALAILEKLVPTLRDGAKVVVNCSGRGDKDINTLIQRGMPSSLC*
>BolC5t34459H
MDSVRLPTAPSTLRSQMLGHTLPHRLPRIPLPPCNRHFESKPLIAITRSRNRVSPIAVIS
GNETSISPPDSPPPRLKVNPSSLQYPAGYLGAVPDRASDPENGSITEAMEYLTSILSTKV
YDVAIETPLHLAKKLSERLGVSMFLKREDLQPVFSFKIRGAYNMMAKLPSEQLAKGVICS
SAGNHAQGVAMSAAKLGCTAVIVMPRTTPEIKWQSVEDLGATVVLVGDTYDEAQAFAKQR
AEEEGLTFIPPFDHPDVIAGQGTVGMEITRQAKGPLHAIFVPIGGGGLIAGIAAYVKRVS
PEVKIIGVEPADANSMALSLHHGERVILNQIGGFADGVAVKEVGEETFRICRKLIDGVVL
VTRDAMCASIKDMFEEKRNILEPAGALAIAGAEAYCKYYGLKDVNVVAITSGANMNFDKL
RIVTELANVGRQQEAVLATILPEKPGSFKQFCELVGPMNITEFKYRCGSRKDAVVLYSVG
VHTPGELKALEKRMESSQLKTTNLTTSDLVKDHLRYLMGGRSSVENEVLCRFIFPERPGA
LMKFLDSFSPRWNISLFHYRAEGAAGANVLVGIQVPENEMEEFRNRAQVLGYEYVLVSED
INFKLLMQ*
>BolC5t34893H
MTSDQRDVVFSLESPDLEEGAAGESDTDENDDVAIPETNEEPEEDDPEEEDLTTAVTVSA
SSTDAVTVALPAGSAVPVSVIPVDSSDPKWHRVTEIVHHLRPPPPPQPQPQPPIDDSRRL
FQRLWTDEDEIELLRGFLDYVATHRGGNSSHPPDTAPFYEMIKSKLQLEFNKNQLVEKLR
RLKKKYRNVMSKISSGKEVFFKSPHDQSTFEISRKIWNQTGKIIGFEDNNAMDFEETNTN
GNYFNSPGSNPTPSNVEIDSENGVEKRLMMMSSSSGGSRKRSRSRIGKIEEDNKPVITPS
DVQTPNAASNVNLNEPATAVVGGNIGVLIEETVKNCVSPVIKEMMNGTTSMMMAAMGGGG
GNGHGFGSFSPVFTRPLGYGVEGGGGNKAVSDERWRKQQILELEVYSRRLELVQEQIRAT

LHELKTMPSGSLFVISSNQIWEVNLAGITRSLYSMEYSIKDDVTQLIGNTPMVYLNNIVD
GCVARIAAKLEMMQPCSSVKDRIAYGMIKDAEDKGLISPGKNILIEPTSGNTGIGIAMVG
AARGYKVVITMPASVSIERRIILLALGAELHLTDPSKGVIGVIVKAEEILSKTPDGFMPE
QFRNPSNPQSHYDTTGPEIWRDSAEKVDMLVVGVGTGGTISGAGKFLKEKNKDFKAYKLK
FKISYMVIMDPMAAHENMGVYGVEPAESAVLSGGQPGPHGIQGIGAGLIPDNLDFSVLDE
VIQVTSVEAIETAKLLALKEGLLVGISSGAAAAAAIKVAKRPENAGKLIVVVFPSGGERY
LSTPMFDSIRCEAENLAIE*
>BolC6t35420H
MRRRLLTLSRVALLELELTKRRMSAATPMADFLTKSPYSPPSWASHLRPLPSHTFSLAHR
PTPIHRWNLPGLPNGTELWIKRDDFTGMELSGNKVRKLEFLMADAVEQQADTVITIGGIQ
SNHCRATTVASNYLNLDTHLILRTSKLLADGDPGLVGNLLVERLVGANVHLISKEEYSSI
GSEALTSALKEKLEKEGKKPYVIPVGGSNSLGTWGYIEAAREIEEQLKCRTDGLKFDDIV
VACGSGGTIAGISLGSWLGALKAKVHAFSVCDDPDYFYDFVQGLLDGLQAGVNSRDIVSI
HNAKGKGYAMNTSEELKFLKDTASATSVILDPVYSGKAAYGLINEMTKDPKSWEGKKILF
IHTGGLLGLYDKVDQMASLMGNWSRMDVQESVPRKEGVGKMF*
>BolC6t36164H
MAPLKITGAVVAAATMVMLSYCYLGFFRFSDKLESCSSSKKKTKTKTKKEKLSTRNGLVD
AIGNTPLIRINSLSEATGCEILGKCEFLNPGGSVKDRVAVKIIEEALESGKLFPGGIVTE
GSAGSTAISLATVAPAYGCQCHVVIPDDAAIEKSQIIEALGATVERVRPVSITHKDHFVN
IARRRADEAKELASSKRRLASGINVAHQGKTNGCTAEEEKEPSLFSESVTGGFFADQFEN
LANYRAHYEGTGPEIWQQTHGNIDAFVAAAGTGGTLAGVSRFLQEKNEKVKCFLIDPPGS
GLYNKVTRGVMYTREEAEGRRLKNPFDTITEGIGINRLTQNFLMAKIDGGFRGTDKEAVE
MSRFLLKKDGLFVGSSSAMNCVGAVRVAQALGPGHTIVTILCDSGMRHLSKFHDPQYLAL
YGLTPTAVGLEFLGIK*
>BolC6t37030H
MTSQLLLPPNPFTRPVSAKVFLTGDDLTLIRKSNQATRVSNGFSLRAKAALRSNHSSSVE
IPNQWYNLIADLSVKPPPPLHPKTLEPIKPEDLSHLFPNELIKQEATLERFIDIPEEVLE
IYKLWRPTPLIRAKRLEKLLQTPARIYFKYEGSSPAGSHKPNTAVPQAYYNAKEGVKNVV
TETGAGQWGSSLAFASSLFGLDCEVWQVANSYHQKPYRRLMMQTWGAKVHPSPSDLTEAG
RKILESDPSSPGSLGIAISEAVEVAARNEDTKYCLGSVLNHVLLHQTVIGEECIKQMEDF
GETPDVIIGCTGGGSNFAGLSFPFIREKLKGNISPVIRAVEPSACPSLTKGVYAYDFGDT
AGLTPLMKMHTLGHDFIPDPIHAGGLRYHGMAPLISHVYEQGFMEAISIPQIECFQGAIQ
FARTEGIIPAPEPTHAIAATIREALRCKETGEAKVILMAMCGHGHFDLSSYDKYLKGELV
DLSFSEDKIRESLSKVPHVV*
>BolC6t37034H
MTSQLLLPPNPFTRPVSAKVFLTDDDLTLKRKSNQATRVSNGFSLRAKAALRSNHSSSVE
IPNQWYNLTADLSVKPPPPLHPKTLEPIKPEDLSHLFPNELIKQEATLERFIDIPEEVLE
IYKLWRPTPLISKELEKLLQTPARIYFKYEGGSPAGSHKPNTAVPQAYYNAKEGVKNVVT
ETGAGQWGSSLAFASSLFGLDCEVWQVANSYHQKPYRRLMMQTWGAKVHPSPSDLTEAGR
KILESDPSSPGSLGIAISEAVETVIGEECIKQMEDFGETPDVIIGCTGGGSNFAGLSFPF
IREKLKGNISPVIRAVEPSACPSLTKGVYAYDFGDTAGLTPLMKMHTLGHDFIPDPIHAG
GLRYHGMAPLISHVYEQGFMEAISIPQIECFQGAIQFARAEGIIPAPEPTHAIAATIREA
LRCKETGEAKVILMAMCGHGHFDLSSYDKYLKGELVDLSFSEDKIRESLSKVPHVV*
>BolC7t40746H
MPLHLCLRHCTSDYSACVSSFMFWDARNTRKAGGDIMGVDVLLLDQKRLGKKRIIAETGA
GQHGVATATVCAQYGLECIISMGAQDMERQALNVFRMRLLGAEVRGVHSGTATLKDATSE
AIRDWVTNVETTHCILGSVAGPHPYPMMVRDFHAVIGKETRRQALEKWGGKPDVMVACVS
GGSNAMGLFHEFVDDAEVRMIGVEAAGFGLDSGKHAAPLTKGDVGVLHGAMSYLLQDDDG
QIIEPQLDYPGVGPEHSFLKDMGRAEYYSVTDEEALEVKTCSLTRRFVAFNRVSRLQGII
SALETSHALPHLEKLCPTLPDGARVVLNFSGRGHKDVQTVAKYLEV*
>BolC7t44578H
MEDRCLIKNDVTELIGNTPMVYLNKIADGCVARIAAKLEMMEPCSSIKDRIAYSMIKDAE
DKGLITPGESTLIEPTAGNTGIGLACIGAARGYKVIILMSSSMSLERRIILRALGAELHL

TDRSIGFKGMLEKTEEMLSKTSGGFVPQQFENPSNPEIHYRTTGPEIWRDSAGKVDILVA
GVGTGGTVTGVGKFLKEMNQNIKVCAVEPTESPVLSGGEPGSHLIQGIGAGIIPTNLDLS
IVDEIIQVTGEEATETAKLLALKEGLLVGISSGATAAAALKVAKRPENAGKLIVVIFASG
GERYLSTKLFDSVRYEAENLQIE*
>BolC7t44816H
MGAQDMERQALNVFRMRLLGAEVRGVHSGTATLKDATSEAIRDWVTNVETTHYILGSVAG
PHPYPMMVRDFHAVIGKKTSRQAWEKWGGKPDVLVACVGGGSNAMGLFHEFVDDKEVRMI
GVEAAGFGLDSGKHAAPLTKGDVGVLHGAMSYLLQDDDGQIIEPQCRVRANQNMPNHKLD
YPGVGPEHSFFKDMGWAEYYSVTDEEALEVKTCSLTRRFVAFNRVSRLEGIISALETSHA
LAHLEKLCPKLPDGARVVLNFSGRGDKDDQTVAKYLEV*
>BolC7t46009H
MATSGTASTFRPSVSASSRLTHLRSSPFKVPNFTPLPSSRSHSFSVSCTIAKDPTFLMAE
AEKTKAAGSDPTLWKRPDSFGRFGKFGGKYVPETLMHALSELETAFYSLATDDDFQRELA
GILKDYVGRESPLYFAERLTEHYRRENGEGPLIYLKREDLNHTGAHKINNAVAQALLAKR
LGKKRIIAETGAGQHGVATATVCARFGLQCIIYMGAQDMERQALNVFRMRLLGAEVRGVH
SGTATLKDATSEAIRDWVTNVETTHYILGSVAGPHPYPMMVRDFHAVIGKETRRQAMEKW
GGKPDVLVACVGGGSNAMGLFHEFVDDTEVRMIGVEAAGFGLDSGKHAATLTKGDVGVLH
GAMSYLLQDDDGQIIEPHSISAGLDYPGVGPEHSFLKDMGRAEYYSVTDEEALEAFKRVS
RLEGIIPALETSHALAHLEKLCPTLPDGARVVLNFSGRGDKDVQTAIKYLEV*
>BolC8t48250H
MASRIAKDVTELIGNTPLVYLNNVAEGCVGRVAAKLEMMEPCSSVKDRIGFSMISDAEKK
GLIKPGESVLIEPTSGNTGVGLAFTAAAKGYKLIITMPASMSVERRIILLAFGVELVLTD
PAKGMKGAIAKAEEILAKTPNGYMLQQFENPANPKIHYETTGPEIWKGTDGKIDGFVSGI
GTGGTITGAGKYLKEQNPNVKLYGVEPIESAILSGGKPGPHKIQGIGAGFIPSVLEVDLI
DEVVQVSSDESIDMARLLALKEGLLVGISSGAAAAAAIKLAKRPENAGKLFVAVFPSFGE
RYLSTVLFDATRKEAESMTFQACIVSP*
>BolC8t49104H
MSSTKIQLRWQPLPRVPARNHRMINSVVFGVPIKSHHRVSDVHSRTNGPSFGRFGRFGGK
FVPETLMSPLRDLEEELDFVLSDHEFQAEFTTALRDYVGRETPLYFAERLTEHYRNKCRT
TGDGPEIYLKREDLGHSGSHKMNNALAQAMIARRLGCSRVVAATGAGQHGVATAAACAKL
SLECTVFMGTTDIEKQSSNVLSMKLLGAQKSFITNSFSEKKILLQIIFPDTIKYSSTNIK
FVSSLEGTFQDASSEAIRNWVENLQTTYYLSGTVVGPHLSPVMVREFQSVIGKETRKQAK
RLWGGKPDVLVAFVGSGSNALGLFHEFVGDEDVRLVGVEAAGLGLDSGKHSATLAVGDVG
VYHGSIELLVAR*
>BolC8t49108H
MSSTKIQIRGQPLSKVLTRNHGMINSVVCGVPIKRHHRVSNVLRTSDPPLGSVPTRTDES
QFLRGDGNGRFGRFGGKFVPETLISPLRDLEDEFNFVLNDHEFQEELTTALRDYVGRETP
LYFAGRLTEHYKNISQTTGGGPEIYLKREDLSHCGSHKINNALGQAMIARRLGCKRVVAA
TGAGQHGVATAAACAKFSMECTVFMGTADIEKQSSNVLSMKLLGAQVKSVEGTFKDASSE
AIRNWVGNLETTYYLSGTVVGPHPSPLMVREFQSVIGKETRRQAKQLWGGKPDVLVACVG
SGSNALGLFHEFLGDEDVRLVGVEAAGLGLDSGKHSATLAVGDVGVYHGSMSYLLQDDQG
QILKPHSIGVGLEYPGVGPEISFLKESGRSEFYTATDQEAVQACMLLSRLEGIIPALEAS
HALAFLDKLVPTLRDGAKVVVNCSGRGDKDLDTLIQRGMPSSLC*
>BolC8t51214H
MATIWRRLLKTETIPRISQSTRKLFSSDGSSSFADRLRNLPKEFPATQAKRDASLLIGRT
PLVFLNRVTEGCGAYIAAKQEHFQPTCSVKDRPALAMVADAEKKNLITPGKTTLIEPTSG
NMGISMAFMAAMKGYKIIMTMPSYTSLERRVTMRSFGAELVLTDPTKGMGGTVKKAYDLL
ESTPDAHMLQQFANPANTQIHYDTTGPEIWEDTLGNVDIFVMGIGSGGTVSGVGQYLKFK
NPNVKIYGVEPAESNILNGGKPGAFYLALNFGLGPHAITGNGVGFKPDILDMDVMESVLE
VSSEDAIKMARELALKEGLMVGISSGANTVAAIRLAKMPENKGKLIVTIHASFGERYLSS
ILFDELRKEAEAMKPVSVD*
>BolC9t53481H
MFIVAFKELRACYVLCYSRNNEFSRQTSMEERYMIKNDVTELIGNTPMVYLNKIVDGCVA

RVAAKLEMMEPCSSIKDRIAYSMIKDAEDKGLITPGKSTLIEATGGNTGIGLASIGAARG
YRVILLMPSTMSLERRIILRALGAEVHLTDMNIGIKGMLEKAEEILSKTPGGYIPHQFLN
PENPEIHYRTTGPEIWRDSAGEVDILVAGAGTGGTVSGTGRFLKKMNKNIKVCVVEPTES
AVLSGGEPGPHLIQGIGPGVIPTNLDLSIVDEVIQVTGEEAIETAKLLALKEGLLVGISS
GAAAAAALKVAKRPENAGKLIAVLFPSGGERYLSTKLFDSVRFEAENLPVE*
>BolC9t54277H
MSWTKIQVRGQPVPRVPARNHRMINSFVCGVSIKSHHRVSNVLRTNGSPLGSVPIRTTES
QFLRGDVNGRFGRFGGKFVPETLMSLLRDLEDEFNFVLSDHEFQEELTTALRDYVGRETP
LYFAGRLTEHYKSISRTIGDGPEIYLKREDLSHCGSHKINNALAQAMIARRLGCSRVVAA
TGAGQHGVATAAACAKFSVECIVFMGTADKEKQFSNILSMKLLGAQVKSVEGTFKDASSE
AIRNWVGNLETTYYLSGTVVGPHPSPLMVREFQSVIGKETRRQANQLWGGKPDVLVACVG
SGSNALGLFHEFVGDEDVRLVGIEAAGLGLDSGKHSATLAVGDVGVYHGSMSYLLQDDEG
QILKPHSVGVGLEYPGVGPEISFLKETGRAEFYTATDQEAIQACRLLSRLEGIIPALEPS
HALAFLDKLVPTLRDGAKVVVNCSGRGDKDLDTLIQRGLPSSLC*
>BolC9t54359H
MTSCSFSSSSLFSFNLPNQNSLHRRPPTLPRHAIVSSTDGSSNGASSSSSPTVKRTRTED
NIRDEARRHRSASANPFSARYVPFNAPPGSTESYSLDEVVYRSESGGLLDVQHDLDALRS
HDGAYWRNLFDSRVGKTKWPYGSGVWSKKEWVLPEIDDDDIVSAFEGNSNLFWAERFGKT
FLGMNDLWVKHCGISHTGSFKDLGMTVLVSQVNRLRKMNKPVVGVGCASTGDTSAALSAY
CASAGIPSIVILPANKISMAQLVQPIANGAFVLSIDTDFDGCMKLIREITSELPIYLANS
LNSLRLEGQKTAAIEILQQFNWQVPDWVIVPGGNLGNIYAFYKGFKMCQDLGLVDRIPRL
VCAQAANANPLYLHYKSGWKEFKPVKANATFASAIQIGDPVSIDRAVYALRNCDGIVEEA
TEEELMDAMAQADSTGMFVCPHTGVALTALFKLRSQGVIAPTDRTVVVSTAHGLKFTQAK
IDYHSKAIPDMACRFSNPPVEVKADFGAVMDVLKVYLGSEELRENIQYIYNFSFFSDETE
SYFTYSLYNHANVSGQIKQFTWLNNNNQWNLFWSQLRQQCQVHAYCGSFGICNDKSQPFC
QCPQGFRPVSQKDWDLKDYSAGCVRSTELQWSR*
>BolC9t57401H
MAASGTVASFRTSVSSSPQFTHLRSPSKALKFTPLPSSRSRPSFSVSCTIAKDPPVLMSA
GSDPTLWQRPDSFGRFGKFGGKYVPETLMHALSELETAFHSLATDDDFQRELAGILKDYV
GRESPLYFAERLTEHYRRENGEGPLIYLKREDLNHTGAHKINNAVAQALLAKRLGKKRII
AETGAGQHGVATATVCARFGLECIIYMGAQDMERQALNVFRMRLLGAEVRAVHSGTATLK
DATSEAIRDWVTNVETTHYILGSVAGPHPYPMMVRDFHAVIGKETRRQALEKWGGKPDVL
VACVGGGSNAMGLFHEFVDDTEIRLIGVEAAGFGVDSGKHAATLTKGDVGVLHGAMSYLL
QDDDGQIIEPHSISAGLDYPGVGPEHSFLKDMGRAEYFSVTDEEALEAFKRVSRLEGIIP
ALETSHALAHLEKLCPTLPDGSRVVLNFSGRGDKDVQTVAKYLEV*
