# Supplementary figures and images for: Identification and expression analysis of the cysteine synthase (CSase) gene family in Brassica napus L. under abiotic stress
Source: BMC Plant Biol. 2025 Jun 5;25:770. doi: 10.1186/s12870-025-06532-8 (PMC12139137; doi:10.1186/s12870-025-06532-8)

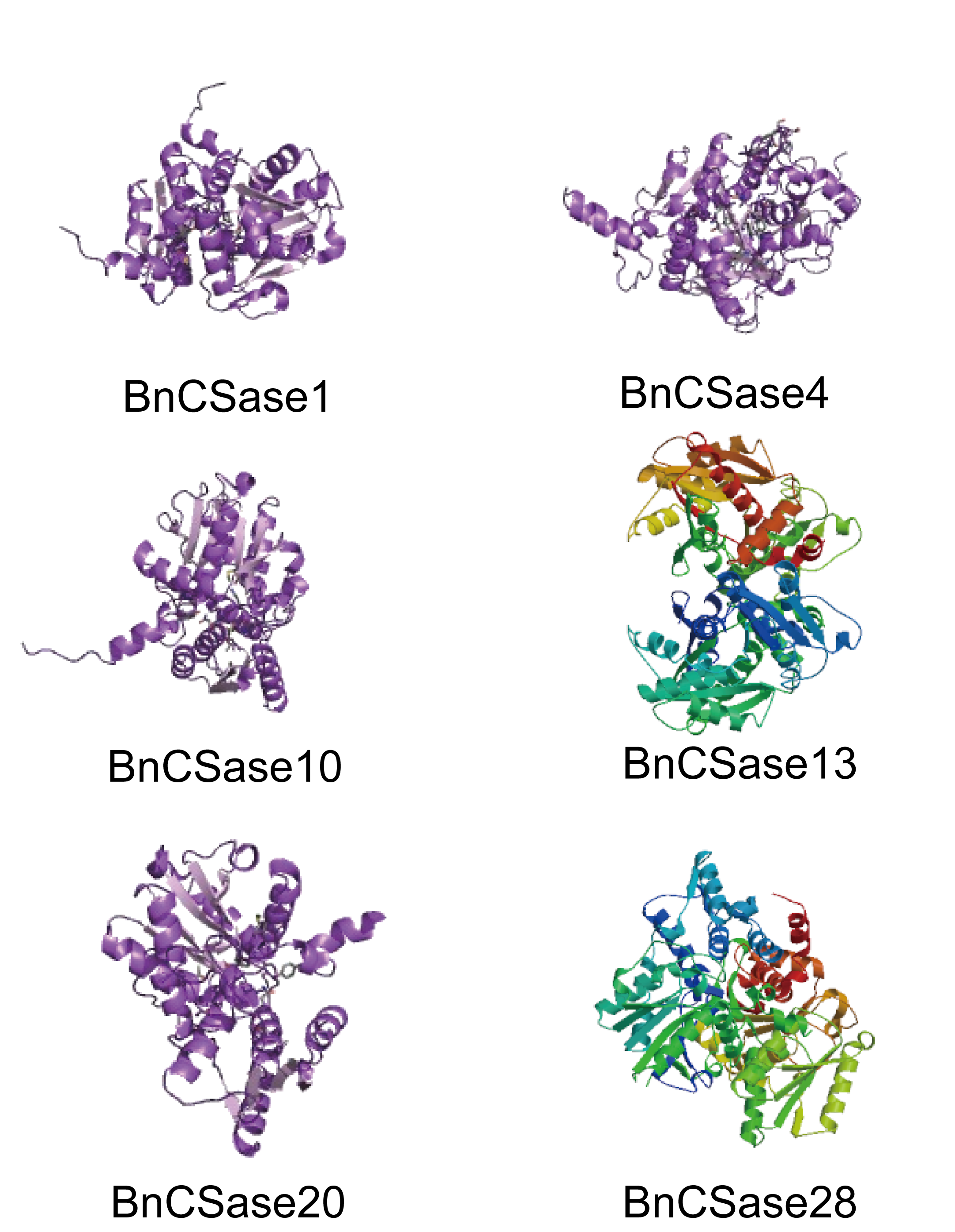

Supplement: Supplementary file 2 — Supplementary Material 2 [file 12870_2025_6532_MOESM2_ESM.tif]
